# A new hadrosauroid (Dinosauria: Ornithopoda) from the Late Cretaceous Baynshire Formation of the Gobi Desert (Mongolia)

**DOI:** 10.1371/journal.pone.0208480

**Published:** 2019-04-17

**Authors:** Khishigjav Tsogtbaatar, David B. Weishampel, David C. Evans, Mahito Watabe

**Affiliations:** 1 Institute of Paleontology and Geology, Mongolian Paleontological Center, Mongolian Academy of Sciences, Ulaanbaatar, Mongolia; 2 Center for Functional Anatomy and Evolution, Johns Hopkins University, Baltimore, Maryland, United States of America; 3 Department of Natural History, Royal Ontario Museum, Toronto, Ontario, Canada; 4 Department of Geosciences, Faculty of Science, Osaka City University, Osaka Prefecture, Osaka, Japan; Universidad de Chile, CHILE

## Abstract

A new genus and species of non-hadrosaurid hadrosauroid, *Gobihadros mongoliensis*, is described from a virtually complete and undeformed skull and postcranial skeleton, as well as extensive referred material, collected from the Baynshire Formation (Cenomanian-Santonian) of the central and eastern Gobi Desert, Mongolia. *Gobihadros mongoliensis* is the first non-hadrosaurid hadrosauroid from the Late Cretaceous of central Asia known from a complete, articulated skull and skeleton. The material reveals the skeletal anatomy of a proximate sister taxon to Hadrosauridae in remarkable detail. *Gobihadros* is similar to *Bactrosaurus johnsoni* and *Gilmoreosaurus mongoliensis*, but can be distinguished from them in several autapomorphic traits, including the maximum number (three) of functional dentary teeth per tooth position, a premaxillary oral margin with a ‘double-layer morphology’, and a sigmoidal dorsal outline of the ilium with a well-developed, fan-shaped posterior process. All of these characters in *Gobihadros* are inferred to be convergent in Hadrosauridae. Phylogenetic analysis positions *Gobihadros mongoliensis* as a *Bactrosaurus*-grade hadrosauromorph hadrosauroid. Its relationship with Maastrichtian hadrosaurids from Asia (e.g., *Saurolophus angustirostris*, *Kerberosaurus manakini*, *Wulagasaurus dongi*, *Kundurosaurus nagornyi*) are sufficiently distant to indicate that these latter taxa owe their distribution to migration from North America across Beringia, rather than having a common Asian origin with *Go*. *mongoliensis*.

## Introduction

Ornithopod dinosaurs have been known since before the concept of Dinosauria originated [1, 2]. These herbivores have traditionally been grouped into several lower taxa: Heterodontosauridae, Hypsilophodontidae, Iguanodontidae, and Hadrosauridae. In recent years, heterodontosaurids have been determined to be basal ornithischians [3, 4], hypsilophodontids are now widely regarded as paraphyletic [3–6], and Iguanodontidae is a more restricted clade [7–13]. Of particular importance to the understanding of ornithopod evolution is the plethora of new species of hadrosauroids phylogenetically nested between *Iguanodon* and the origin of Hadrosauridae that have been described in the last two decades (e.g., [14–25]). Most of these new taxa are known from the Early Cretaceous of China, although a few are known from the Late Cretaceous. These new discoveries, together with a resurgence of phylogenetic interest in the group, have resulted in a better understanding of the origin of Hadrosauridae (= the least inclusive clade containing *Hadrosaurus foulkii* Leidy and *Lambeosaurus lambei* Parks, sensu [26]), but have produced a series of conflicting or poorly-resolved phylogenetic hypotheses of the relationships within Hadrosauroidea (e.g., [9, 11–13, 18, 23, 26–29]).

These new discoveries also reveal that knowledge of hadrosauroid evolution remains poor in the pre-Campanian part of the Late Cretaceous. This time interval is important in iguanodontian evolution since it is when Hadrosauridae originated [26, 29], and new material from this time period may ultimate help resolve long-standing questions including the biogeographic origin of the clade (e.g., [30]), and the order and timing of derived trait acquisitions that define it. The present paper describes a new, derived hadrosauroid taxon, *Gobihadros mongoliensis* gen. et sp. nov., which is the only non-hadrosaurid taxon known from the early Late Cretaceous (Santonian) of the Gobi Desert in Mongolia. The exquisitely preserved material that comprises the hypodigm of this new taxon was collected by the Mongolian Palaeontological Center-Hayashibara Museum of Natural Sciences joint paleontological expedition from 1993–2004 [31, 32]. In addition to revealing the anatomy of both the cranial and postcranial skeleton of a proximate sister taxon to Hadrosauridae in remarkable detail, this study also analyzes the position of *Go*. *mongoliensis* in the context of non-hadrosaurid hadrosauroids and discusses its biogeographic significance.

## Materials and methods

### Geological setting

**Fig 1:**
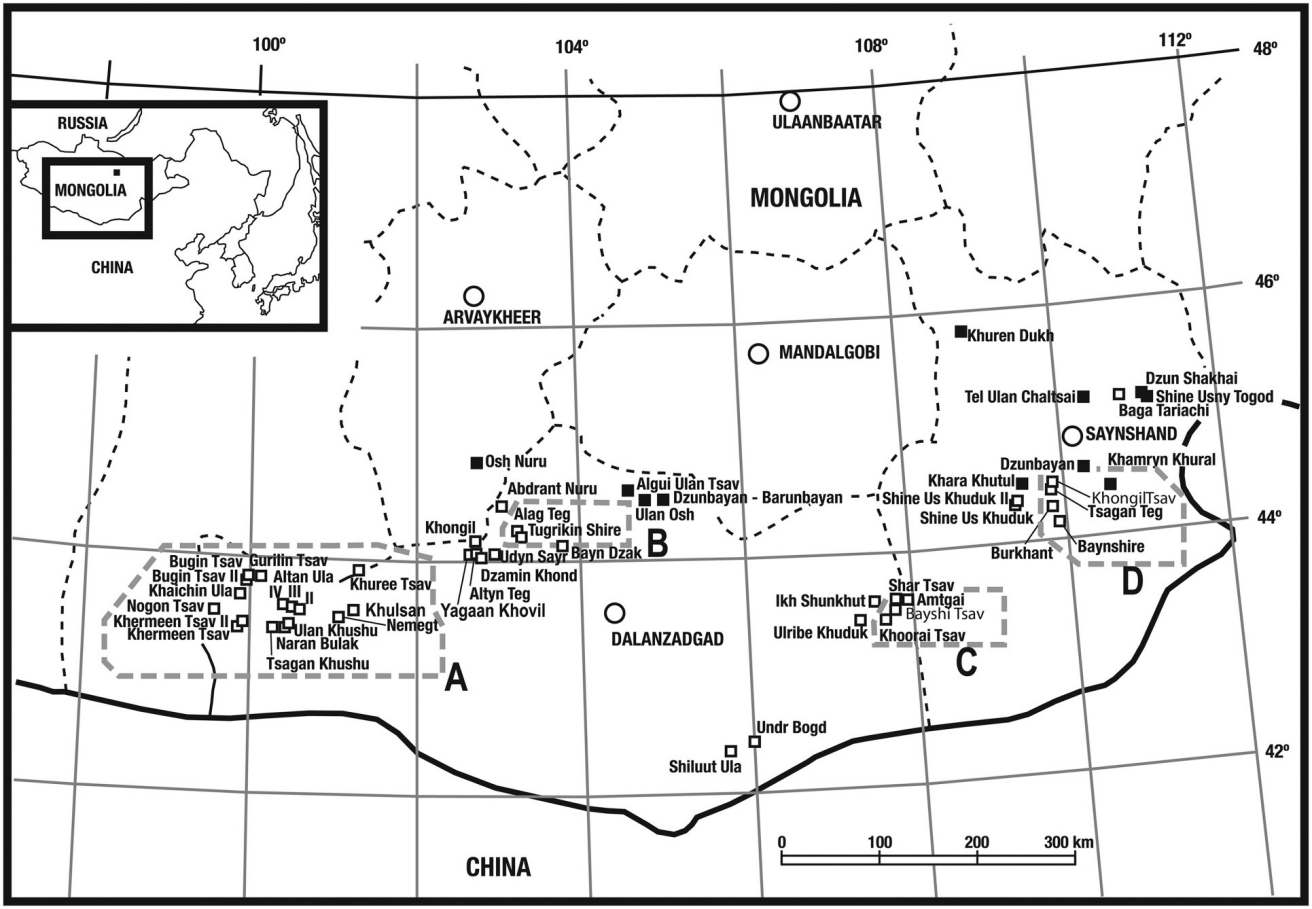
Map of Cretaceous-aged dinosaur fossil localities of Mongolia. *Gobihadros mongoliensis* was collected from Bayshin Tsav in Area C. Open squares indicate Late Cretaceous sites, solid squares represent Early Cretaceous localities. Abbreviations: A, Localities of Western Gobi Desert in Mongolia, mainly group of localities of Nemegtian age (early Maastrichtian), Late Cretaceous; B, Localities of Central Gobi Desert in Mongolia, mainly Djadokhtian age (Campanian), Late Cretaceous; C & D- Localities of Eastern Gobi Desert in Mongolia, mainly Baynshirenian age (Cenomanian-Santonian), Late Cretaceous. Figure has been modified from Tsogtbaatar et al. 2014, Figure 1 [24].

Bayshin Tsav, where the holotype material of *Gobihadros mongoliensis* was collected, is a fossiliferous locality in the eastern part of the South Gobi Aimag, situated ca. 95 km SE of Manlai Somon (Fig 1). Strata at this locality are divided into five sub-localities (areas) by distribution of outcrop: Bayshin Tsav I, II, III, IV, and V. All of the sub-localities have yielded well-preserved material including an articulated skeleton, associated specimens, and isolated bones. The fossil-bearing beds, bluish-white to yellowish-brown medium-grained sandstone and dark-to-light gray mudstone strata, form small hills and low cliffs, with the total thickness of the section in the area only approximately 20 m. The beds form heterolithic interbedded units, characteristic of point bar deposits [33–35]. Articulated and isolated bones of hadrosauroids and other vertebrates can be found in all of these lithological units. Some of the sandstone layers include aggregations of isolated bones of various vertebrate taxa such as dinosaurs and turtles; these are considered bonebeds. Similar alternating beds at this locality yield fully and partially articulated skeletons, and parts of hadrosauroid skeletons.

Khoorai Tsav is a locality situated 3 kilometers southwest of Bayshin Tsav in the South Gobi Aimag. The widely distributed fossiliferous beds form cliffs along narrow gorges that run from North to South. These outcrops extend continuously to the area of Bayshin Tsav, and have a similar total thickness of 20 m. The area has the same lithological conditions as Bayshin Tsav and is also rich in hadrosauroid fossils. We consider these fossil bearing beds at Bayshin Tsav and Khoorai Tsav as belonging to the same formation.

Another locality, Khongil Tsav, is in the Eastern Gobi Aimag, situated 16 km WSW of the town of Dzunbayan Somon (Fig 1). The fossil bearing beds form complicated cliffs facing to the east and south and extend 6 km from north to south. The exposed fossil bearing strata are considerably thicker here, with a total thickness of at least 50 m. The lower and upper boundaries of the beds cannot be observed [33].

Bayn Shire is a locality in the Eastern Gobi Aimag, situated 26 km southwest of Dzunbayan Somon (Fig 1). The fossil bearing beds are distributed forming the cliffs facing to the east and south, in a large mesa, with the total thickness of the fossil beds (56 m) similar to Khongil Tsav. The lower and upper boundaries of the beds are also not observed [35].

The fossiliferous beds in Bayshin Tsav, together with the beds of other localities such as Khongil Tsav and Bayn Shire, are thought to be late Cenomanian to Santonian in age based on their molluscan, ostracode, and vertebrate faunas and named the Baynshire Svita [36–38]. Jerzykiewicz and Russell [39] correlated the Baynshire Svita with the Iren Dabasu Formation based on its fauna from Inner Mongolia. Hicks et al. [40] carried out paleomagnetic analyses on the fossiliferous beds at Bayn Shire, which is one of the stratotype localities of the Baynshire Svita. Their analyses have limited the chronological range of the Baynshire Svita to Cenomanian-Santonian.

### Permits

Field permits from the Mongolian government were obtained to conduct the fieldwork that resulted in the collection of the specimens described herein. No further permits were required for the described study, which complied with all relevant regulations.

### Nomenclatural acts

The electronic edition of this article conforms to the requirements of the amended International Code of Zoological Nomenclature, and hence the new names contained herein are available under that Code from the electronic edition of this article. This published work and the nomenclatural acts it contains have been registered in ZooBank, the online registration system for the ICZN. The ZooBank LSIDs (Life Science Identifiers) can be resolved and the associated information viewed through any standard web browser by appending the LSID to the prefix “http://zoobank.org/”. The LSID for this publication is: urn:lsid:zoobank.org:pub:520779DB-3095-42CE-B912-A933CDF41922. The electronic edition of this work was published in a journal with an ISSN, and has been archived and is available from the following digital repositories: PubMed Central, LOCKSS

**Institutional Abbreviations:** MPC: Mongolian Palaeontological Center, Ulaanbaatar.

## Results

### Systematic Palaeontology

ORNITHISCHIA Seeley, 1887 [41]

ORNITHOPODA Marsh, 1881 [42]

IGUANODONTIA sensu Sereno, 1998 [43]

HADROSAUROIDEA sensu Sereno 1998 [43]

*Gobihadros* nov. gen. urn:lsid:zoobank.org:act:38EE8AD7-AD50-44BF-B31D-B2675456556A

**Etymology.** Hadrosauroid from the Gobi Desert of Mongolia.

**Generic diagnosis.** As per species diagnosis.

*Go*. *mongoliensis* nov. sp.

urn:lsid:zoobank.org:act:2DB42EE7-6A64-4D64-AA19-E5D3453BF99C

**Holotype.** MPC-D100/746, a complete, nearly articulated skeleton from sub-locality I of Bayshin Tsav.

**Referred material.** MPC-D100/763 (complete articulated skull and manus from Bayshin Tsav, Sub-locality unknown), MPC-D100/710, 711, 712, 713, 714, 715, 716, 717, 718, 719, 720, 721, 722, 723, 724, 725, 726, 727, 728 (all from Sub-locality I, Bayshin Tsav), MPC-D100/729, 730, 731, 732, 734, 735, 736, 737, 738, 740, 741, 742 (all from Sub-locality IV, Bayshin Tsav), MPC-D100/743, 744 (Khongil Tsav), MCP-D100/747 (Sublocality I, Bayshin Tsav), MPC-D100/748 (Bayn Shire), MPC-D100/749 (Bayn Shire), MPC-D100/750 (Bayn Shire), MPC-D100/752 (Sublocality I, Bayshin Tsav), MPC-D100/753 (Sublocality V, Bayshin Tsav), MCP-D100/754, 755, 760 (from Khoorai Tsav), MPC-D110/756 (Sublocality III, Bayshin Tsav), MPC-D100/761, 762 (Sublocality IV, Bayshin Tsav).

**Etymology.** From Mongolia.

**Locality and horizon.** Bayshin Tsav (South Gobi Aimag); Khoorai Tsav (South Gobi Aimag); Khongil Tsav (East Gobi Aimag); Baynshire Formation (Cenomanian-Santonian), Upper Cretaceous.

**Diagnosis.** Small hadrosauroid that differs from all other non-hadrosaurid hadrosauroids (including *Bactrosaurus* and *Gilmoreosaurus*) in the presence of a premaxilla with a ‘double-layer’ oral margin and up to three functional teeth in the dentary tooth row (both convergent in Hadrosauridae). *Gobihadros* differs from *Bactrosaurus johnsoni*, *Probactrosaurus gobiensis*, *Eolambia caroljonesi*, *Claosaurus agilis*, *Tethyshadros insularis*, in the sigmoidal dorsal outline of the ilium and the greater lateral expression of the supracetabular crest. *Gobihadros* differs from *T*. *insularis*, *Plesiohadros djaktaensis*, and Hadrosauridae in the possession of a spike-like manus digit 1.

### Description

This description focuses on two excellently preserved specimens of *Gobihadros mongoliensis*. One is a complete and uncrushed skull (MPC-D100/763), while the second—the holotype (MPC-D100/746)–is a virtually complete skull and postcranial skeleton. Except for the skull, this latter specimen is fully articulated. The skull was found closely associated, but not articulated except for the braincase, the right and left mandibles, and the right and left prefrontals and lacrimals. Only minimal distortion is present in both cranial and postcranial elements. The available specimens represent a range of sizes and presumed ontogenetic stages from subadult to adult. The osteology described here is consistent across the known size variation and is hypothesized to characterize the adult morphology. A comparative table of measurements for select Mongolian hadrosauroids is provided in Supporting Information S1 File.

#### Skull

**Fig 2:**
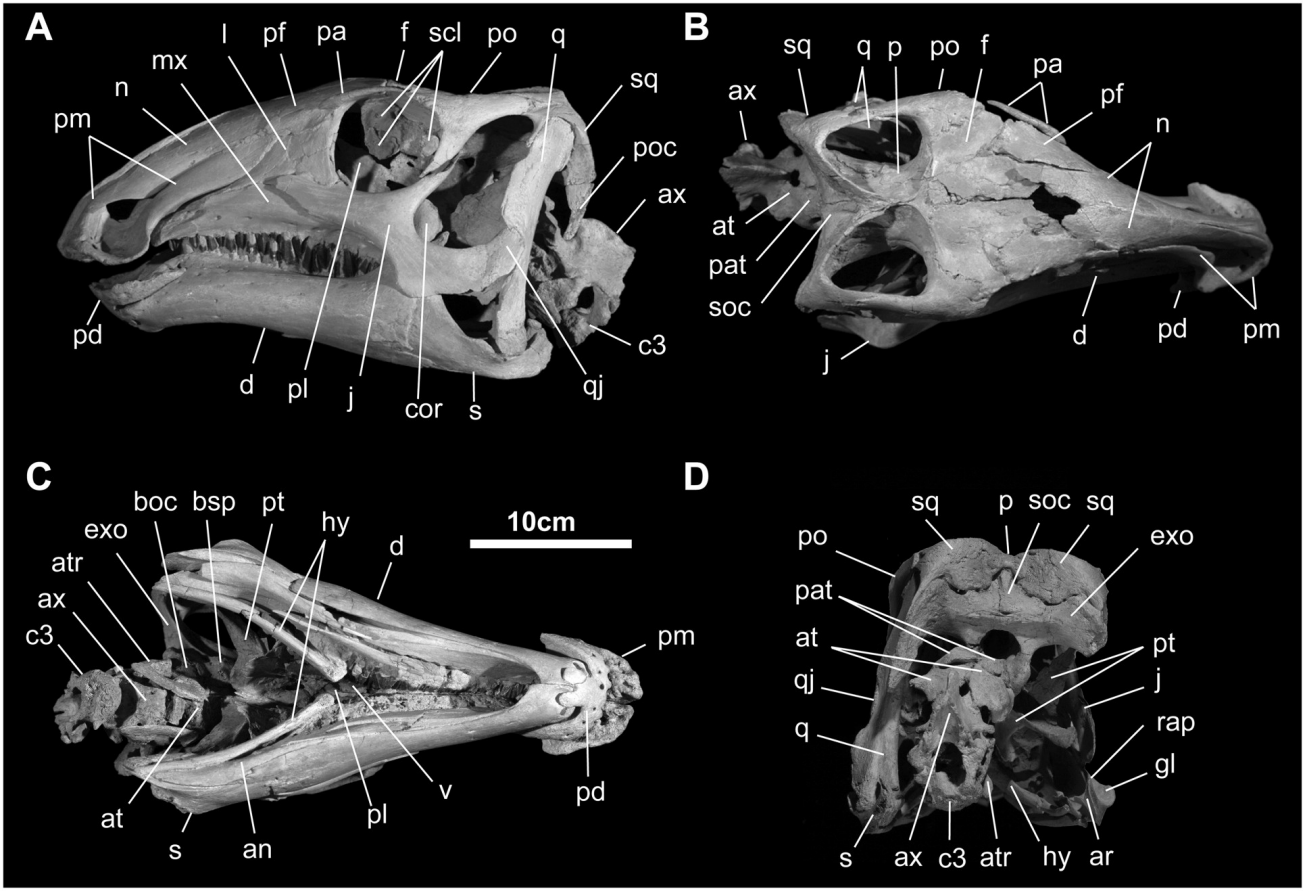
Skull and mandible of *Gobihadros mongoliensis*. Skull and mandible (MPC-D100/763) in left lateral (A), dorsal (B), ventral (C), and posterior (D) views. Abbreviations: an, angular; ar, articular; at, atlas; atr, atlantal rib; ax, axis; boc, basioccipital; bsp, basisphenoid; cop, coronoid process; c3, 3rd cervical vertebra; d, dentary; exo, exoccipital; f, frontal; gl, glenoid for the lateral quadrate condyle; hy, hyoid; j, jugal; l, lacrimal; mx, maxilla; n, nasal; p, parietal; pa, palpebral; pat, proatlas; pd, predentary; pf, prefrontal; pl, palatine; pm, premaxilla; po, postorbital; poc, paroccipital process; pt, pterygoid; q, quadrate; qj, quadratojugal; rap, retroarticular process; s, surangular; scl, sclerotic ring; soc, supraoccipital; sq, squamosal; v, vomer.

In lateral view, the skull of MPC-D100/763 is 294 mm long, rising gradually from its rostral tip to the orbits, and the quadrate height is 154 mm (Fig 2). Equivalent measurements of MPC-D100/746 are 226 mm and 115 mm. The transverse width of the postorbital region of the skull in dorsal view is broad; width is maintained from the orbit to the head of the quadrate. The external naris is relatively short (20% basal skull length) and the dorsal and caudoventral premaxillary processes do not meet caudal to external nares. Instead, its v-shaped caudal margin is formed by the nasal. The caudalmost apex of the external naris is formed equally by the nasal (dorsally) and the premaxilla (ventrally). In its proportions and composition, it differs from *Tenontosaurus tilleti* Ostrom, *Jinzhousaurus yangi* Wang & Xu, *Iguanodon bernissartensis* Mantell, and *Altirhinus kurzanovi* Norman, but is similar to *Telmatosaurus* and *Probactrosaurus* [7, 14, 44–46]. The antorbital foramina and fossa appear to be absent (similar to *Eolambia*, but different from many other non-hadrosaurid hadrosauroids; [10], although a small opening along the jugal-lacrimal suture may well be the last vestige of this system opening onto the lateral surface of the skull. The nearly circular orbits are 18% basal skull length. The supratemporal fenestra is nearly subequal in transverse (38 mm) and rostrocaudal (33 mm) dimensions. The infratemporal fenestra is somewhat trapezoidal, 80 mm in maximal length and wider ventrally than dorsally. The foramen magnum is subcircular, slightly prolonged and pointed ventrally. A modest fontanel with irregular edges, found between the paired nasals and frontals, testifies to the immature ontogenetic status of this individual. The vomer, ectopterygoids, and stapes appear to be missing.

**Fig 3:**
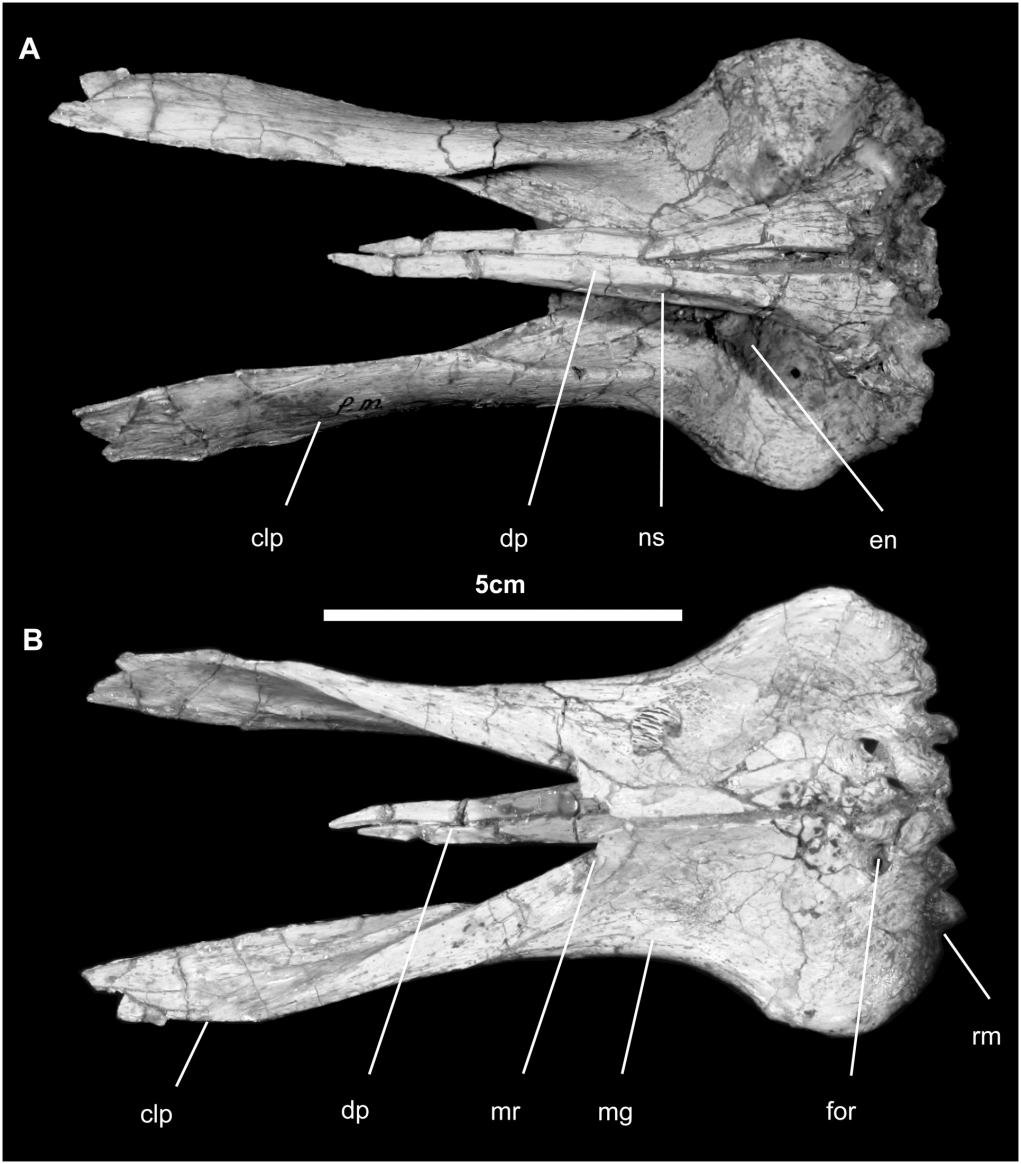
Premaxilla of *Gobihadros mongoliensis*. Premaxillae (MPC-D100/746, left and right) in dorsal (A) and ventral (B) views. Abbreviations: clp, caudolateral process; dp, dorsal process; en, external naris; for, premaxillary foramen; mg, maxillary groove; mr, midline recess for rostromedial process of the maxilla; ns, nasal suture; rm, rostral margin.

*Premaxilla* (Fig 3): The paired premaxillae were found in articulation and no attempts have been made to disarticulate them. In dorsal view, the oral margin is straight and bears a row of large denticles (three or four denticles per premaxilla), unlike in *Mantellisaurus*, *Ouranosaurus*, *Protohadros*, *Eolambia*, and *Dakotadon*, which have only two [10, 12, 47–50]. There is a second, more caudal (more oral) row of smaller denticulations separated from the aforementioned row by a deep sulcus bearing vascular foramina, as in hadrosaurids. The external surface of the oral region of the premaxilla is slightly rugose, indicating the presence of a keratinous rhamphotheca. More caudally, the premaxilla is expanded laterally less than twice the width of the premaxilla at its narrowest point. The lateral profile of the rostral margin of the premaxilla is slightly less than 70°. Although the dorsal process is slightly dorsoventrally crushed, it was very long, terminating 35 mm prior to the caudalmost extent of the caudolateral premaxillary process. There is no reflected rim around the oral margin, nor is there a circumnarial depression, which occurs in hadrosaurids. The narial fossa extends in front of and beneath the rostral and ventral margins of the external naris onto the expanded narial platform. A canal traverses the premaxilla from the rostral surface of the narial fossa to the front of the premaxillary palatal surface, 7 mm from the midline. There is no accessory narial foramen. The flat caudolateral process shrouds the upper articular surface with the maxilla, ending as it covers the front of the lacrimal. There is no outer (accessory) narial fossa. In ventral and caudal view, just beneath the ventral margin of the external naris is a small, shallow, but well-defined excavation that accommodates the styloid rostromedial process of the maxilla. Immediately caudolateral on the ventral surface is a larger, more rounded fossa into which the blunt rostrolateral process of the maxilla fits.

**Fig 4:**
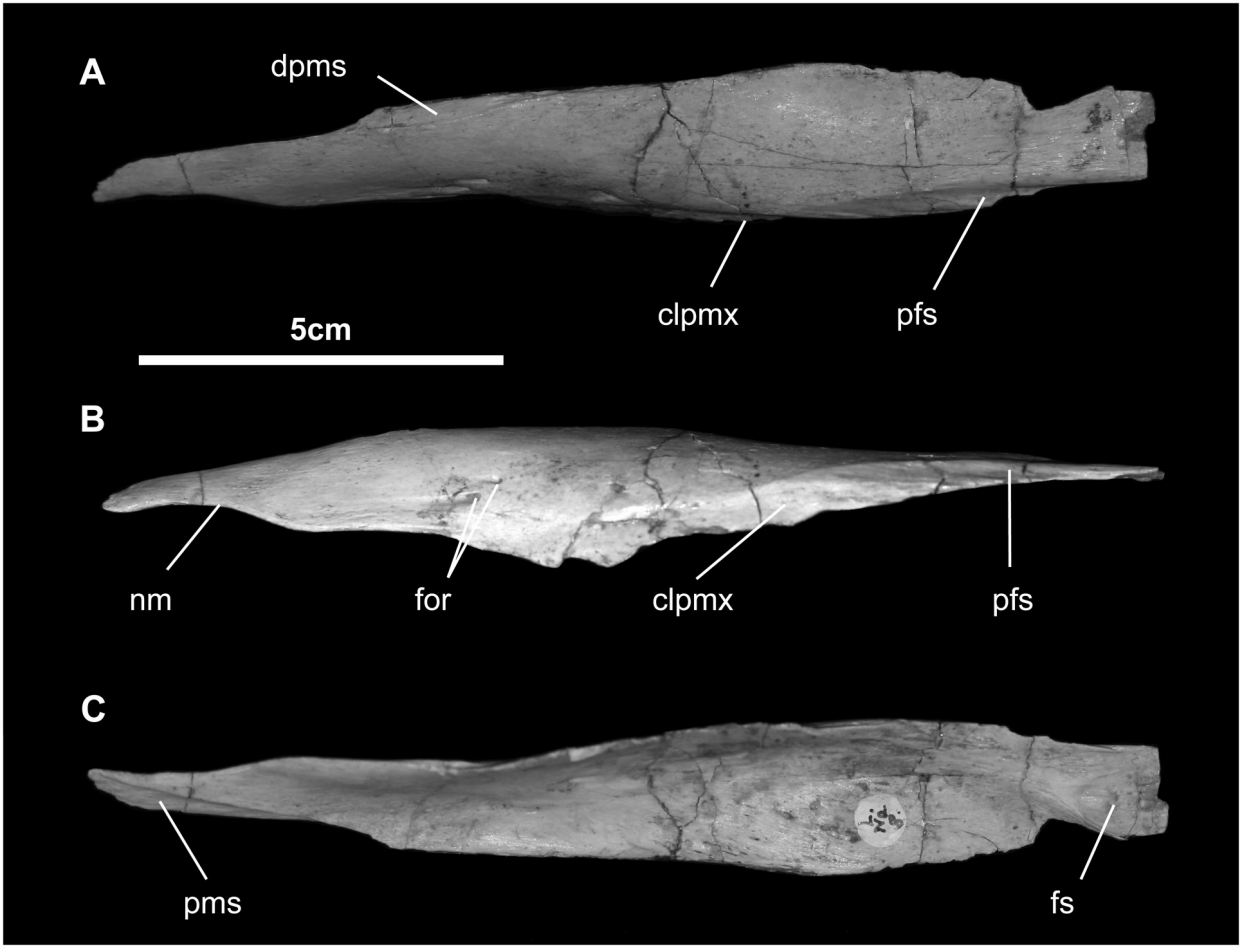
Nasal (MPC-D100/746, left) of *Gobihadros mongoliensis*. Nasal (MPC-D100/746, left) in dorsal (A), lateral (B), and ventral (C) views. Abbreviations: clpmx, suture for caudolateral process of the premaxilla; dpms, suture for dorsal process of the premaxilla; for, foramen; fs, frontal suture; nm, narial margin; pfs, prefrontal suture; pms, premaxillary suture.

*Nasal* (Fig 4): The nasal is a long and thin element (no more than a millimeter thick), which forms the dorsal wall of the nasal cavity. Rostrally and medially, it forms a very long articulation with the dorsal process of the premaxilla; here it also forms a modest crescentic contribution to the caudal margin of the external naris, as described above. Making up the ventral middle third of the nasal is a grooved surface for reception of the caudolateral process of the premaxilla. The outer surface is generally smooth, marked only by a few neurovascular foramina. There is no indication of a crest or other visual display structure, and there is no circumnarial depression, which is present in *Eotrachodon* and hadrosaurids [29]. Caudally, the nasal twists slightly to articulate with the frontal along a slightly fluted scarf joint, as seen in all hadrosauroids.

**Fig 5:**
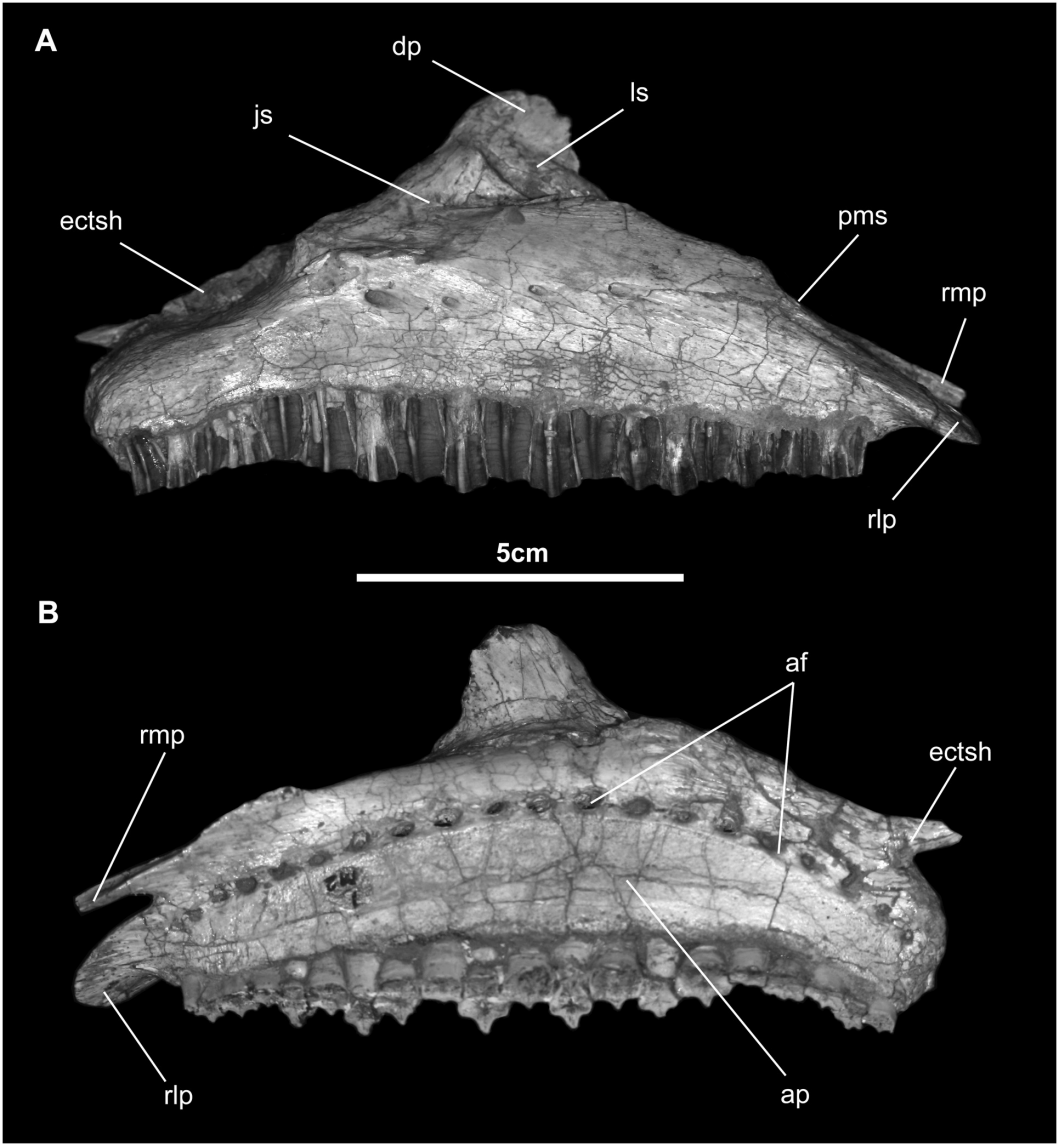
Maxilla of *Gobihadros mongoliensis*. Maxilla (MPC-D100/746, right) in lateral (A) and medial (B) views. Abbreviations: af, alveolar foramina; ap, alveolar parapet; dp, dorsal process; ectsh, ectopterygoid shelf; js, jugal suture; ls, lacrimal suture; pms, premaxillary suture; rlp, rostrolateral process; rmp, rostromedial process.

*Maxilla* (Fig 5): As in *Camptosaurus*, *Probactrosaurus*, *Eotrachodon*, and saurolophine hadrosaurids [7, 26, 46, 51], the maxilla is isosceles-triangular in shape and nearly half as high as long, in lateral view. Rostrally, two processes articulate with the premaxilla: the rostromedial process, which fits into a small, well-defined pit, and the flattened, lobate rostrolateral process, which articulates with a fossa on the undersurface of the premaxillary body. These processes are also seen in numerous species of hadrosauroids, including *Bactrosaurus*, *Altirhinus*, *Shuangmiaosaurus*, *Mantellisaurus*, and saurolophine hadrosaurids [7, 15, 45, 47, 51, 52]. The body of the maxilla diverges markedly from the midline of the skull. The oblique dorsal margin of the bone is marked by a shallow facet for articulation with the caudolateral process of the premaxilla. The apex (dorsal maxillary process), set slightly rostral to the midpoint, is tall and sharply peaked. Contact between the maxilla and lacrimal is along a linear groove directly rostral to the pointed end of the jugal. The articular facet for the jugal dominates the dorsal process. Immediately medial to the front of the jugal facet is a rostrocaudal channel that opens on the premaxillary articular surface and terminates as a curved groove in the middle of the dorsal maxillary process. Beneath the sigmoid ventral margin of the jugal facet, the lateral surface of the maxilla overhangs the tooth row and forms the dorsal extent of the buccal cavity. Four or five neurovascular foramina ranging in diameter from 1 to 2 mm, run rostrocaudally ventral to this buccal shelf; these are also seen in *Protohadros*, *Jinzhousaurus*, *Probactrosaurus*, *Bactrosaurus*, and numerous hadrosauroids [14, 46, 49, 51–53]. The contact with the vomer appears to be along the shoulder where the rostromedial process of the medial maxillary edge lies immediately caudal to the dorsal process. The ectopterygoidal shelf slightly overhangs the lateral wall of the maxilla; continuation of the suture with the ectopterygoid is on the blunt, angular, caudal end of the maxilla. Contact with the pterygoid occurs just caudal to that of the palatine and onto the small process at the caudal extremity of the maxilla. Medially, the maxilla is flat, marked by alveolar foramina and a distinct, slightly arched neurovascular canal that links them. Ventrally, the tooth row is very slightly concave laterally and contains 19 tooth families, or 1.5 families/1.0 cm. The low number of tooth families is consistent with most non-hadrosaurid hadrosauroids, but similar tooth family density is found in *Bactrosaurus*, *Telmatosaurus*, *Probactrosaurus*.

**Fig 6:**
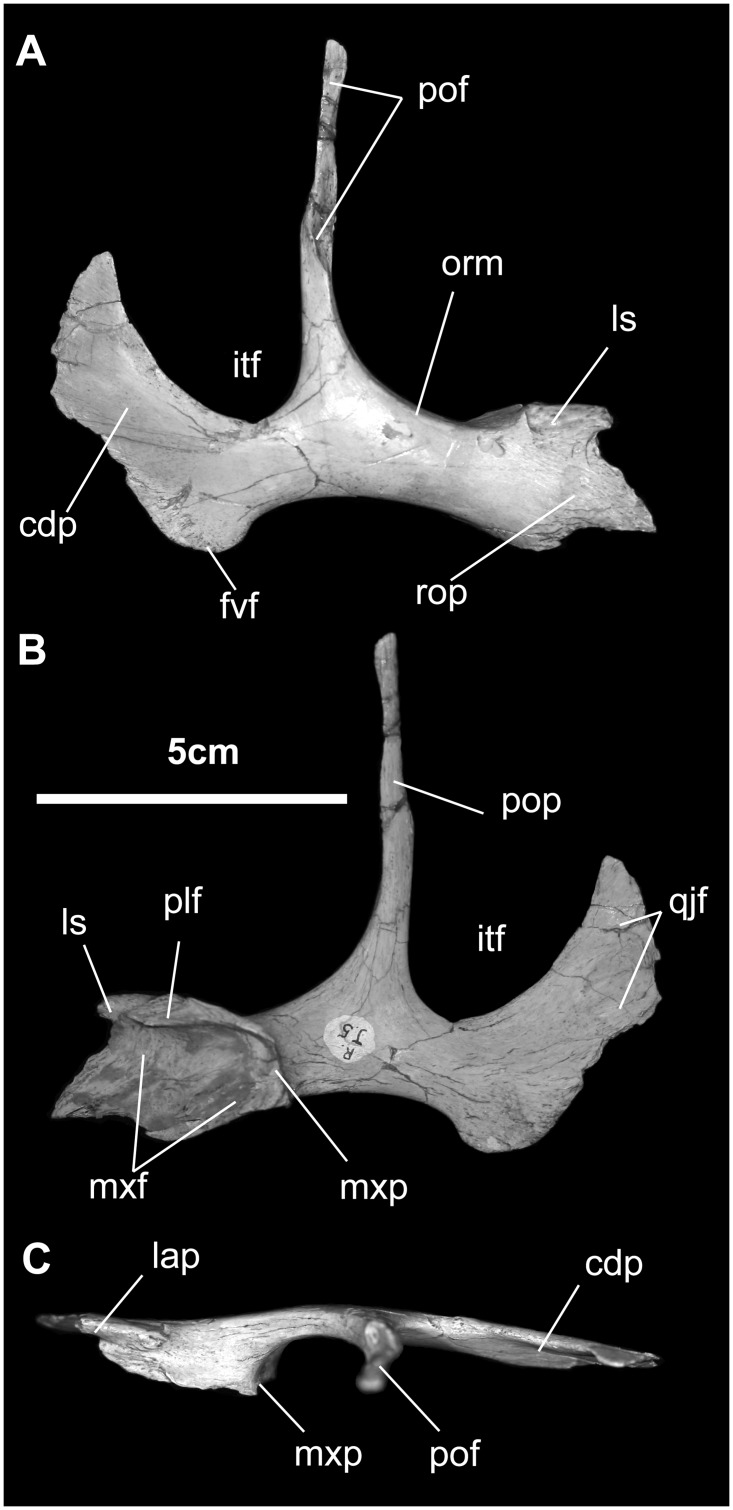
Jugal of *Gobihadros mongoliensis*. Jugal (MPC-D100/746, right) in lateral (A), medial (B), and dorsal (C) views. Abbreviations: cdp, caudal process; fvf, free ventral flange; itf, infratemporal fenestra; lap, lacrimal process; ls, lacrimal suture; mxf, maxillary facet; mxp, maxillary process; orm, orbital margin; plf, palatine facet; pof, postorbital facet; pop, postorbital process; qjf, facet for the rostral process of the quadratojugal; rop, rostral process.

*Jugal* (Fig 6): As in hadrosauroids in general, the jugal of *Gobihadros* is roughly triradiate, consisting of a rostral process, where it articulates with the maxilla, a postorbital process, where it contacts the postorbital, and an expanded caudal process or blade, which articulates with the quadratojugal and the quadrate. The rostral process is slightly dorsoventrally flared and pointed where it contacts the maxilla. The dorsal margin of the rostral process has pushed the lacrimal dorsally to lie completely above the level of the maxilla. Here the articular facet for the jugal process of the lacrimal is divided into two by a gap or foramen between the pillar-like caudal process of the lacrimal that fits into a slot on the jugal and the thinner, linear jugal-lacrimal contact. This foramen may be the opening for the antorbital sinus. Medially, a very small facet directly caudoventral to the rim of the maxillary articulation marks the contact with the ectopterygoid, as also seen in *Iguanodon*, *Mantellisaurus*, *Altirhinus*, *Eolambia*, *Protohadros*, *Probactrosaurus*, and *Bactrosaurus*, but not in hadrosaurids [7, 10, 44–47, 49, 51, 52]. Contact with the palatine is along the dorsalmost portion of the rim of the maxillary suture.

The orbital margin of the jugal is smoothly curved. The postorbital process extends vertically 68 mm above the lowest point of the infratemporal fenestra. Most of this length forms the contact surface for the jugal process of the postorbital; at its terminus, it reaches to the body of the postorbital, making this a relative long postorbital process compared to most hadrosauroids if not all ornithopods. The jugal makes up the entire ventral infratemporal margin. It is also dorsoventrally constricted beneath this fenestra to set off the caudal jugal process, thereby giving this edge of the jugal its sigmoidal silhouette. The depth-to-length ratio of the caudal process is relatively small (0.70–0.90), compared to that of *Jinzhousaurus* and *Altirhinus* [14, 45], where the ratio ranges upward of 1.3. Striations on the lateral aspect of the ventral jugal margin may indicate the presence of soft tissue covering the buccal region.

**Fig 7:**
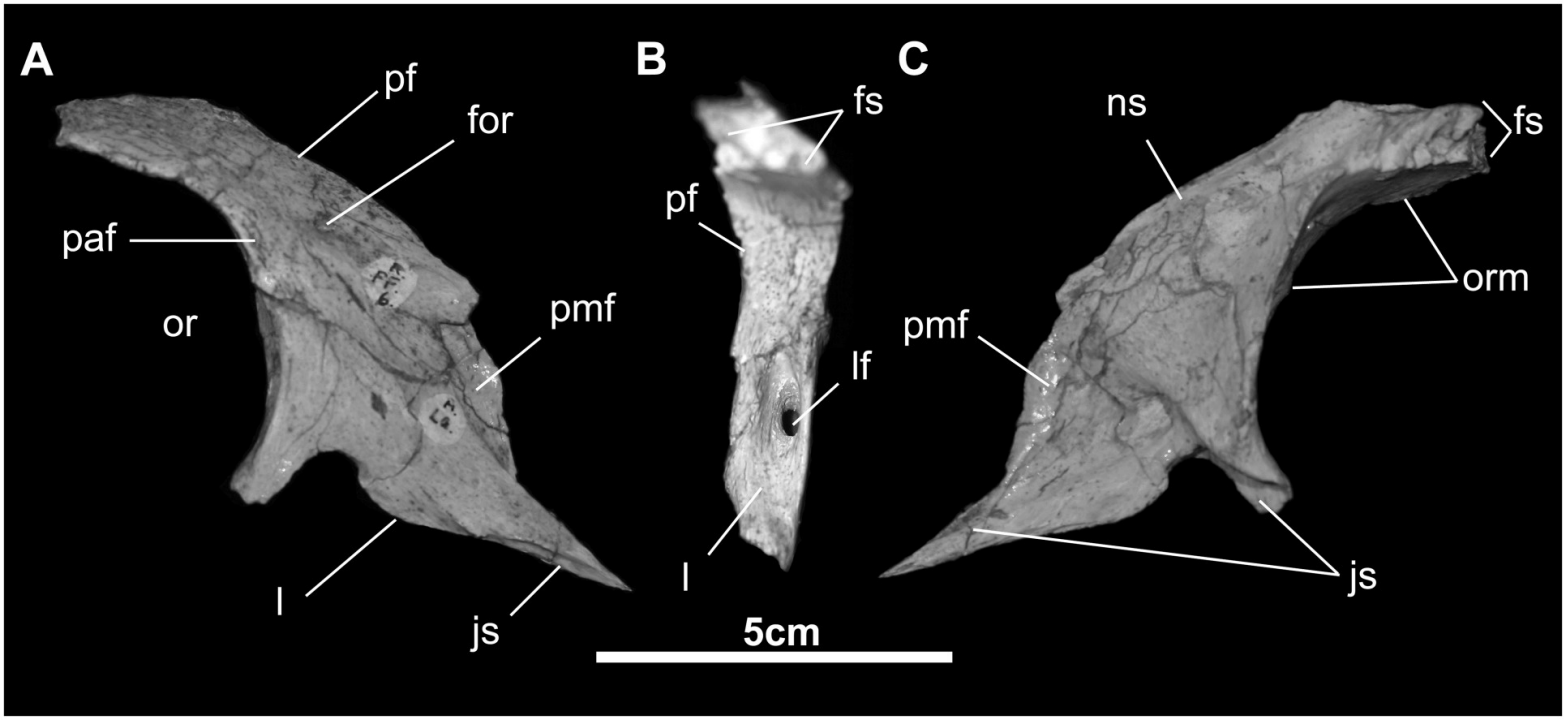
Prefrontal and lacrimal of *Gobihadros mongoliensis*. Prefrontal and lacrimal (MPC-D100/746, right) in lateral (A), posterior (B), and medial (C) views. Abbreviations: for, foramen; fs, frontal suture; js, jugal suture; l, lacrimal; lf, lacrimal foramen; ns, nasal suture; or, orbit; orm, orbital margin; paf, palpebral facet; pf, prefrontal; pmf, facet for caudolateral process of premaxilla.

*Lacrimal* (Fig 7): The lacrimal is plate-like and triangular in lateral view. Along its base, it articulates via a ridge and groove suture with the maxilla. At the caudal extreme of this edge, the lacrimal forms the concave margin of a small fenestra, which is caudally limited by the jugal process of the lacrimal. The rostral edge is marked by a shallow scarf suture for the caudolateral premaxillary process. The base of the lacrimal makes a linear contact with the lateral maxillary wall directly in front of the dorsal process. The dorsal aspect of the lacrimal slips beneath the ventral edge of the prefrontal. The caudal edge of the lacrimal forms the rostral orbital margin. Here the element is thickest where it forms the entrance of the nasolacrimal canal, which exits on the medial aspect of the lacrimal, immediately rostral to the jugal process.

*Prefrontal* (Fig 7): The majority of the crescentic prefrontal is smoothly convex externally, but flares dorsolaterally to form the thin, everted, and wing-like rostrodorsal margin of the orbit. Ventrally it overlaps the dorsal margin of the lacrimal. There is a small facet on the external surface at the base of the prefrontal that marks the articulation of the terminus of the caudolateral process of the premaxilla. Immediately above this facet are one or two neurovascular foramina. The internal surface of the prefrontal is markedly concave except where it contacts the frontal. This articulation is via a complex, tongue-and-groove joint on the caudomedial surface of the prefrontal.

**Fig 8:**
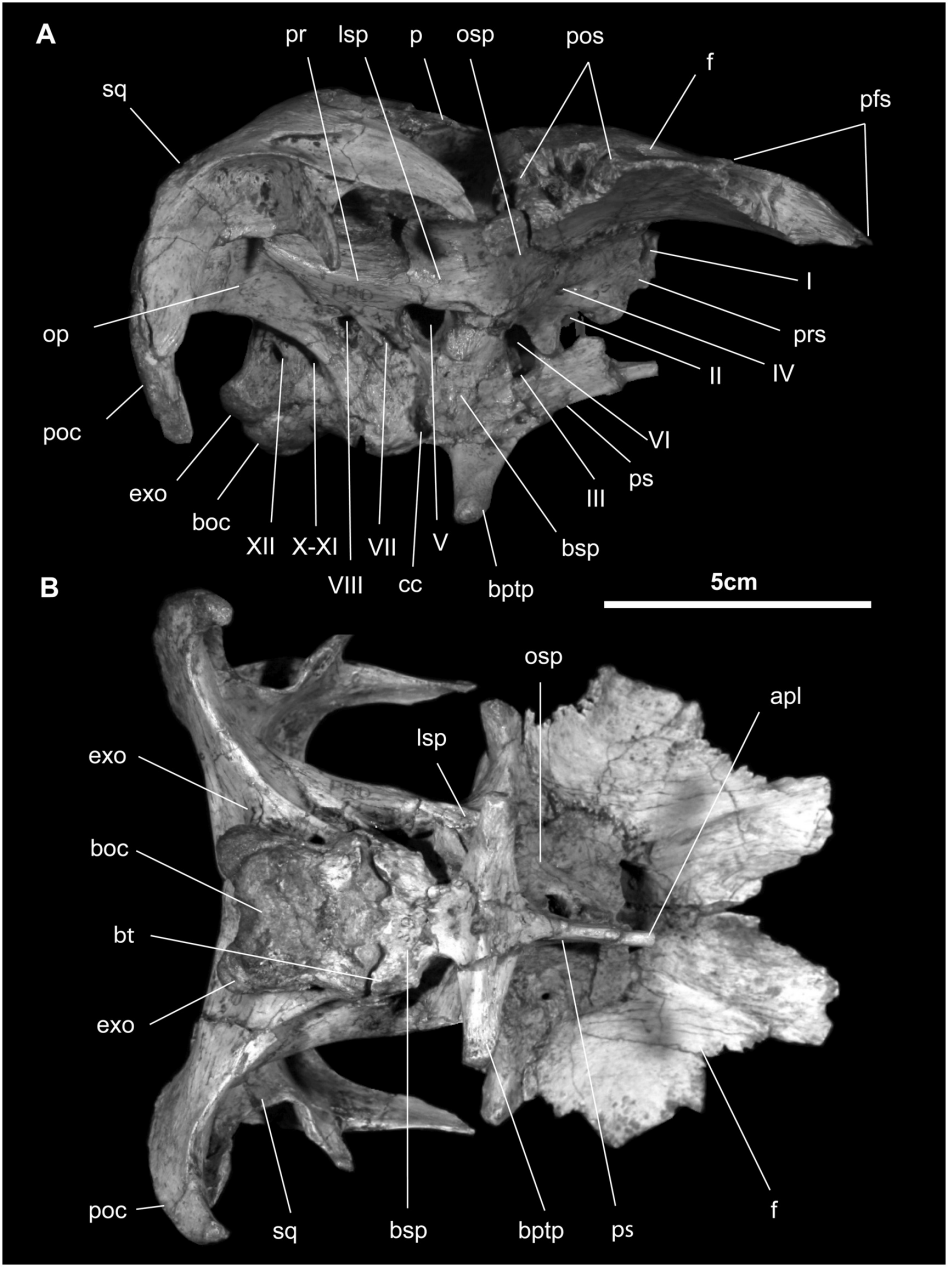
Braincase of *Gobihadros mongoliensis*. Braincase (MPC-D100/746) in right lateral (A) and ventral (B) views. Abbreviations: apl, arteria palatinus; boc, basioccipital; bpt, basipterygoid; bptp, basipterygoid process; bsp, basisphenoid; bt, basal tubera; cc, carotid canal; exo, exoccipital; f, frontal; lsp, laterosphenoid; op, opisthotic; osp, orbitosphenoid; p, parietal; pfs, prefrontal suture; pos, postorbital suture; pr, prootic; prs, presphenoid; ps, parasphenoid; sq, squamosal; I-XII, foramina for cranial nerves.

*Frontal* (Fig 8): In dorsal view, the flat, subtriangular frontal is two-thirds as wide as long. Rostrally, it bears a broad, grooved, and shallow articular facet for the nasal and immediately lateral a complex excavated pit for the prefrontal. The orbital margin is short and grooved on its ventral surface. An upward doming over a braincase is absent. The interfrontal joint is interdigitated along its length. Caudally, the paired frontals part to accommodate a rostral process of the parietal. The caudolateral corner of the frontal is complexly excavated for the postorbital as well as for the head of the laterosphenoid. The caudal surface of the frontal receives the rostral face of the parietal and the laterosphenoid.

Ventrally, the most prominent feature is the endocranial surface for the olfactory bulbs and tracts on the rostral half of the frontal. The ventral surface of the orbital margin is perforated by a few small neurovascular foramina. On its caudal half, just behind and forming the lateral wall of the olfactory tract, the ventral surface of the frontal articulates with the orbitosphenoid and, more laterally, with the body of the laterosphenoid.

*Parietal* (Fig 8): The parietal is stout and hour-glass in shape in dorsal view, possessing only a modest, straight to slightly down-warped sagittal crest that is more than half the length of the supratemporal fenestrae. Rostrally an interfrontal process slips between the paired frontals. Two ridges diverge from the sagittal crest to set off the rear of the interfrontal process. Contact with the frontal is extensive, covering nearly its entire caudal surface. A small portion of the parietal lateral to this frontal suture contacts the postorbital. The squamosal rides over the rear margin of the parietal. Ventrally, the parietal articulates in series with the laterosphenoid rostrally, with the prootic intermediately, and with the opisthotic/exoccipital complex caudally. Midway along this suture, the parietal forms the dorsal margin of the foramen for the median cerebral vein.

**Fig 9:**
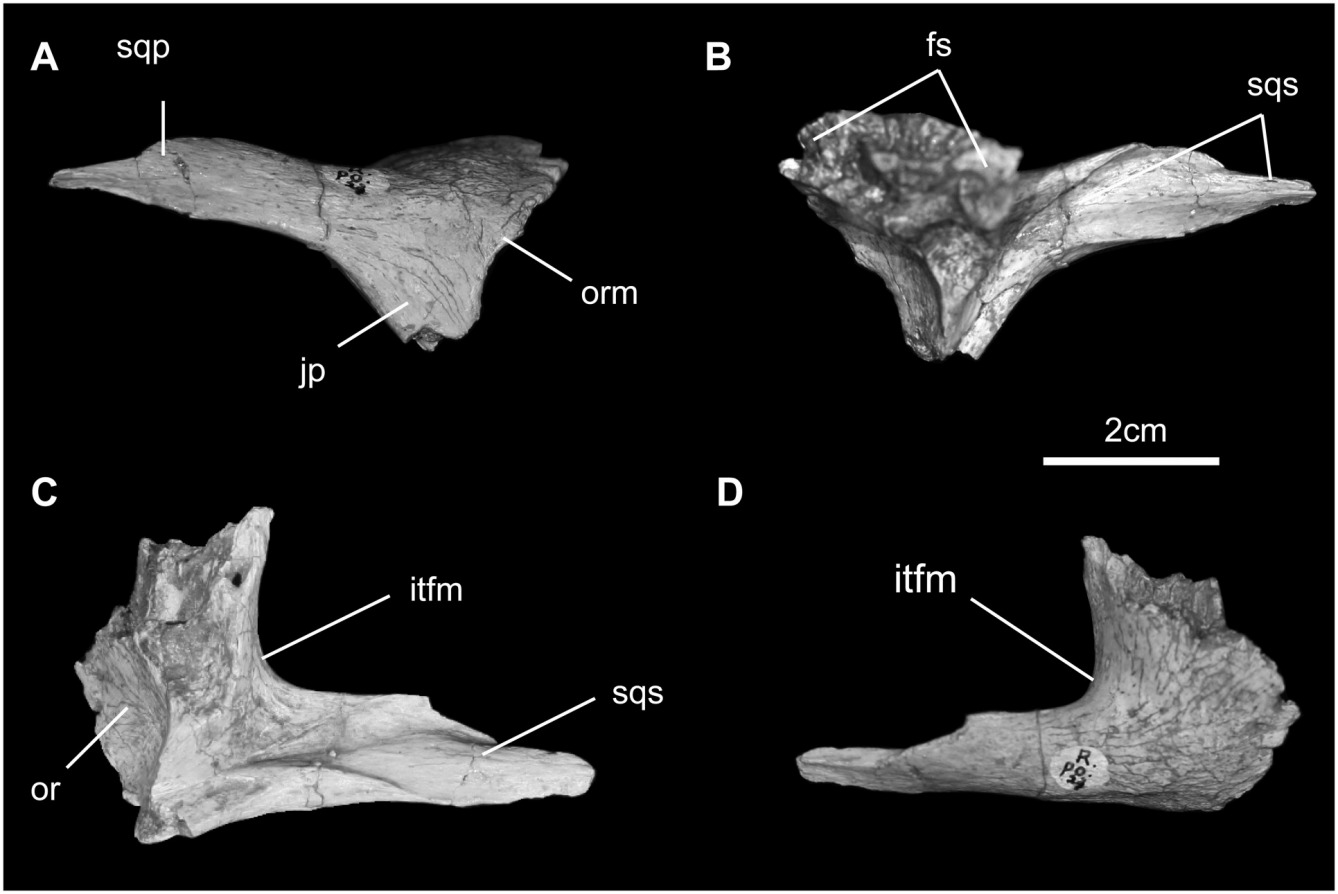
Postorbital of *Gobihadros mongoliensis*. Postorbital (MPC-D100/746, right) in lateral (A), medial (B), ventral (C), and dorsal (D) views. Abbreviations: fs, frontal suture; itfm, infratemporal fenestra margin; jp, jugal process; or, orbit; orm, orbital margin; sqp, squamosal process; sqs, squamosal suture.

*Postorbital* (Fig 9): The postorbital is a triradiate element with medial, caudal, and ventral processes. It closely resembles the postorbital of *Bactrosaurus johnsoni* [52]. Centrally it forms the thickened caudodorsal border on orbital rim. Medially, its contact with the frontal is made up a complex tongue-and-groove articulation. Ventral to the postorbital-frontal suture is a pit that accommodates the head of the laterosphenoid. The caudal process overlaps the squamosal for most of the supratemporal bar; here it is a simple non-digitate suture. The caudal end of the process is not bifurcated, as is seen frequently in hadrosaurids. Finally, contact with the jugal is by way of a long, thin scarf joint on the caudomedial surface of the ventral (postorbital) process.

*Squamosal* (Fig 8): The squamosal is roughly triradiate in form. Medially, it forms the caudal margin of the supratemporal fenestra, continuing on toward the midline and its counterpart, separated here by a narrow band of the parietal, as in *Jinzhousaurus*, *Jintasaurus*, *Probactrosaurus*, *Bactrosaurus*, *Levnesovia*, and *Tanius* [14, 18, 19, 46, 54]. The cranial process forms much of the upper temporal arcade; here it is marked externally by the single angular articulation for the caudal process of the postorbital. The ventral process descends behind the quadrate cotylus to form a deep, nearly vertical, and rugose postquadratic process. The caudal surface of this process forms a butt joint with the base of the paroccipital process and also contacts the lateral margin of the supraoccipital in caudal view. Externally, the deep cotylus for the head of the quadrate dominates the squamosal in external view. Immediately rostral to the cotylus is the prequadratic process; sandwiched between it and the articular surface for the postorbital is the small, triangular scar for *m*. *adductor mandibulae externus superficialis* [55].

**Fig 10:**
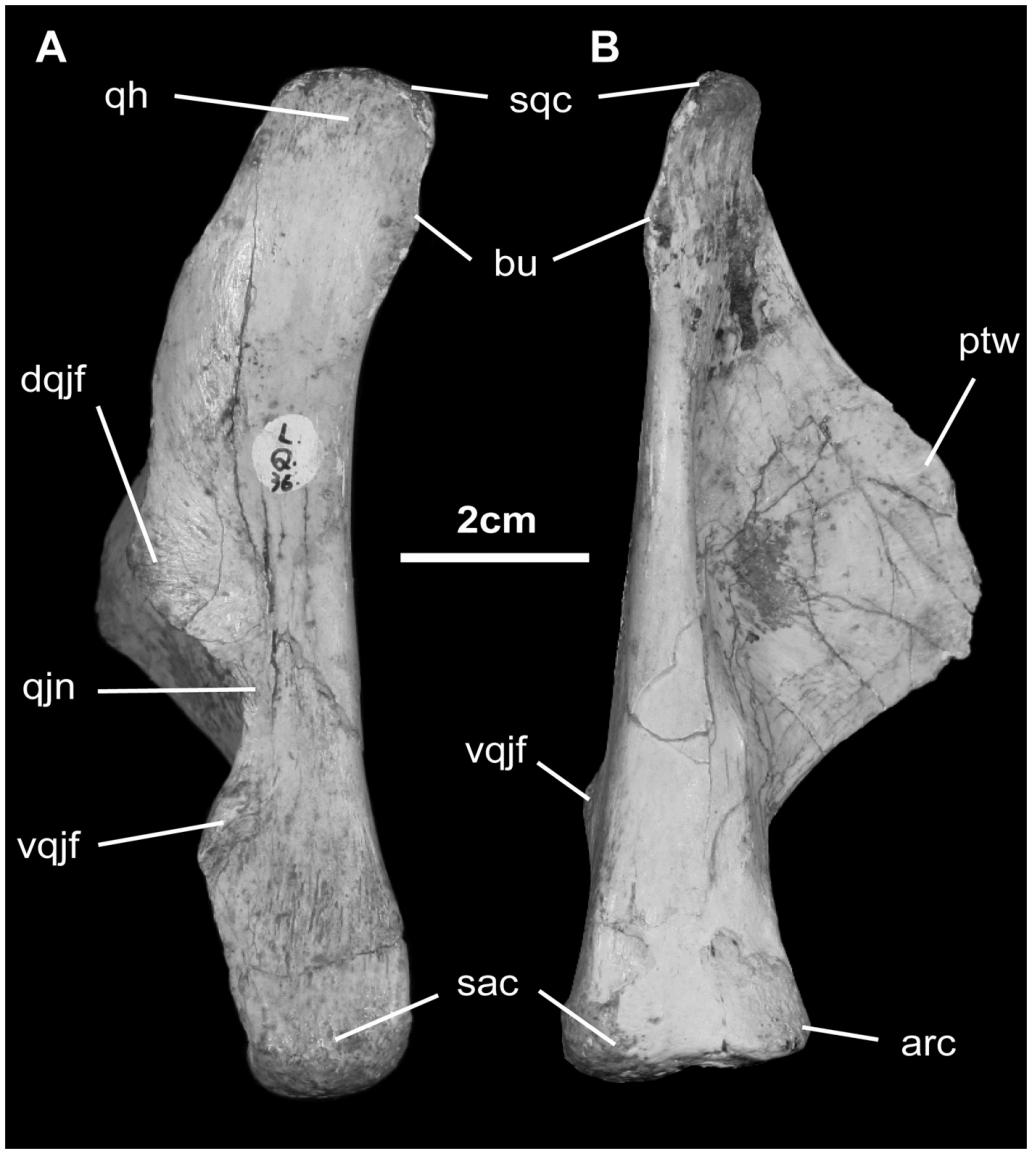
Quadrate of *Gobihadros mongoliensis*. Quadrate (MPC-D100/746, left) in lateral (A) and posterior (B) views. Abbreviations: arc, articular condyle; bu, buttress beneath head of quadrate; dqjf, dorsal quadratojugal facet; ptw, pterygoid wing; qh, quadrate head; qjn, quadratojugal notch; sac, surangular condyle; vqjf, ventral quadratojugal facet.

*Quadrate* (Fig 10): The quadrate in lateral view is moderately robust. The dorsal head is nearly triangular in dorsal view, and it is very shallow, as in *Mantellisaurus*, *Jeyawati*, *Bactrosaurus*, and *Telmatosaurus*. Immediately beneath the head, the rostral surface is beveled and strongly striated for sutural ligaments that bind this region with the prequadratic process of the squamosal. The caudal margin is buttressed where it contacts the postquadratic process of the squamosal. This squamosal articulation positions the quadrate in a canted position, giving the skull a triangular outline in occipital view (Fig 2D). In lateral view, the caudal aspect of the quadrate shaft is modestly concave. Beneath midshaft, the lateral aspect of the quadrate shaft is dominated by the articular surface for the quadratojugal. There is a modest gap here, indicating that the paraquadratic opening was reasonably well defined. The lower margin of the quadrate here is slightly buttressed. The ventral end of the quadrate is only slightly expanded and obviously bicondylar. The lower and larger lateral condyle articulates with the surangular glenoid, while the slightly higher, smaller medial condyle articulates with the articular glenoid. The lateral condyle is not large and globular as in hadrosaurids [51], but is similar in relative size and morphology to *Bactrosaurus*, *Gilmoreosaurus*, and *Jeyawati* [56–58]. Together, the ventral quadrate gives a slight medial elevation to the axis of mandibular rotation. Medially, the thin, platelike pterygoid wing extends rostromedially at approximately 45° from the lateral quadrate wall. The lower margin of the ala is thickened; the central region is markedly convex laterally, forming the reciprocal surface for the quadrate ala of the pterygoid.

**Fig 11:**
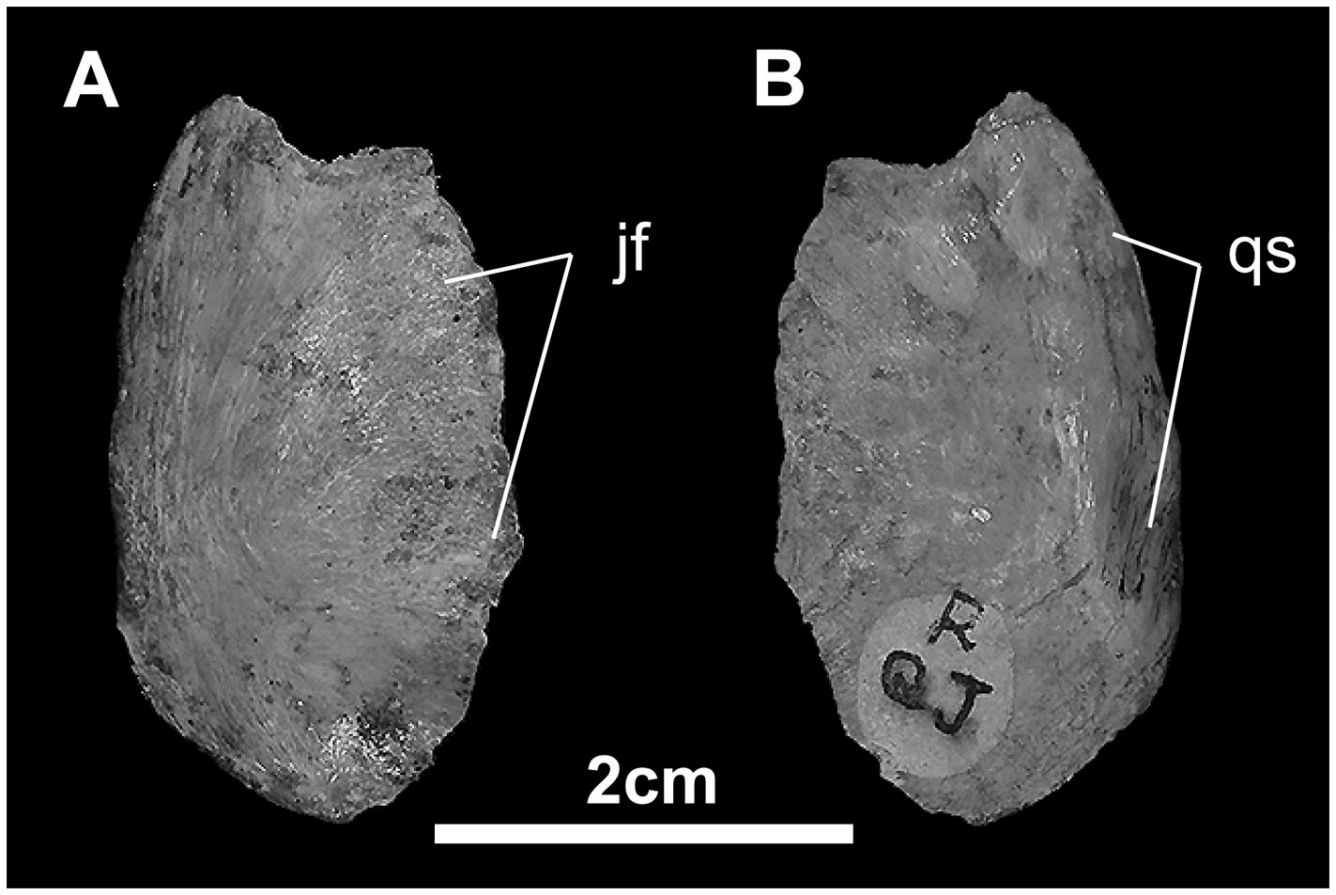
Quadratojugal of *Gobihadros mongoliensis*. Quadratojugal (MPC-D100/746, right) in medial (A) and lateral (B) views. Abbreviations: jf, jugal facet; qs, quadrate suture.

*Quadratojugal* (Fig 11): The quadratojugal is a thin, oblong element located between the jugal and quadrate. Both external and internal surfaces are beveled rostrally and caudally respectively from a median thickened body. Externally, this beveled region is covered by the jugal, while internally it marks the regions that contact the quadrate.

*Supraoccipital* (Fig 8): The supraoccipital is a relatively large, subtriangular element that makes up the dorsal region of the occiput. Its caudal surface is inclined steeply forward at approximately 45°, unlike in *Probactrosaurus*, *Ouranosaurus*, *Dakotadon*, *Lurdusaurus*, *Eolambia*, *Iguanodon*, and *Mantellisaurus*, where the caudal supraoccipital surface is nearly vertical [10, 44, 46–48, 50, 59]. The ascending process contacts the parietal dorsally and laterally. There is a prominent median nuchal crest. On each side of this crest is a low boss separated by a furrow; this boss forms the area of insertion of *m*. *rectus capitis caudalis* [60]. In addition to contacting the caudolateral margin of parietal rostrally, the lateral supraoccipital process forms a prominent horizontal ridge with the opisthotic/exoccipital complex immediately above where the latter forms the dorsal margin of the foramen magnum. The ventrolateral corners of the supraoccipital are inset under the squamosals such that the supraoccipital is “locked” between them. The post-temporal foramen is found at the junction of the squamosal, opisthotic/exoccipital complex, and supraoccipital. The ventral margin of the supraoccipital forms a prominent horizontal ridge where it contacts the exoccipital/opisthotic complex.

*Exoccipital/Opisthotic Complex* (Fig 8): The ventral margin of the fused exoccipital-opisthotic complex contacts the basioccipital and basisphenoid. The exoccipital contributes a small occipital condylid and directly above forms the lateral and dorsal margins of the oval foramen magnum. The dorsal border of the exoccipital-opisthotic complex meets the supraoccipital along a prominent horizontal ridge. Immediately above this ridge, the ventromedial margins of the post-temporal foramen are formed by the exoccipital/opisthotic complex. Laterally, the caudal aspect of the complex is excavated to form the attachment site of *m*. *obliquus capitis magnus* [60]. Beneath the ridge at the supraoccipital suture is the *m*. *rectus capitis caudalis* scar. The outer margin of the exoccipital-opisthotic complex arches dorsolaterally to form the large caudolaterally-projecting paroccipital process. At their extremities, the paroccipital processes are curved rostrally.

In lateral view, the ventral edge of the paroccipital process forms a sharp rostroventral crest (*crista tuberalis*) that splits rostroventrally into two ridges. One, the more caudal and rounder of the two, merges with the dorsal margin of the exoccipital condylid, while the other, sharper ridge, continuing toward the suture between the basioccipital and basisphenoid, marks the suture between the exoccipital/opisthotic complex and the prootic. The concave ventrolateral aspect of the exoccipital-opisthotic complex is pierced by three foramina. Two, just above the suture for the basioccipital transmitted the hypogossal nerve (c.n. XII). The exit for the spinal accessory and vagus nerves (c.nn. X and XI) is found immediately behind *crista tuberalis*. The auditory foramen opens in front of *crista tuberalis* directly above the suture with the basisphenoid. At its base, contact with the basioccipital is somewhat difficult to discern given its nearly fused state, but it appears to correspond to a line extending from the base of the occipital condylids to the base of the basal tubera.

*Prootic* (Fig 8): With the laterosphenoid and exoccipital/opisthotic, the prootic forms the lateral wall of the braincase. It forms a butt joint with the laterosphenoid immediately above the foramen for the trigeminal nerve (c.n. V). From this foramen, the largest of all of the cranial nerve foramina, a groove accommodating the ophthalmic branch of the trigeminal nerve (V_1_) follows the base of the laterosphenoid. A canal extending ventrally from the trigeminal foramen marks the pathway of the combined maxillary and mandibular divisions (V_2_, V_3_). The surface of the prootic between the first and second (with the third) divisions marks the scar for *m*. *constrictor ventralis*. Directly caudal to the groove for the second and third divisions is a caudolateral flange that shrouds the pathway of the hyomandibular branch of the facial nerve (c.n. VII). At the top of this groove is the foramen of the facial nerve. Above and continuing caudodorsally, a roughened eminence traverses the lateral surface of the exoccipital-opisthotic complex known as *crista prootica* (the scar for *m*. *constrictor dorsalis*). This eminence marks an elongate scarf articulation between the prootic and opisthotic and also a continuation of *crista prootica*. Below the contact between the prootic and opisthotic is a small, deep depression marking the caudalmost extent of the otic vestibule. Dorsal to the prootic articulation and *crista prootica*, the rostrodorsal surface of the base of the paroccipital process is excavated for contact with the supraoccipital.

*Laterosphenoid* (Fig 8): The head of the laterosphenoid forms what appears to be a synovial joint with the underside of the postorbital and frontal. The frontal articulation continues transversely across the orbital surface to meet its suture with the orbitosphenoid. A butt joint is found between the laterosphenoid and prootic, and between the laterosphenoid and parietal.

*Orbitosphenoid* (Fig 8): The paired orbitosphenoids are found in articulation with the ventral surface of the frontals, as well as the laterosphenoids, basisphenoid, and each other. C.n. I emerges rostrally where the right orbitosphenoid articulates with the left. Contact with each frontal and laterosphenoid is via a laterally convex, digitate suture. Foramina for cranial nerves II, III, IV, and VI are found in the central and caudal aspects of the orbitosphenoid.

*Basisphenoid* (Fig 8): In ventral view, the basisphenoid is triangular and appears like a thin arrowhead. The handle of the blade, the basal tubera, is twice as wide as long. These tubera are rugose probably for attachment of *m*. *rectus capitis ventralis*. A sinuous groove across the tubera marks the suture with the basioccipital. More rostrally, the basipterygoid process forms lateral to the midline furrow and rostral to the entrance of the carotid canal. The basipterygoid process is relatively long (23 mm from the midline), and slightly ventrolaterally directed approximately 80° from the sagittal plane (the basipterygoid process appears to be deformed by dorsoventral compression). The basipterygoid articular facets are directed rostroventrally. A midline prong, immediately between the basipterygoid processes, is found in *Gobihadros*, *Camptosaurus*, *Ouranosaurus*, *Bactrosaurus*, and *Levnesovia* [18, 48, 52, 61]. Entrance to the carotid canal is located between the flange formed by the base of the basipterygoid process and basal tubera. This canal travels rostrally and probably slightly medially through the basisphenoid to exit through the caudoventral wall of the hypophyseal fossa. A vertical groove directly above the entrance of the canal corresponds to path of palatine ramus of facial nerve; the latter also enters the carotid canal. A style-like rostral projection from the body of the basisphenoid may be the separate center of ossification known as the parasphenoid. A foramen for the median palatine artery is found where the basisphenoid meets the orbitosphenoid.

*Basioccipital* (Fig 8). The basioccipital is formed principally by the occipital condyle, the intermediate collum, and the caudal half of the basal tubera. The occipital condyle is reniform as it wraps around, and forms 9 mm of, the ventral margin of the foramen magnum. Laterally it butts the condylids of the exoccipitals. The collum is short (10 mm) and equal in width of the occipital condyle. Farther forward, the basioccipital portion of the basal tubera projects rostrally, slightly laterally, and slightly ventrally to contact the basisphenoid portion of the basal tubera. The former is divided by a shallow midline sulcus and separated by a prominent transverse groove from the latter. There is no axial ridge between basal tubera, which is seen in *Camptosaurus*, *Uteodon*, *Cumnoria*, *Dakotadon*, and *Jintasaurus* [12, 19, 50, 61–64].

**Fig 12:**
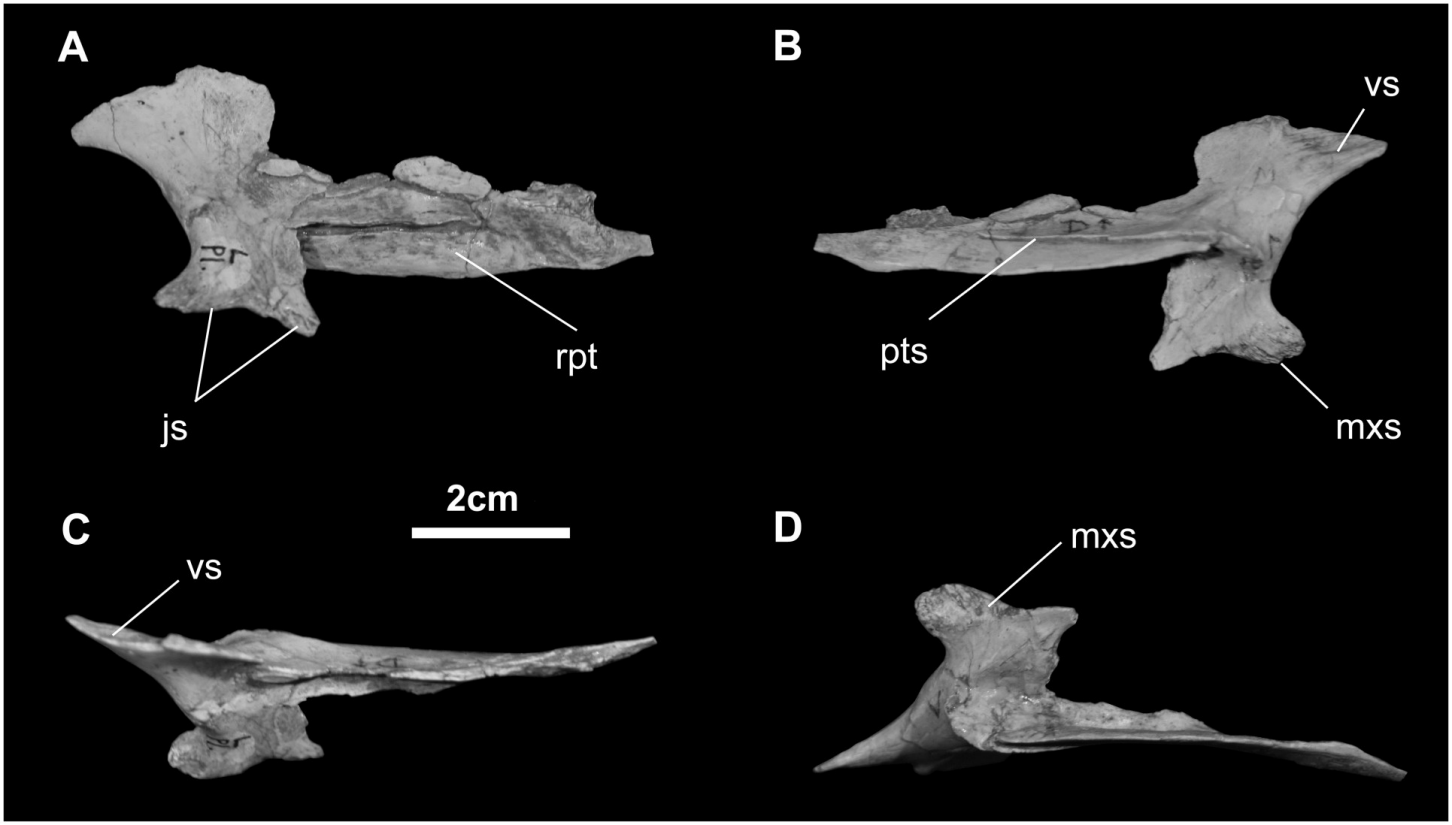
Palatine of *Gobihadros mongoliensis*. Palatine (MPC-D100/746, left) in lateral (A), medial (B), dorsal (C), and ventral (D) views. Abbreviations: js, jugal suture; mxs, maxillary suture; pts, pterygoid suture; rpt, rostral pterygoid; vs, vomer suture.

*Palatine* (Fig 12): The base of the palatine makes an inclined suture with the dorsomedial edge of the caudal process of the maxilla. A stout process located toward the front of this palatine–maxilla contact extends laterally to contact the jugal. The palatine also articulates with the pterygoid along its oblique dorsolateral margin.

**Fig 13:**
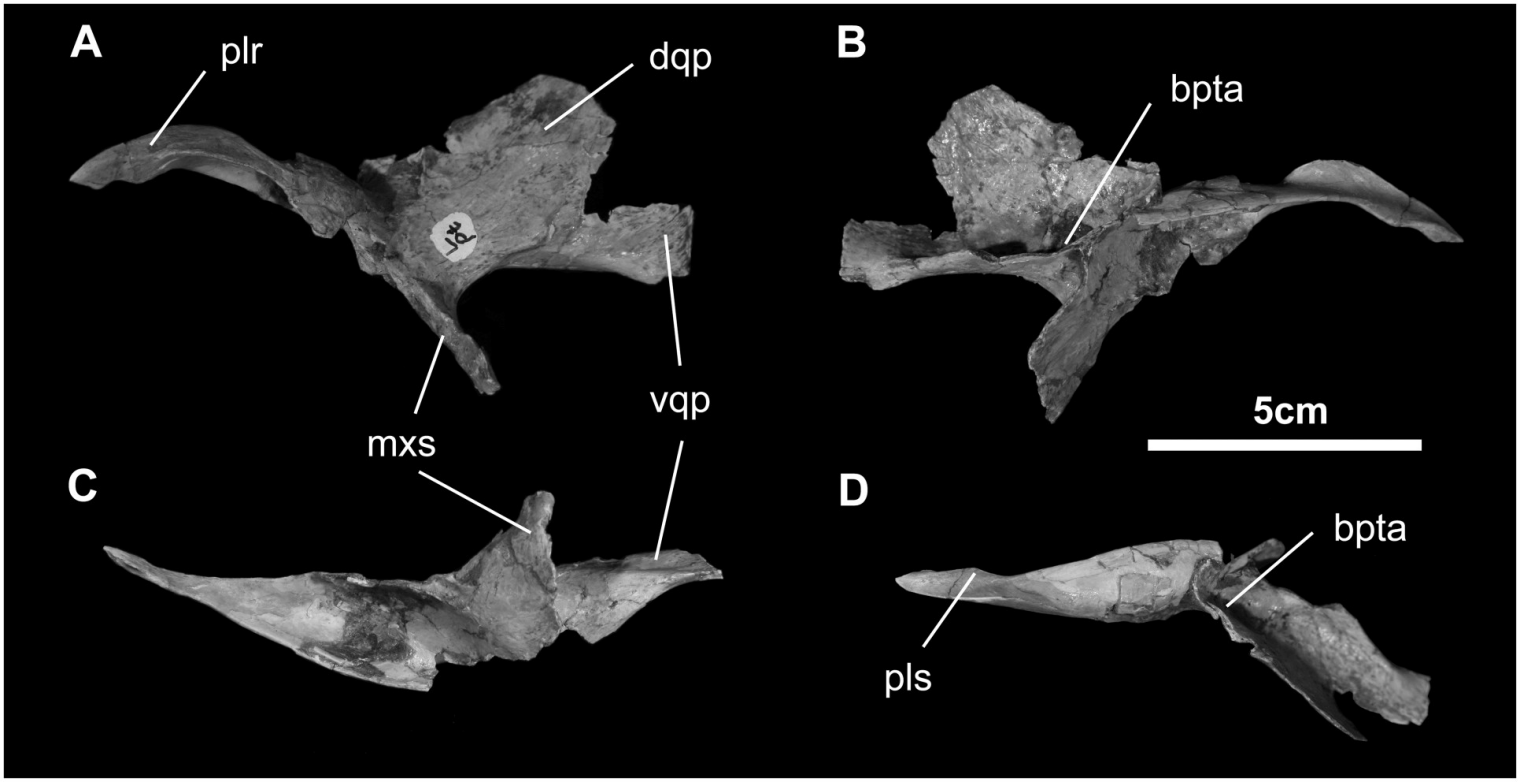
Pterygoid of *Gobihadros mongoliensis*. Pterygoid (MPC-D100/746, left) in lateral (A), medial (B), ventral (C), and dorsal (D) views. Abbreviations: bpta, basipterygoid articulation; dqp, dorsal quadrate process; mxs, maxillary suture; plr, palatine ramus; pls, palatine suture; vqp, ventral quadrate process.

*Pterygoid* (Fig 13): As in other ornithopods, the pterygoid of *Go*. *mongoliensis* is a tetraradiate element. The palatine ramus is incomplete, yet appears to extend dorsally and laterally where it contacts the palatine. Ventrally, the pterygoid sends a short process to contact the ectopterygoid and the rear end of the maxilla; this process is somewhat caudally deformed. Two alar projections on the caudal end of the pterygoid articulate with the quadrate. The larger upper ala fits against the pterygoid ala of the quadrate, while the thickened lower ala reinforces this contact with the quadrate. Additional buttressing is found between the ectopterygoid and upper quadrate rami. Halfway along this buttress, on the medial aspect of the pterygoid, is a well-defined saddle for the synovial articulation with the basipterygoid process of the basisphenoid.

#### Lower jaw

**Fig 14:**
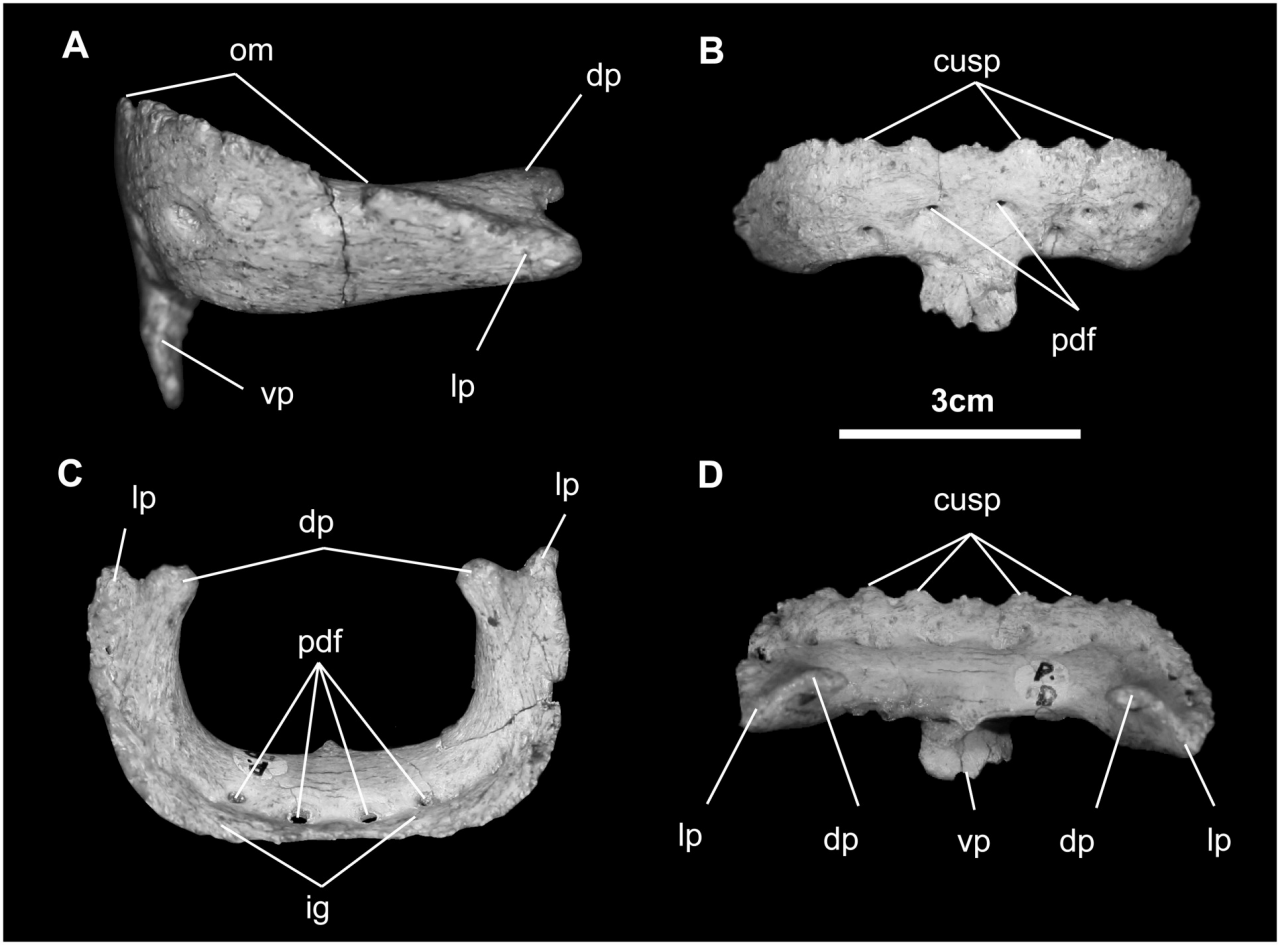
Predentary of *Gobihadros mongoliensis*. Predentary (MPC-D100/746) in lateral (A), anterior (B), dorsal (C), and posterior (D) views. Abbreviations: cusp, denticulations on oral margin; dp, dorsal process; ig, internal groove; lp, lateral process; om, oral margin; pdf, predentary foramina, vp, ventral process.

*Predentary* (Fig 14): In dorsal view, the predentary is transversely straight rostrally, but otherwise U-shaped, with nearly parallel lateral processes, much like *Ouranosaurus*, possibly *Dakotadon*, and virtually all hadrosaurids [48, 50, 51], unlike the less squared-off, more horseshoe shape seen in *Mantellisaurus*, *Probactrosaurus*, *Bactrosaurus*, *Protohadros*, and *Saurolophus* [7, 46, 47, 49, 51, 52]. The rostral surface is inclined approximately 30° to the horizontal. The oral margin of the predentary bears seven large, triangular denticles centered at the midline, and as many as 11 much smaller denticles extending to the end of the lateral processes; these are similar to, but much more abundant than, seen in *Probactrosaurus* and *Altirhinus* [45, 46]. These denticles strongly suggest the presence of a keratinous rhamphotheca. Together, this rhamphotheca and that of the premaxilla (see above) intermesh when the mouth is closed. More internally are 12 neurovascular foramina that occupy a prominent groove, piercing to the external predentary surface. The lateral processes enclose the front of the dentaries along a bilobed overlapping suture. The ventral process, where the predentary clasps the under surface of the mandibular symphysis, may be divided along the midline into two flat lobes. There is a small dorsal process that overrides the dorsal margin of the dentary symphysis, as in *Iguanodon*, *Mantellisaurus*, *Levnesovia*, *Ouranosaurus*, *Probactrosaurus*, *Protohadros*, *Eolambia*, and *Bactrosaurus* [10, 18, 44, 46–48, 52].

**Fig 15:**
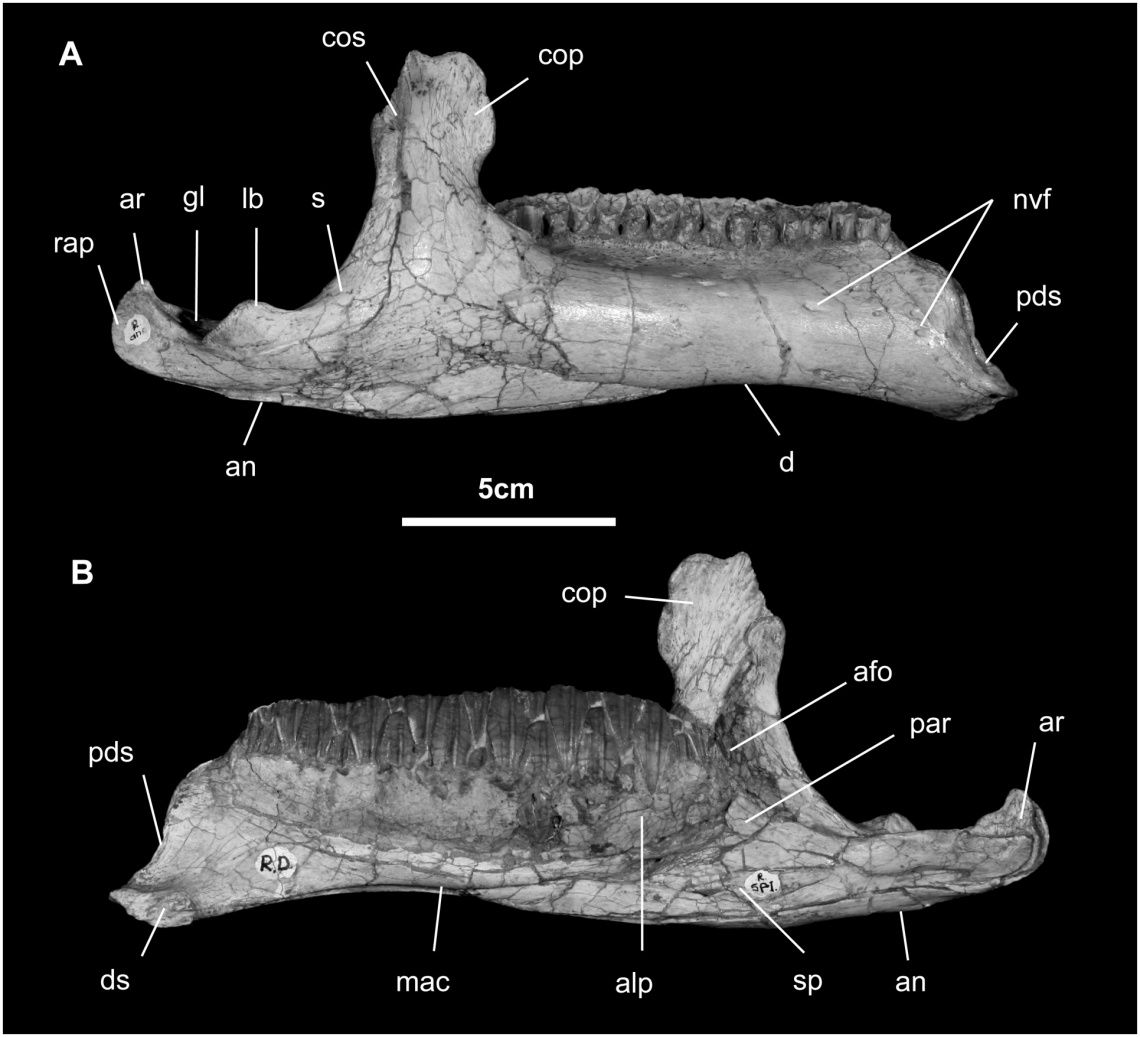
Mandible of *Gobihadros mongoliensis*. Mandible (MPC-D100/746, right) in lateral (A) and medial (B) views. Abbreviations: afo, adductor fossa; alp, alveolar parapet; an, angular; ar, articular; cop, coronoid process; cos, coronoid portion of surangular; ds, dentary symphysis; gl, glenoid; lb, lateral buttress; mac, mandibular canal; nvf, neurovascular foramen; par, prearticular; pds, predentary suture; rap, retroarticular process; s, surangular; sp, splenial.

*Dentary* (Fig 15): The dentary, containing 18 tooth positions, is 157 mm long; the height of the tooth-bearing portion of the ramus is 37 mm. Rostrally, the dentary symphysis is small and oriented approximately horizontal; it is rough and irregular, testifying to the presence of strong symphyseal ligaments. Contact with the predentary is marked by a distinct sigmoidal groove on the external surface of the dentary. Several large neurovascular foramina are associated with this articulation area for the predentary. The orientation of the dentary rostral to the tooth row is moderately down-turned, but not as extreme as seen in *Ouranosaurus* and *Protohadros* [48, 49], and there is no significant diastema between the oral margin of the predentary and the first dentary tooth, much like *Altirhinus*, *Xuwulong*, *Jinzhousaurus*, *Jeyawati*, *Equijubus*, and *Eotrachodon*, among several other non-hadrosaurid hadrosauroids [14, 15, 22, 29, 45, 56, 65]. The dorsal and ventral margins of the dentary are straight and parallel. Buccal emargination is substantial and the dentary bears six to eight foramina that likely contained buccal neurovasculature. The dentary tooth row is straight to slightly concave medially.

The coronoid process of the dentary is robust, rising at a right angle to the long axis of the dentary body, much like that seen in *Penelopognathus*, *Bactrosaurus*, *Altirhinus*, *Protohadros*, and *Jinzhousaurus*, and other non-hadrosaurid hadrosauroids [14, 45, 49, 52, 65, 66]. It gradually arises from the lateral wall of dentary to another 1.3 times the height of the ramus. In doing so, the coronoid process comes to lie lateral to the more distal teeth. Dorsally it is expanded, pitted, and striated laterally and medially, reflecting the attachment of adductor musculature and articulation for the coronoid bone. The caudal edge of the coronoid process is notched to receive dorsal process of surangular. Ventrally the tooth row is linear in dorsal view.

Medially, the body of dentary is shallowly convex dorsoventrally, marked by the curved line of alveolar foramina and interconnecting groove. The coronoid bone articulates with the caudal margin of the coronoid process of the dentary and continues medially to form the medial margin of the mandibular fossa. Ventrally, the mandibular canal extends nearly the entire length of the dentary, being widest caudally at the adductor fossa and gradually diminishing in width and depth to terminate faintly just caudal to the mandibular symphysis. The splenial covered the mandibular canal in life, but in both mandibles it is displaced ventrally. Articulation with the surangular is along the caudal dentary margin as a near-vertical tongue and groove and, more ventrally, as a sleeve around the rostral part of the surangular.

*Surangular* (Fig 15): The surangular is roughly crescentic in both lateral and dorsal views. Rostrodorsally, a complex tongue-and-groove joint reinforced by transverse thickening is made with the coronoid bone and the coronoid process of the dentary. The surangular contributes substantially to the rear of the coronoid process in lateral view, as in *Mantellisaurus*, *Ouranosaurus*, *Equijubus*, *Probactrosaurus*, *Altirhinus*, and *Telmatosaurus* [7, 15, 45–48, 51, 67], but makes a narrow contribution in all hadrosaurids and possibly *Jinzhousaurus* [14, 51, 65]. There is no evidence of an external mandibular fenestra, as is expected of all ornithopods. Nor is there a surangular foramen, as seen in *Mantellisaurus*, *Ouranosaurus*, *Altirhinus*, *Jinzhousaurus*, *Equijubus*, *Probactrosaurus*, and *Protohadros* [14, 15, 45–49, 65].

The surangular comprises more than half of the jaw joint, where it is shallowly concave and subcircular. The rostral margin of the glenoid is elevated to form a shoulder that extends toward the base of the coronoid process. The glenoid margin is marked laterally by a dorsally directed prominence that is pitted and grooved at its apex. More caudally, that part of the retroarticular process formed by the surangular is elongate, strongly upturned, and highly ridged (scar for *m*. *pterygoideus externus*). The suture for the angular is found along the ventrolateral aspect of the entire length of the surangular. Contact with the articular is limited to the roughened caudal aspect of the medial surface of the surangular. Immediately rostral, the surangular is smoothly biconcave, forming the inlet to the mandibular canal.

*Angular* (Fig 15): The angular is small and dorsoventrally narrow, exposed only in medial view. It articulates with the articular dorsally and the surangular below and laterally.

*Articular* (Fig 15): The articular is small and oblong; it articulates with the caudal and caudomedial surface of the surangular. Laterally it forms the remainder of the jaw joint.

*Splenial* (Fig 15): The splenial covers the mandibular canal of the dentary. Slender along most of its length, it expands caudally as it approaches the angular.

**Fig 16:**
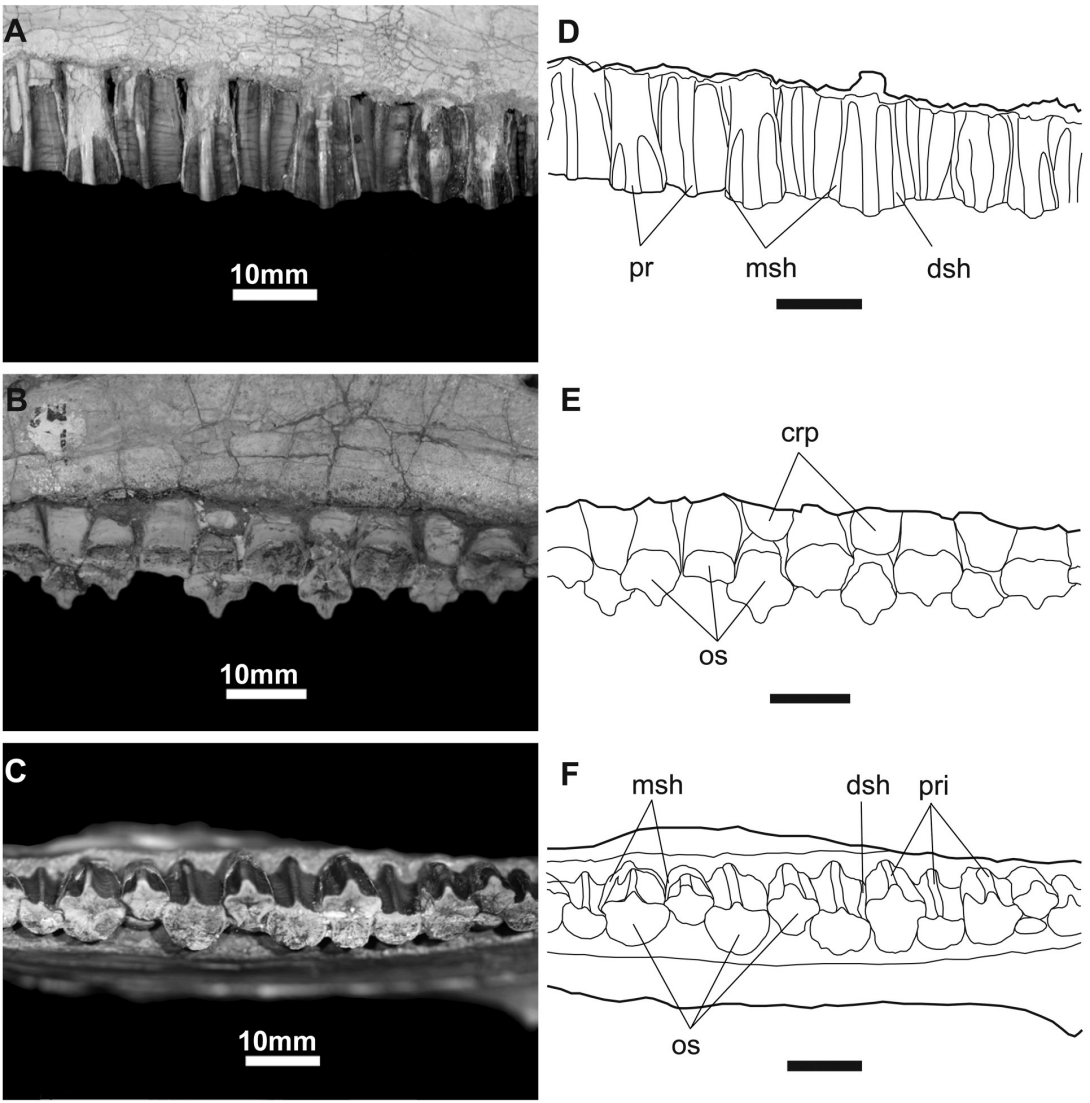
Maxillary teeth of *Gobihadros mongoliensis*. Maxillary teeth (MPC-D100/746) in buccal (A), lingual (B), and occlusal (C) views. Interpretative drawing in buccal (D), lingual (E), and occlusal (F) views. Abbreviations: crp, crown of replacement tooth; dsh, distal shelf; msh, mesial shelf; os, occlusal surface; pr, primary ridge.

*Maxillary teeth* (Fig 16): The crowns of the maxillary dentition are slightly asymmetrical and taller than wide in buccal view. The apex is located slightly distal to the midline of the crown. Lenticular in cross-section, they are enameled on their buccal sides. The primary ridge is prominent, but rounded in cross-section. There are no secondary ridges or marginal denticles on the crown, like the situation in *Probactrosaurus*, *Jeyawati*, *Protohadros*, *Bactrosaurus*, *Levnesovia*, *Eolambia*, *Tethyshadros*, *Telmatosaurus*, *Lophorhothon*, and all hadrosaurids [10, 17, 46, 51, 67, 68].

Maxillary teeth are closely packed, emplaced in an *en echelon* manner, and largest toward the middle of the tooth row. There are probably more than two teeth per tooth position, but there is only one functional tooth. Wear facets cover the entire occlusal surface and a dentine lip is found on the mesiolingual edge of each tooth. Transversely, facets make an angle of approximately 45° degrees with the horizontal plane.

**Fig 17:**
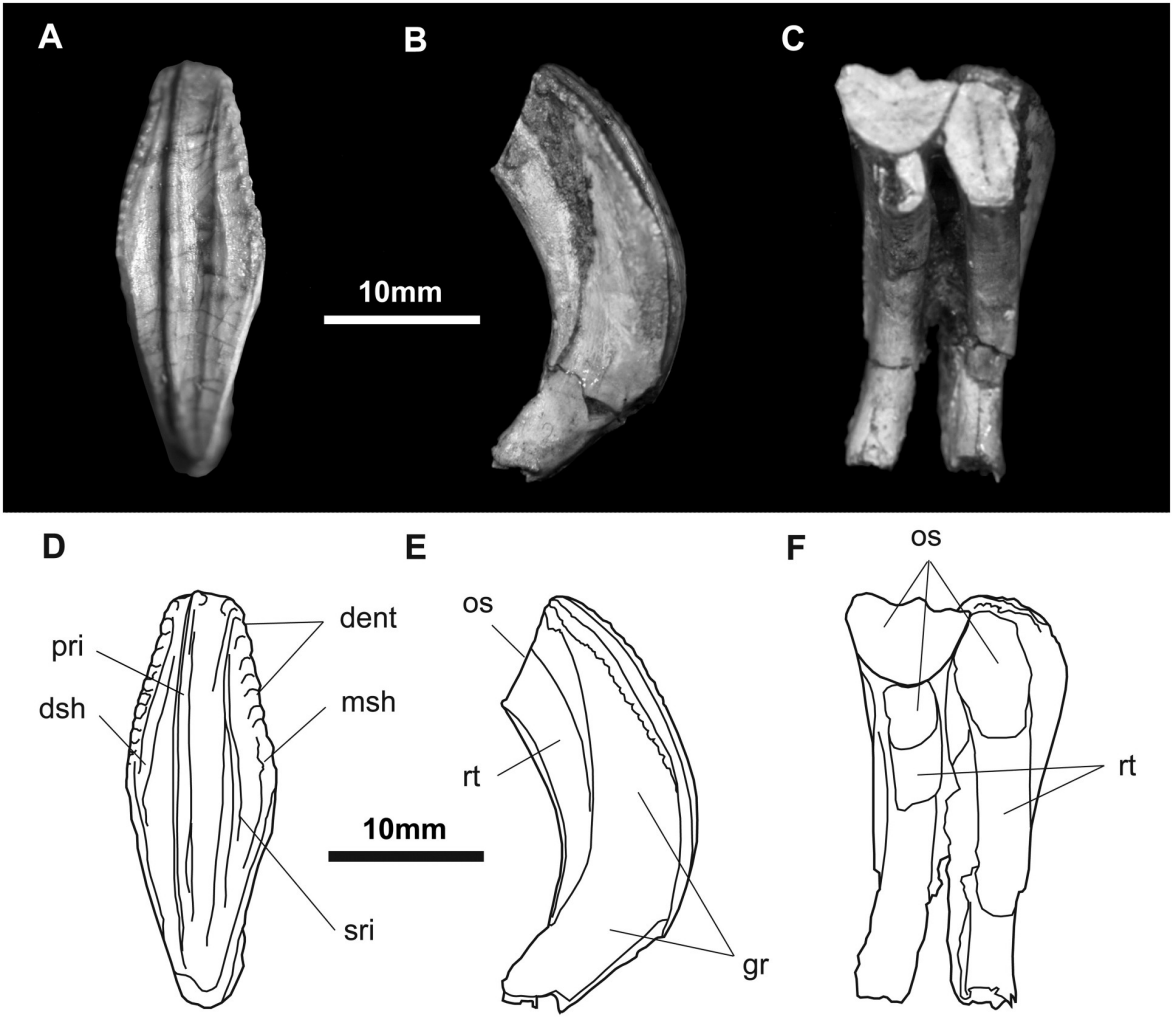
Dentary tooth of *Gobihadros mongoliensis*. Dentary tooth (MPC-D100/746, left) in lingual (A), mesial (B), and buccal (C) views. Interpretive drawing in lingual (D), mesial (E), and buccal (F) views. Abbreviations: dent, marginal denticles of crown; dsh, distal shelf; gr, grooves to accommodate the margins of crown of replacement tooth; msh, mesial shelf; os, occlusal surface; pr, primary ridge; rt, replacement roots; sr, secondary ridge.

*Dentary teeth* (Fig 17): The dentary dentition is only slightly different from the maxillary dentition. Each bears a vertical primary ridge disposed slightly asymmetrically on the crown. The largest teeth are found distal to the middle of the tooth row, similar to that of the maxillary dentition. Emplacement is *en echelon* and the teeth are closely packed into a dental battery in which there are up to two replacement teeth per position, similar to *Eolambia*, *Probactrosaurus*, *Jeyawati*, and *Bactrosaurus* [7, 10, 46, 52, 56], however, unlike these taxa but like hadrosaurids, there are up to three functional teeth in some of the tooth positions (hadrosaurids have up to five teeth per tooth family and as many as three functional teeth at a time). These teeth are ornamented on the margins of the lingual surface by small denticles and one or two secondary ridges mesial and distal to the primary carina, much like *Eolambia* and *Probactrosaurus* [10, 46]. Wear is found across each tooth, forming a continuous occlusal surface across the dentition. The angle of wear varies between 45° and 50° from the horizontal.

*Palpebral* (Fig 2): The palpebral articulates solely with the prefrontal and extends 70% across the orbit. A small shelf on the postorbital may indicate the caudal attachment of a cartilaginous extension of the palpebral.

*Sclerotic ring* (Fig 2): Several irregularly-shaped sclerotic plates are found within the orbit of MPC-D100/763, but their number and shape cannot be determined.

*Hyoid apparatus* (Fig 2): A pair of ceratobranchials is preserved; they are slightly curved, with a blunt distal end and an expanded proximal end. At midshaft, is bears a near-longitudinal groove on its dorsal side.

#### Postcranium

**Fig 18:**
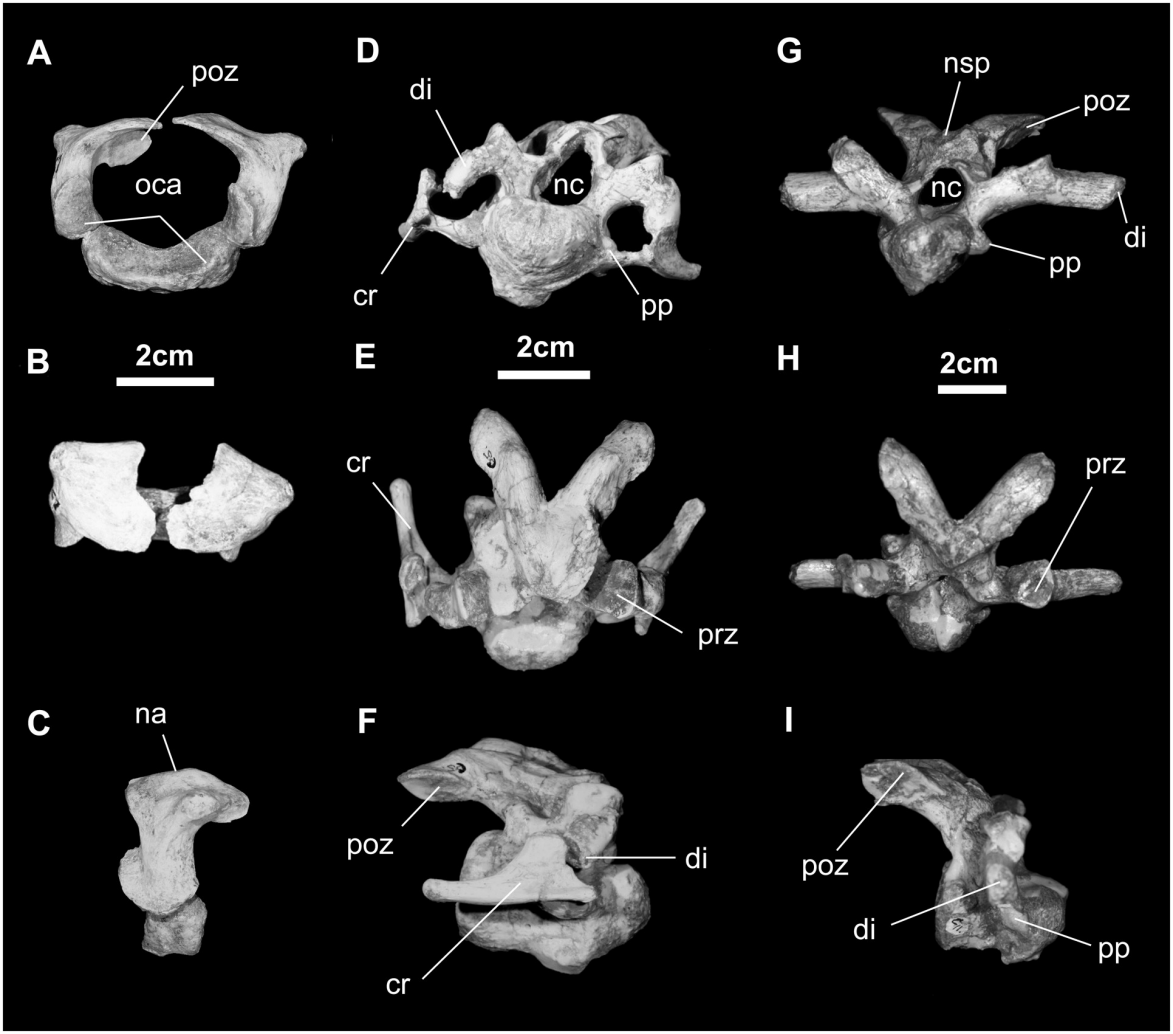
Cervical vertebrae (MPC-D100/746) of *Gobihadros mongoliensis*. Atlas in cranial (A), dorsal (B), and left lateral (C) views. Cranial cervical vertebra (c5) in cranial (D), dorsal (E), and right lateral (F) views. Caudal cervical vertebra (c11) in cranial (G), dorsal (H), and right lateral (I) views. Abbreviations: cr, cervical rib; di, diapophysis; na, neural arch; nc, neural canal; nsp, neural spine; oca, occipital condyle articulation; poz, postzygapophysis; pp, parapophysis; prz, prezygapophysis.

*Cervical vertebrae and ribs* (Fig 18): *Go*. *mongoliensis* has a total of 11 cervical vertebrae (MCP-D100/746; MCP-D100/753), although the axis is missing. The atlas consists of a single neurocentrum articulating with two pleurocentra. The latter are separated from each other dorsally. They each bear a postzygapophyseal process that articulates with the axis. The inner curvature of the neurocentrum articulates with the dens of the axis.

Cervical vertebrae in the middle of the series have ovate centra in cranial view. They are strongly opisthocoelous, much like the condition seen in numerous hadrosauroids including all hadrosaurids, *Lophorhothon*, *Claosaurus*, *Tanius*, *Bactrosaurus*, *Jeyawati*, *Probactrosaurus*, *Eolambia*, *Equijubus*, *Jinzhousaurus*, *Ouranosaurus*, *Mantellisaurus*, *Iguanodon*, and more basal forms [7, 10, 14, 15, 44, 46–48, 51, 52, 54, 68, 69]. Laterally, the parapophysis contacts the elongate tuberculum of the cervical rib, forming with the transverse process and the head of the rib, a large transverse foramen. Axial length of the centra axial is approximately 10% longer than the transverse width. An axially oriented keel traverses the entire ventral surface. The prezygapophysis, formed directly above the transverse process, is short, while the postzygapophysis arches over the intervertebral joint to contact the adjacent prezygapophysis. The angle of both pre- and postzygapophyseal facets is approximately 15°-20° from horizontal. The neural spine is very low to absent.

Toward the cranial part of the dorsal series, the cervical vertebrae have longer and more robust postzygapophyses and transverse processes, which project nearly directly laterally. Otherwise, the features of these cervicals are much like those with a more cranial position.

The cervical ribs of *Go*. *mongoliensis* are typical of hadrosauroids: relatively straight, short shaft that does not exceed the length of its centrum, and a large capitulum and tuberculum.

**Fig 19:**
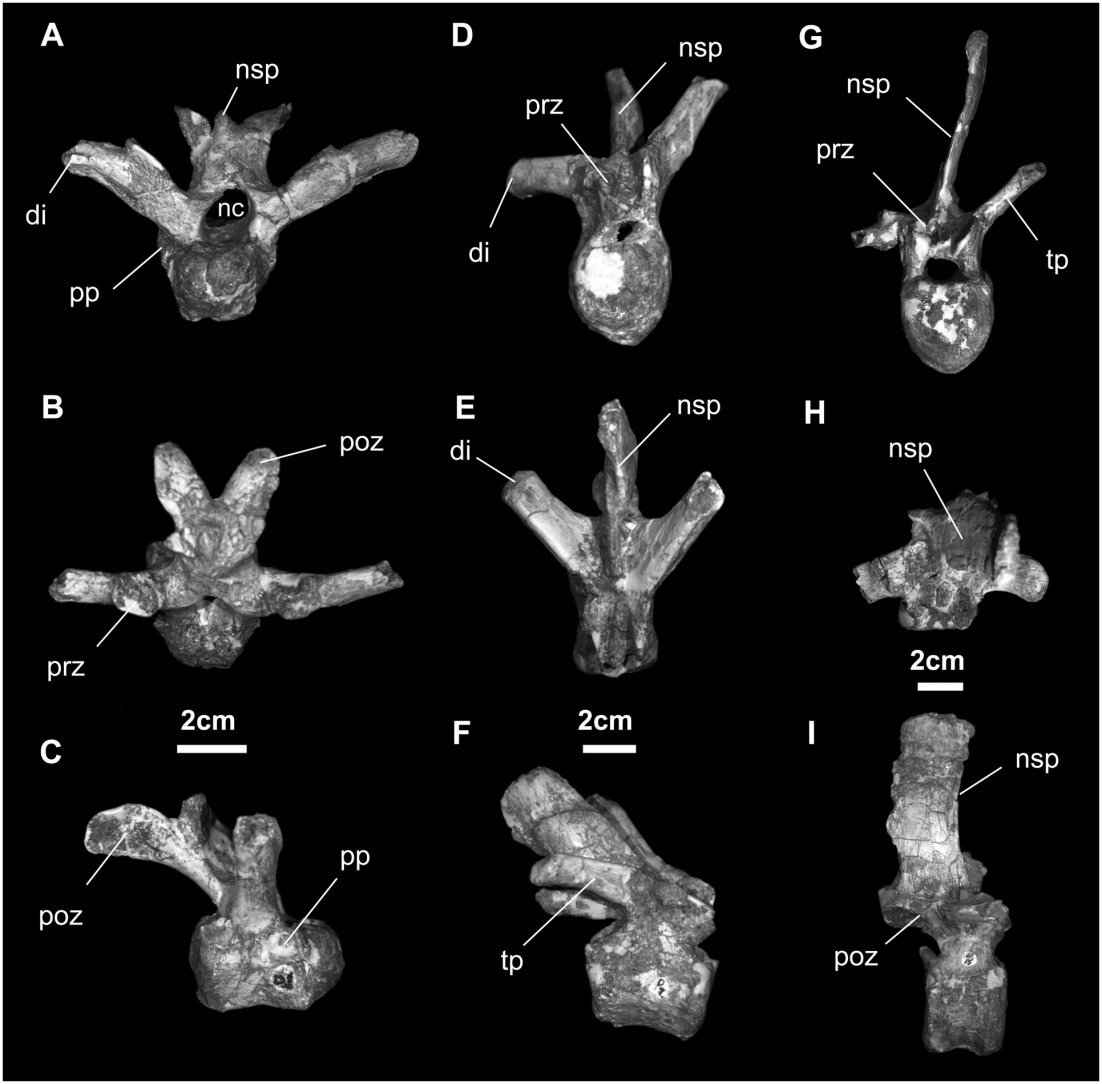
Dorsal vertebrae and ribs (MPC-D100/746) of *Gobihadros mongoliensis*. Cranial dorsal vertebra (D1) in cranial (A), dorsal (B), and right lateral (C) views. Cranial-to-middle dorsal vertebra (D7) in cranial (D), dorsal (E), and right lateral (F) views. Middle-to-caudal dorsal vertebra (D15) in cranial (G), dorsal (H), and right lateral (I) views. Abbreviations: di, diapophysis; nc, neural canal; nsp, neural spine; pp, parapophysis; poz, postzygapophysis; prz, prezygapophysis; tp, transverse process.

*Dorsal vertebrae and ribs* (Fig 19): Sixteen dorsal vertebrae are known in *Go*. *mongoliensis* (MPC-D100/746, MCP-D100/753). At the front of the series, the centrum is spool-shaped in ventral view, nearly twice as long as wide. The transverse processes are long and angled dorsolaterally and caudally. The neural spine is only as high as is the centrum and is angled approximately 50° dorsocaudally. The parapophysis is found on the upper centrum and onto the base of the neural arch.

The centrum of the more caudal dorsal vertebrae is slightly higher than wide and long. The angle of the zygapophyseal facets is approximately 35°-40°. The transverse processes project at an angle of about 45° from the horizontal. The parapophysis has migrated onto the side of the neural arch. The neural spine is transversely and axially narrow (more than 40% its height); it is twice as high as the centrum and nearly vertical in orientation.

The cranial dorsal ribs differ little from the cervical ribs, although down the series they become much longer and project more vertically. The longest is the sixth rib, after which they decrease in length. Most end bluntly, indicating articulation with the costal cartilages.

**Fig 20:**
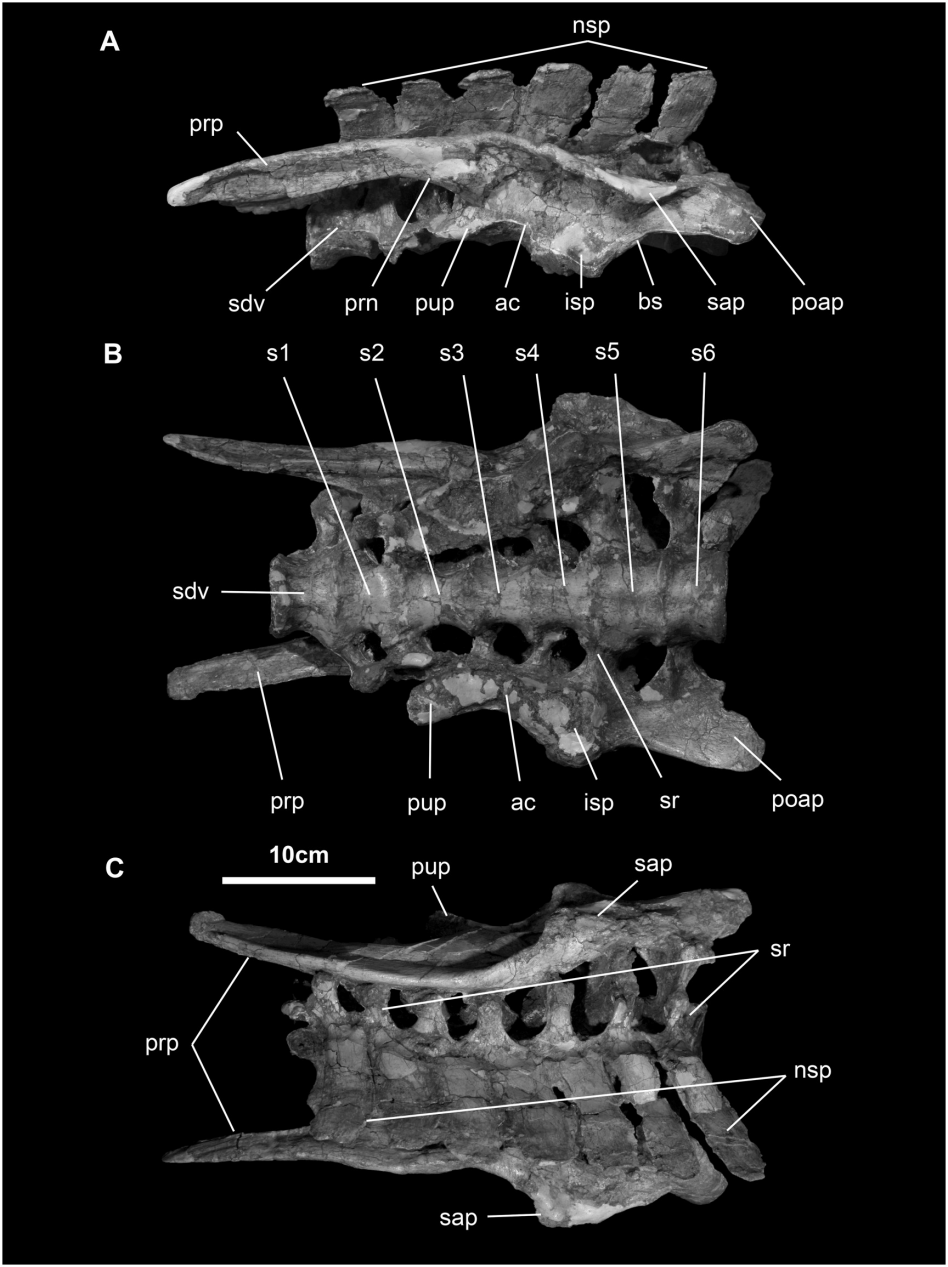
Sacral block with ilia of *Gobihadros mongoliensis*. Sacral block with ilia (MPC-D100/746) in left lateral (A), ventral (B), and dorsal (C) views. Abbreviations: ac, acetabulum; isp, ischial peduncle; nsp, neural spine; bs, brevis shelf (postacetabular notch); poap, postacetabular process; prn, preacetabular notch; prp, preacetabular process; pup, pubic peduncle; sap, supraacetabular process; sdv, sacrodorsal vertebra; sr, sacral ribs; s1—s6, first through sixth sacral vertebrae.

*Sacral vertebrae and ribs* (Fig 20): The sacrum of *Go*. *mongoliensis* consists of seven coossified sacral vertebrae (1 dorsosacral and 6 true sacrals; MCP-D100/746, MCP-D100/753). The neural spines and transverse processes are short, the latter capped with massive sacral ribs that articulate with nearly the entire medial surface of the ilium.

**Fig 21:**
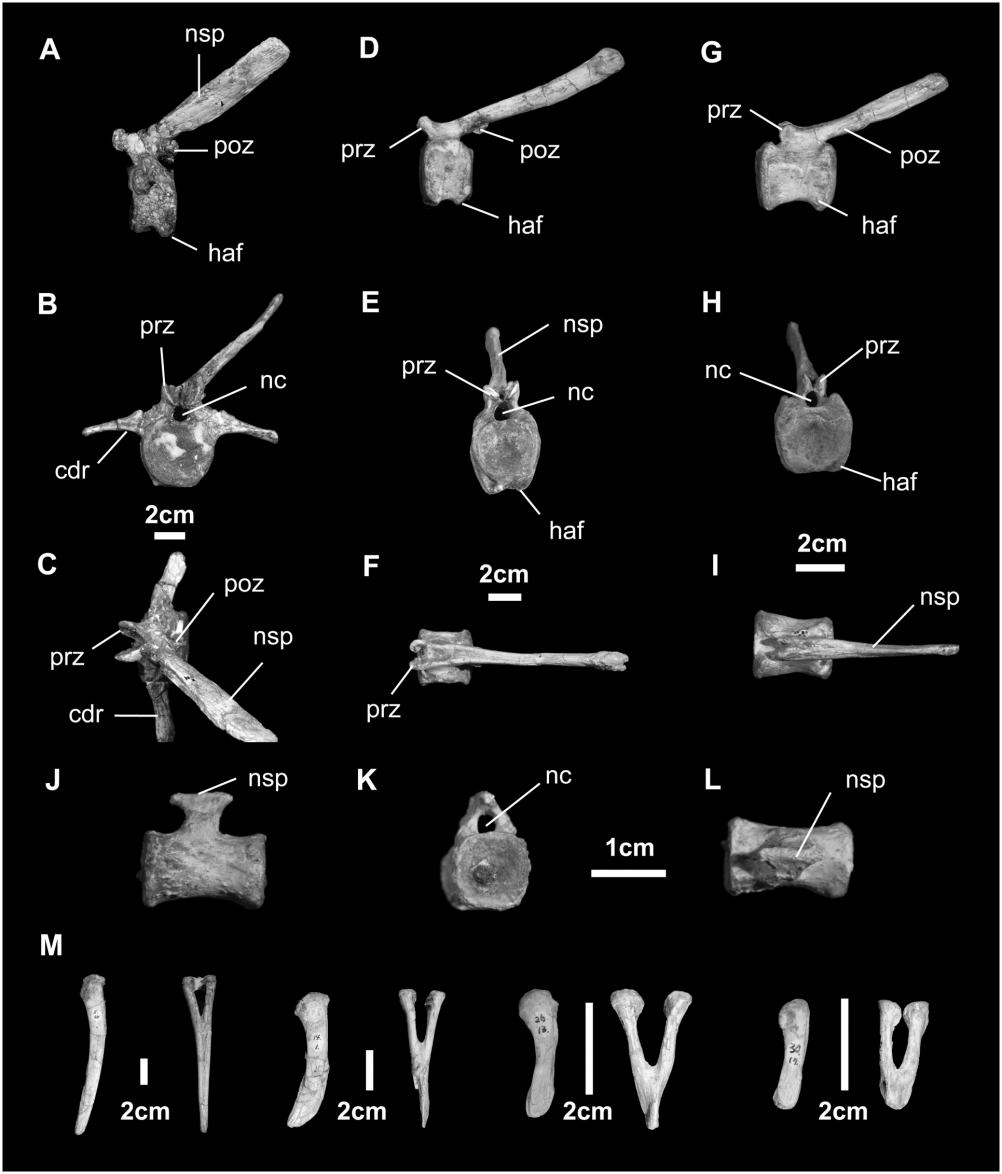
Caudal vertebrae and chevrons (MPC-D100/746) of *Gobihadros mongoliensis*. Proximal caudal vertebra (Cd2) in left lateral (A), proximal (B), and dorsal (C) views. Proximal-to-middle caudal vertebra (Cd13) in left lateral (D), proximal (E), and dorsal (F) views. Middle caudal vertebra (Cd28) in left lateral (G), proximal (H), and dorsal (I) views. Middle-to-distal caudal vertebra (Cd40) in left lateral (M), proximal (N), and dorsal (O) views. Distal caudal vertezbra (Cd55) in left lateral (M), proximal (N), and dorsal (O) views. Chevrons (ch5, ch14, ch26 and ch30) in right lateral and proximal (P) views. Abbreviations: cdr, caudal rib; haf, haemal arch (chevron) facet; nc, neural canal; nsp, neural spine; poz, postzygapophysis; prz, prezygapophysis.

*Caudal vertebrae and chevrons* (Fig 21): There are 58 caudal vertebrae in *Go*. *mongoliensis* (MCP-D-100/746). Proximal caudal vertebrae have a short, wide, and nearly circular centrum. Proximally, the central surface is slightly biconcave, while distally it is slight biconvex. There is a hemifacet at the base of the front and back surfaces to accommodate the hemal arch. The neural spine rises nearly twice the height of the centrum and projects 45° dorsodistally. The pre- and postzygapophyseal process are prominent, angling approximately 50° to the horizontal. Middle caudal vertebrae have centra that are nearly as long as high. The hemifacets at both ends of the ventral surface are prominent. Separating the two is an axial groove. The neural spine is long (250% centrum height), slightly expanded distally, and angled 30° dorsodistally. The pre- and postzygapophyseal facets make an angle of 60°-65° to the horizontal. Transverse processes are absent. Distal caudal vertebrae are cylindrical, equidimensional in proximal view. There is no neural spine topping the neural arch and no transverse processes. The centrum is twice as long as high. A notochordal pit is visible on both proximal and distal surfaces.

Chevrons articulate via hemifacets associated with intervertebral spaces, from the distal end of the second caudal vertebra onward. Each consisting of a long, slender distal blade, with a proximal canal surrounded by the hemal arch, the chevrons regularly decrease in size down the tail.

**Fig 22:**
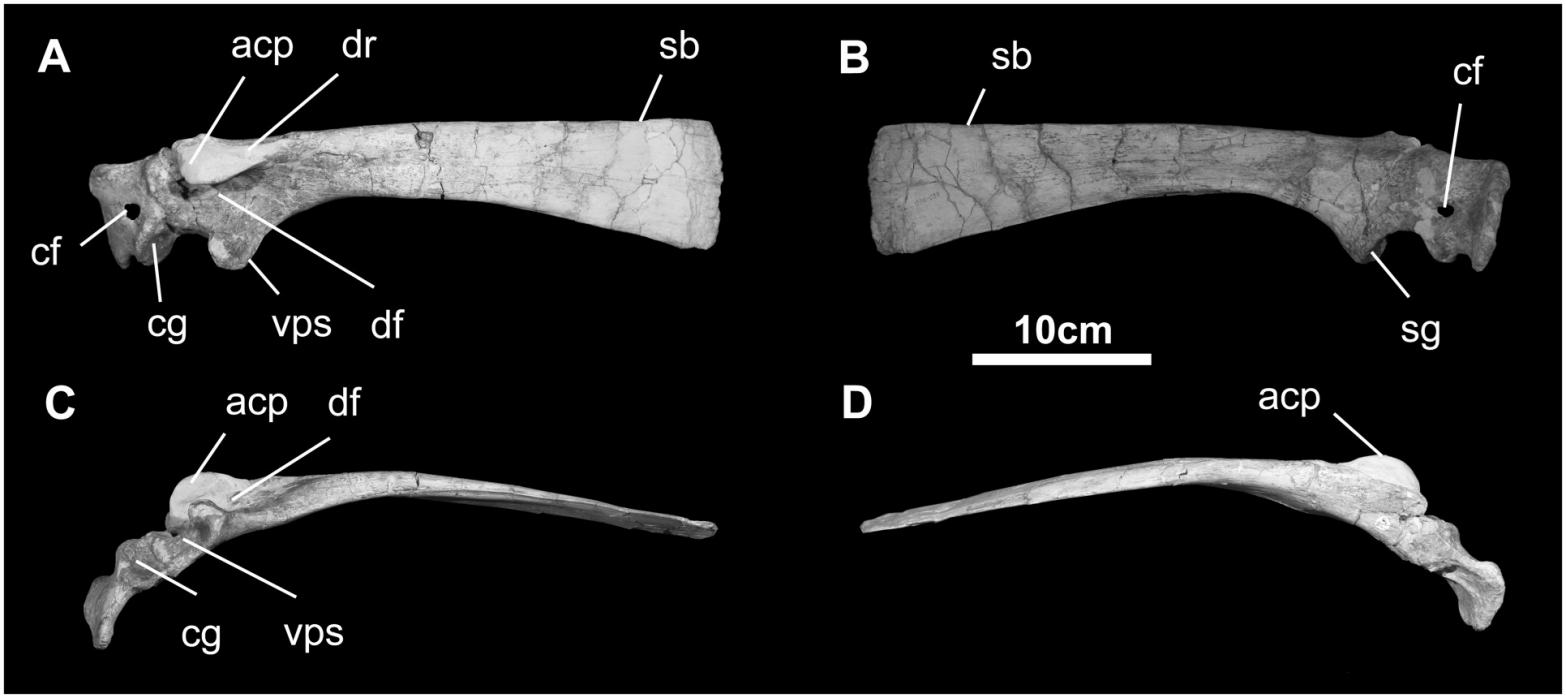
Scapula and coracoid of *Gobihadros mongoliensis*. Scapula and coracoid (MPC-D100/746, left) in lateral (A), medial (B), ventral (C), and dorsal (D) views. Abbreviations: acp, acromion process; cf, coracoid foramen; cg, coracoid glenoid; df, deltoid fossa; dr, deltoid ridge; sb, scapular blade; sg, scapular glenoid; vps, ventral protuberance of the scapula.

*Scapula* (Fig 22): The scapular blade is long and slightly divergent caudally (much like in *Iguanodon*, *Tanius*, *Ouranosaurus*, *Probactrosaurus*, and *Altirhinus*; [44–46, 48, 54], abruptly ending in a roughly orthogonal distal margin, representing the widest dimension of the scapula. The blade is internally concave, reflecting the curvature of the rib cage. The straighter dorsal margin of the scapular blade terminates cranially as a prominent, laterally-projecting pseudoacromial process (attachment of *m*. *deltoids clavicularis*), just above the craniodorsal aspect of the scapulocoracoid articulation. The caudoventral aspect of this region is occupied by the dorsal lip of the glenoid articulation with the humerus (attachment of *m*. *triceps scapulare lateralis externum*).

*Coracoid* (Fig 22): The coracoid is found in articulation with the scapula. It is a relatively large element that contributes the ventral lip of the glenoid. Beneath this position, the coracoid notch is shallow and the coracoid process is short. The coracoid foramen is found toward the center of the element and does not intersect the scapulocoracoid articulation on external and internal surfaces. The cranial margin of the coracoid is straight to weakly convex and the biceps tubercle is small.

**Fig 23:**
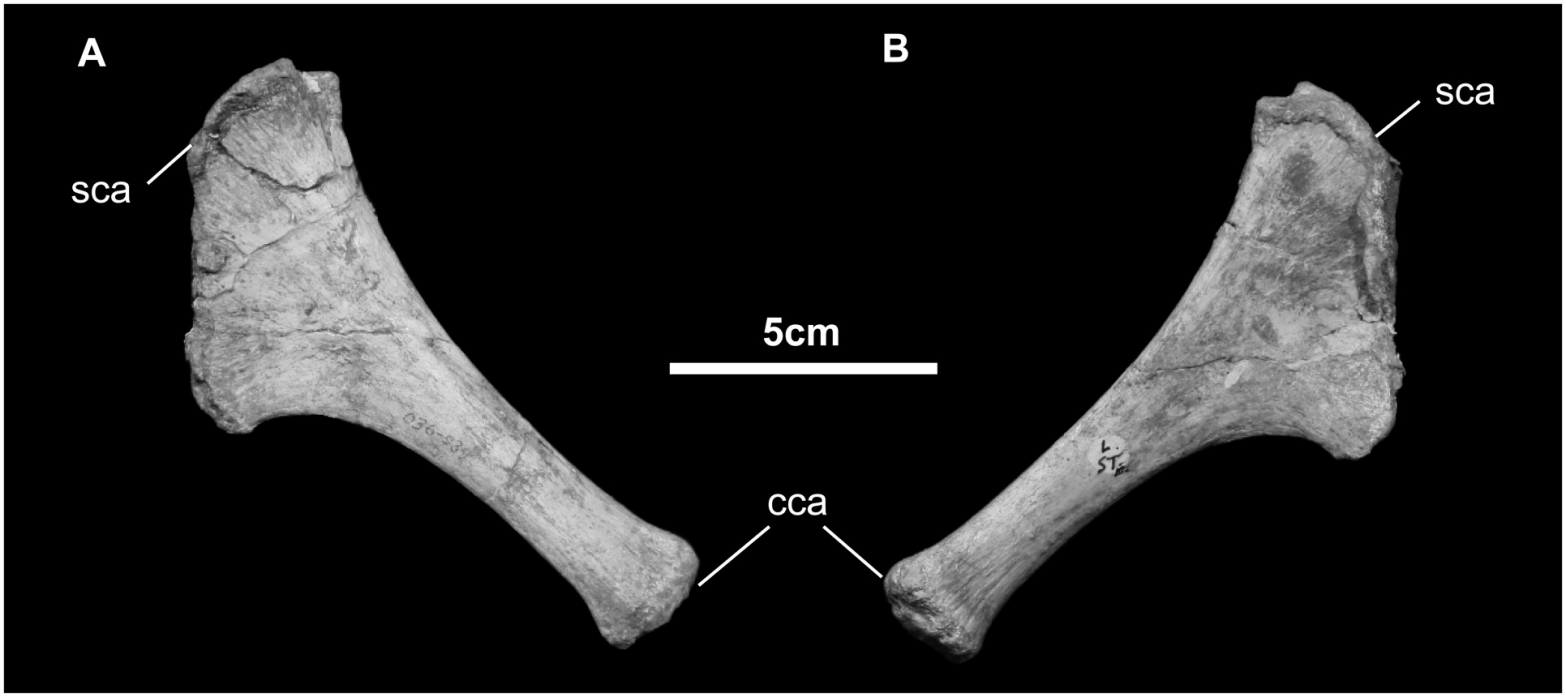
Sternal plate of *Gobihadros mongoliensis*. Sternal (MPC-D100/746) in ventral (A) and dorsal (B) views. Abbreviations: cca, costal cartilage attachment; sca, sternal cartilage attachment.

*Sternal* (Fig 23): The sternal of *Go*. *mongoliensis* is much like other hadrosauroids. The caudal process is slightly longer than the cranial sternal body, as in *Fukuisaurus*, *Iguanodon*, *Ouranosaurus*, *Eolambia*, and *Jinzhousaurus* [10, 14, 16, 44, 48, 70].

**Fig 24:**
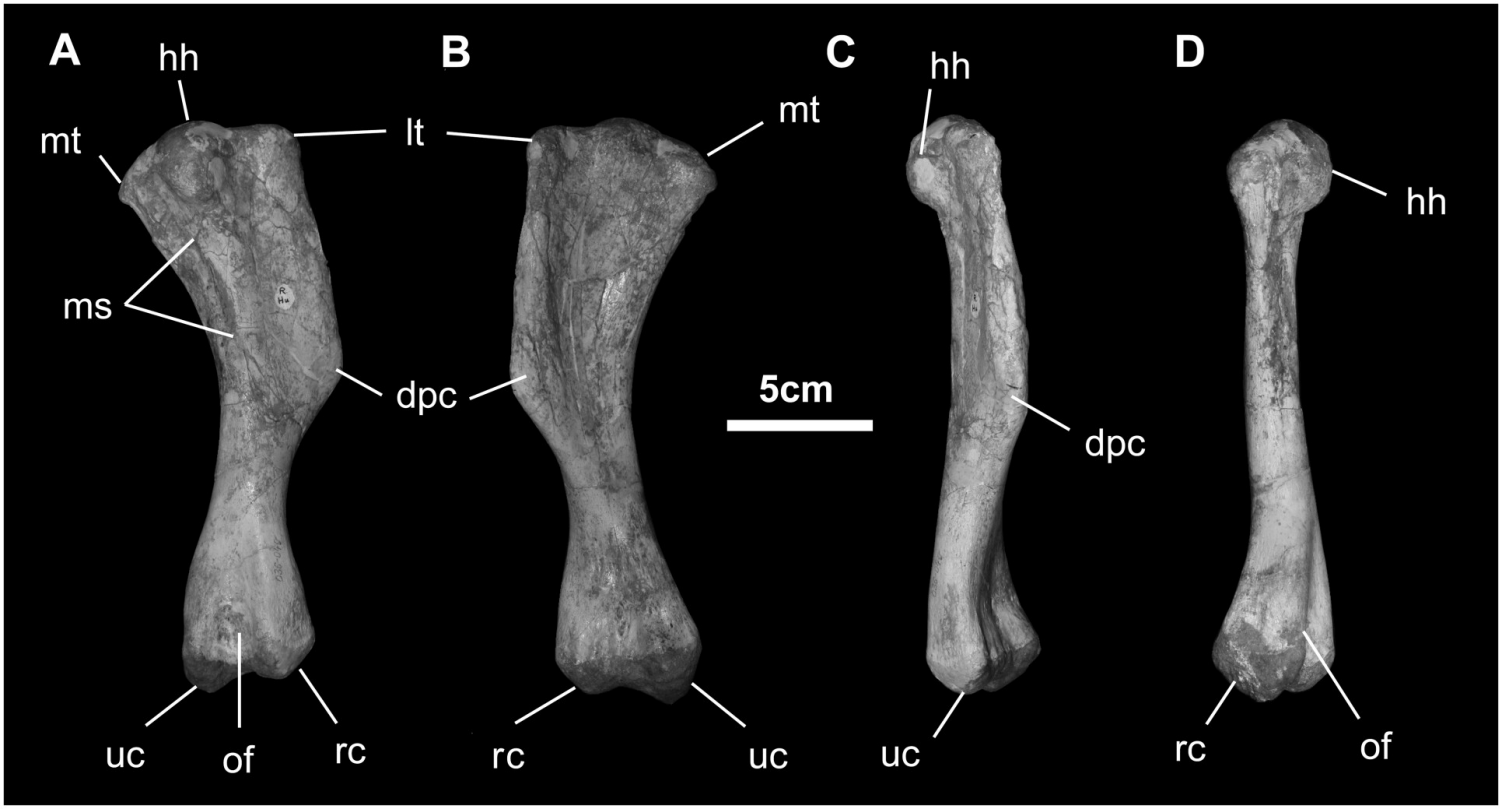
Humerus of *Gobihadros mongoliensis*. Humerus (MPC-D100/746, right) in posterior (A), anterior (B), lateral (C), and medial (D) views. Abbreviations: dpc, deltopectoral crest; hh, humeral head; lt, lateral tuberosity; ms, muscle scar; mt, medial tuberosity; of, olecranon fossa; rc, radial condyle; uc, ulnar condyle.

*Humerus* (Fig 24): The humerus in lateral view is dominated by the deltopectoral crest, the roughened apex of which is found at midshaft. The crest (attachment of *m*. *deltoids clavicularis*) extends 25–30% beyond the diameter of the shaft. The head is globose, wrapping onto the caudal surface of the humerus. The lateral tuberosity is broad where it merges with the proximal portion of the deltopectoral crest. The medial tuberosity is smaller and merges indistinctly with the humeral head. The caudal surface of the humerus is strongly marked by the scar for *mm*. *teres major* and *latissimus dorsi*. Below the deltopectoral crest, the shaft is nearly circular. The distal condyles of the humerus are transversely broad, flaring moderately from the shaft. The ulnar condyle projects slightly more distally and is larger than the radial condyle. The two condyles are separated by shallow cubital and olecranon fossae.

**Fig 25:**
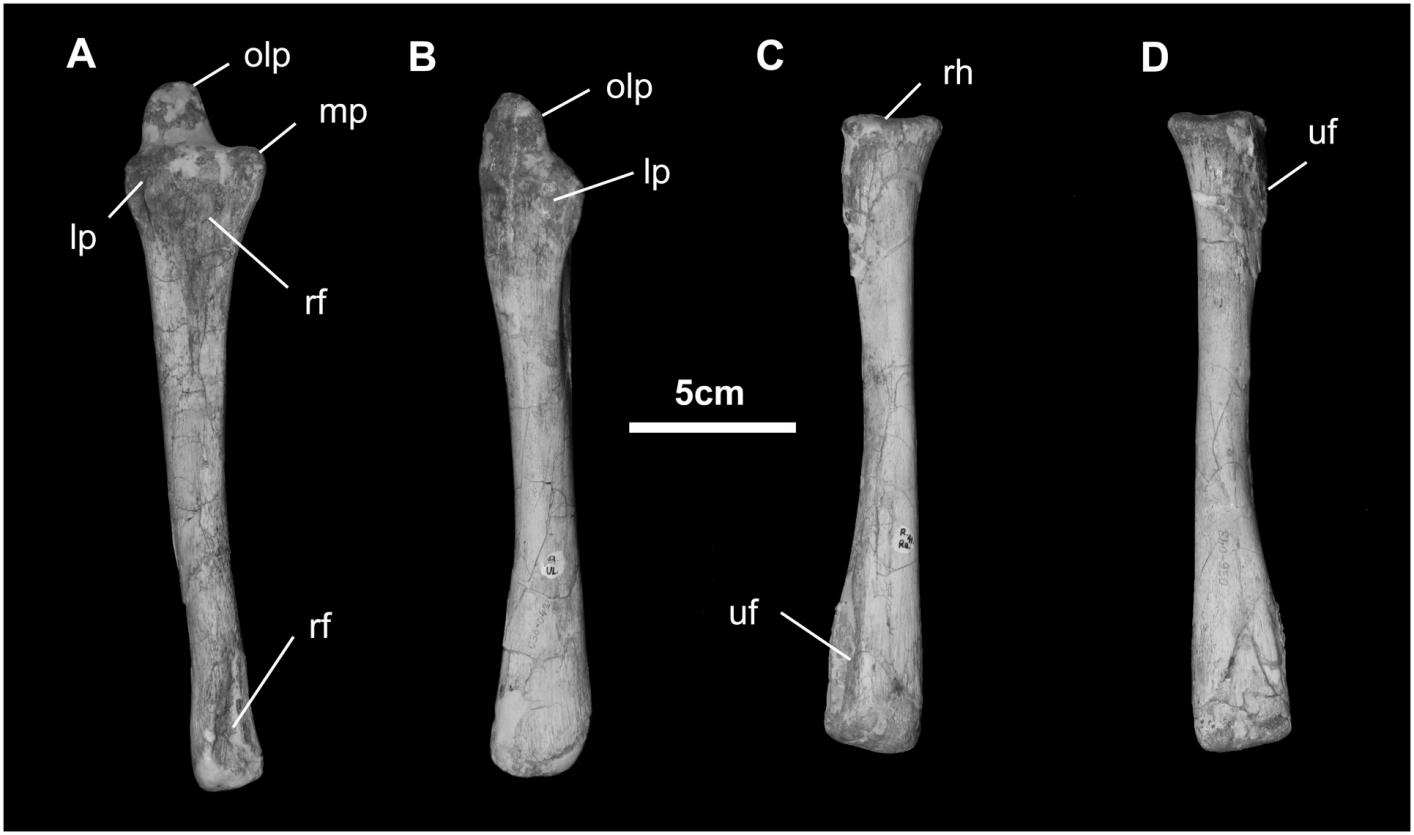
Ulna and radius (MPC-D100/746, right) of *Gobihadros mongoliensis*. Ulna in anterior (A) and lateral (B) views. Right radius in lateral (C) and medial (D) views. Abbreviations: lp, lateral process; mp, medial process; olp, olecranon process; rf, radial facet; rh, radial head; uf, ulnar facet.

*Radius* (Fig 25): The gracile radius is approximately as long as the humerus. The proximal surface of the radial head is flat where it articulates with the humerus. The upper articular surface for the proximal ulna is somewhat triangular. The radial tuberosity is only slightly developed. The shaft is straight and marked by the interosseous ridge. The radius terminates distally as a slight flaring for articulation with the distal ulna laterally and the carpals distally.

*Ulna* (Fig 25): The ulna is a slightly curved element. Proximally, the small olecranon extends behind the ulnar articulation with the humerus. The latter is deep and strongly concave in lateral view. Proximally, the ulna curls around the head of the radius. Distally, the ulna terminates abruptly where it forms the ulnar-carpal articulation.

**Fig 26:**
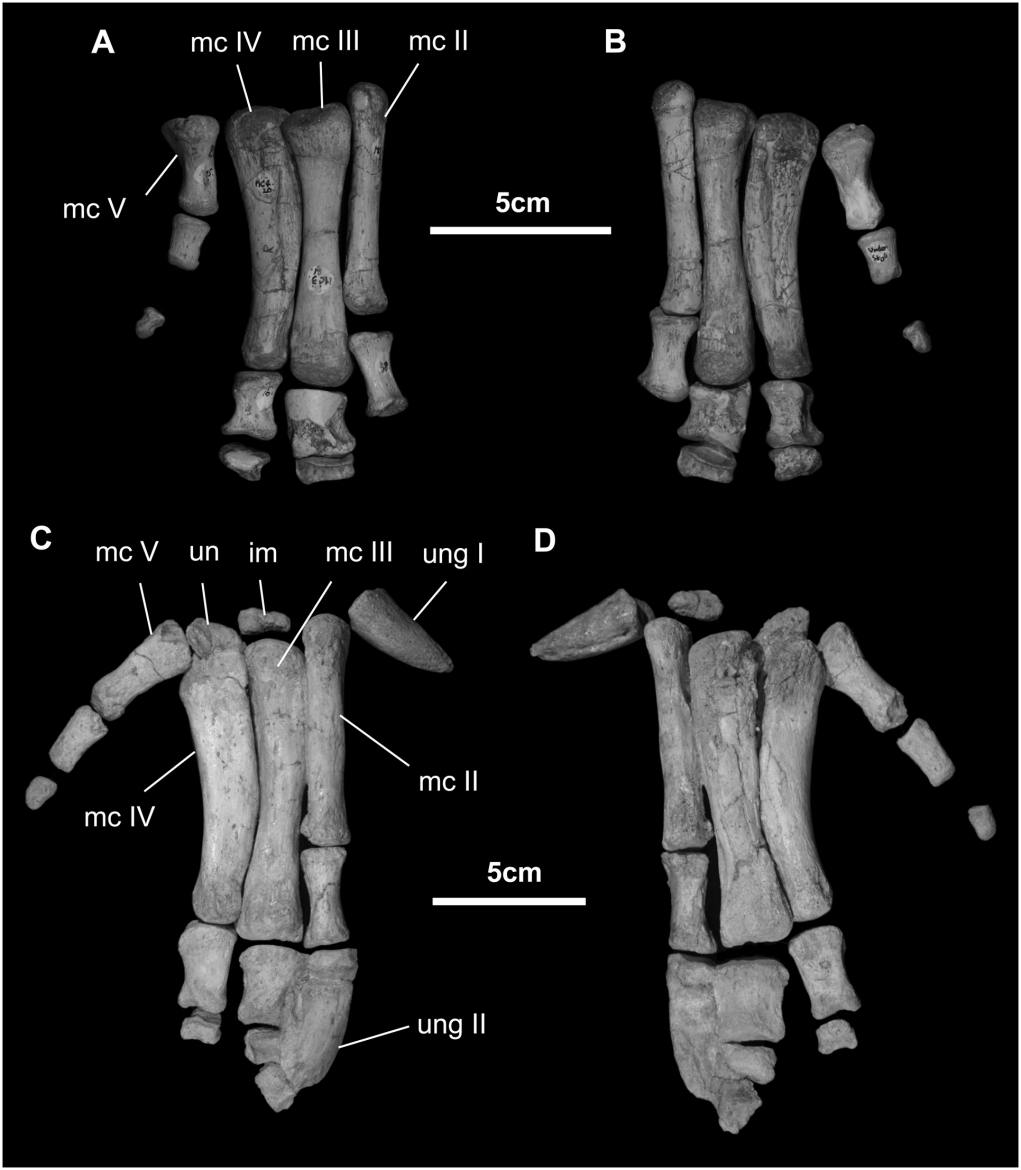
Manus of *Gobihadros mongoliensis*. Manus (MPC-D100/746, right) in dorsal (A) and ventral (B) views. Manus (MPC-D100/763, right) in dorsal (C) and ventral (D) views. Abbreviations: im, intermedium; mc-II, metacarpal-II; mc-III, metacarpal-III; mc-IV, metacarpal-IV; mc-V, metacarpal-V; un, ulnare; ung-I, ungual of digit-I; ung-II, ungual of digit-II.

*Manus* (Fig 26): The carpus in *Go*. *mongoliensis* is reduced to two small, unfused nubbins. All of the metacarpals are elongate (midshaft width to length 0.15 or less). The proximal end of Metacarpal III is offset distally relative to MC II and IV. The digital formula is 1-3-3-3-4 (based on MPC-D-100/763). The penultimate phalanges of digits II and III are wedge-shaped and the medial side of the phalanges is significantly shorter than the lateral side.

*Ilium* (Fig 20): The ilium of *Go*. *mongoliensis* has a shallow sigmoid dorsal margin. The preacetabular process is narrow and long, making up nearly half of the entire element. In contrast to the ischial peduncle, the pubis peduncle is thin and long. On the other hand, the ischial peduncle is very round and bulbous. The supracetabular process is large and broadly overhangs the lateral side of the ilium, extending at least to a half way down the side, as in *Bactrosaurus*, *Tanius*, and all hadrosaurids [51, 52, 54]. The postacetabular process is less than 40% the length of the ilium and tapers caudally to nearly a point, with a wide brevis shelf on its ventral margin.

**Fig 27:**
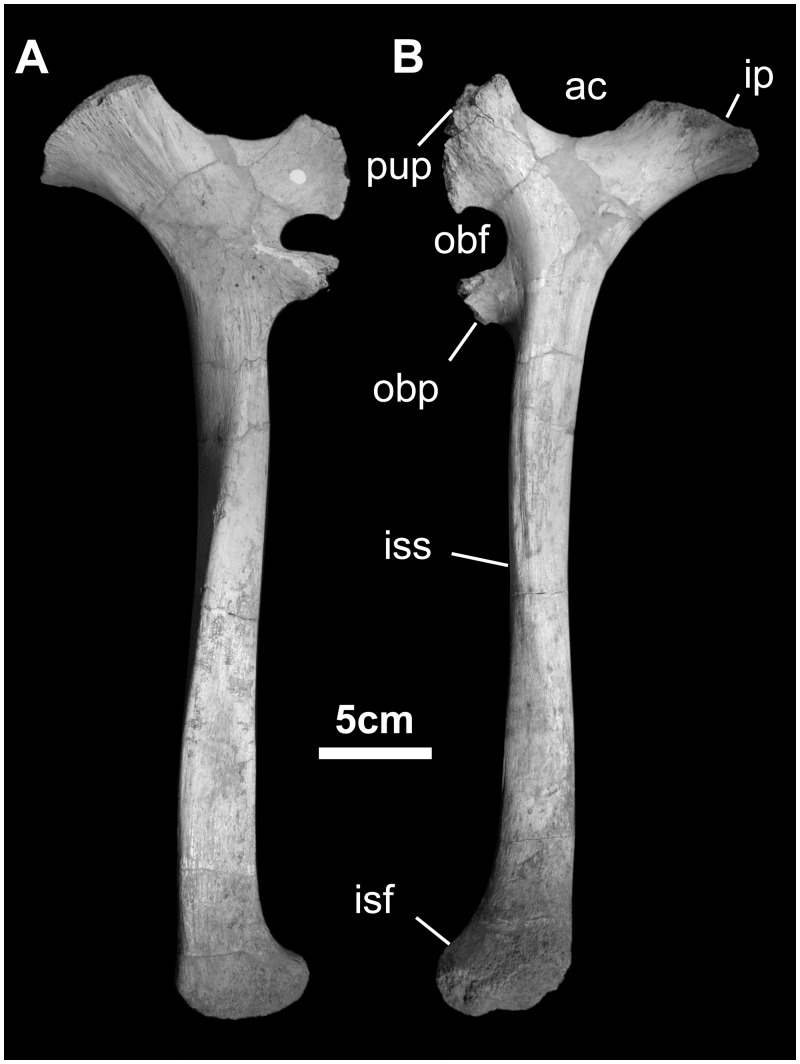
Ischium of *Gobihadros mongoliensis*. Ischium (MPC-D100/760, right) in medial (A) and lateral (B) views. Abbreviations: ac, acetabulum; ip, iliac peduncle; isf, ischial foot; iss, ischial shaft; obf, obturator foramen; obp, obturator process; pup, pubic peduncle.

*Ischium* (Fig 27): The ischium consists of the acetabular end, a long, straight shaft, and an expanded distal end. The proximal acetabular region consists of a robust iliac peduncle, a smoothly convex acetabular surface, and a wide pubic peduncle. Immediately distal to the pubic peduncle is the upper rim of the obturator foramen and the obturator process. The latter is found 22% down the ischial shaft. The lateral surface of the shaft is marked by an undulating ridge and groove, probably for *m*. *ischiotrochantericus*. The distal end of the ischium is enlarged, with a pendent foot, also found in *Bactrosaurus*, *Ouranosaurus*, all lambeosaurine hadrosaurids, and to a lesser extent in *Probactrosaurus*, *Mantellisaurus*, and *Iguanodon* [44, 46–48, 51, 52].

**Fig 28:**
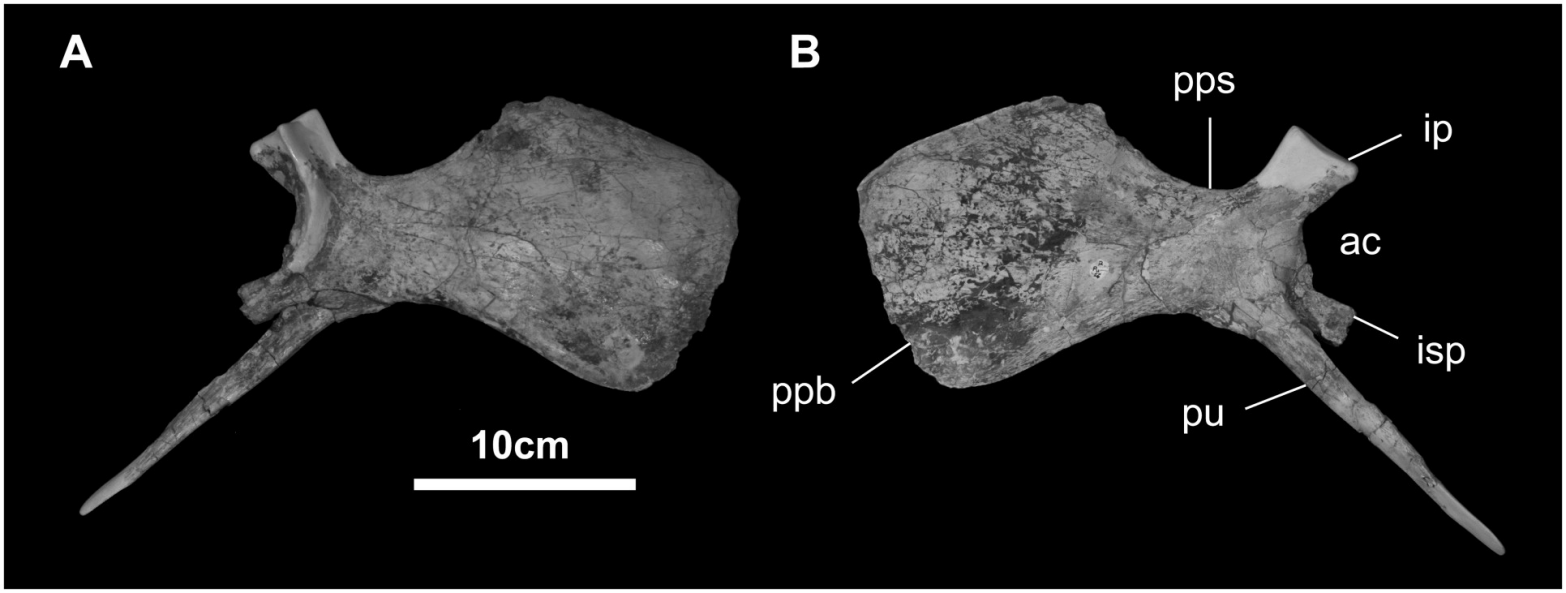
Pubis of *Gobihadros mongoliensis*. Pubis (MPC-D100/746, right) in lateral (A) and medial (B) views. Abbreviations: ac, acetabulum; ip, iliac peduncle; isp, ischial peduncle; ppb, prepubic blade; pps, prepubic shaft; pu, pubis.

*Pubis* (Fig 28): The pubis closely resembles that of *Bactrosaurus johnsoni* [52]. The style-like pubis *sensu stricto* is very short, approximately 66% the length of the prepubic process. The latter is semi-rectangular, expanded to more than twice the depth of its proximal shaft, as in hadrosaurids, *Huehuecanauhtlus*, *Tethyshadros*, *Levnesovia*, *Bactrosaurus*, *Probactrosaurus*, *Xuwulong*, *Altirhinus*, *Ouranosaurus*, *Delapparentia*, *Mantellisaurus*, and *Iguanodon*, among iguanodontoids [17, 18, 22, 44–48, 51, 52, 71, 72]. The prepubic shaft is relatively short. Both iliac and ischial peduncles are well preserved, the former slightly more robust than the latter.

**Fig 29:**
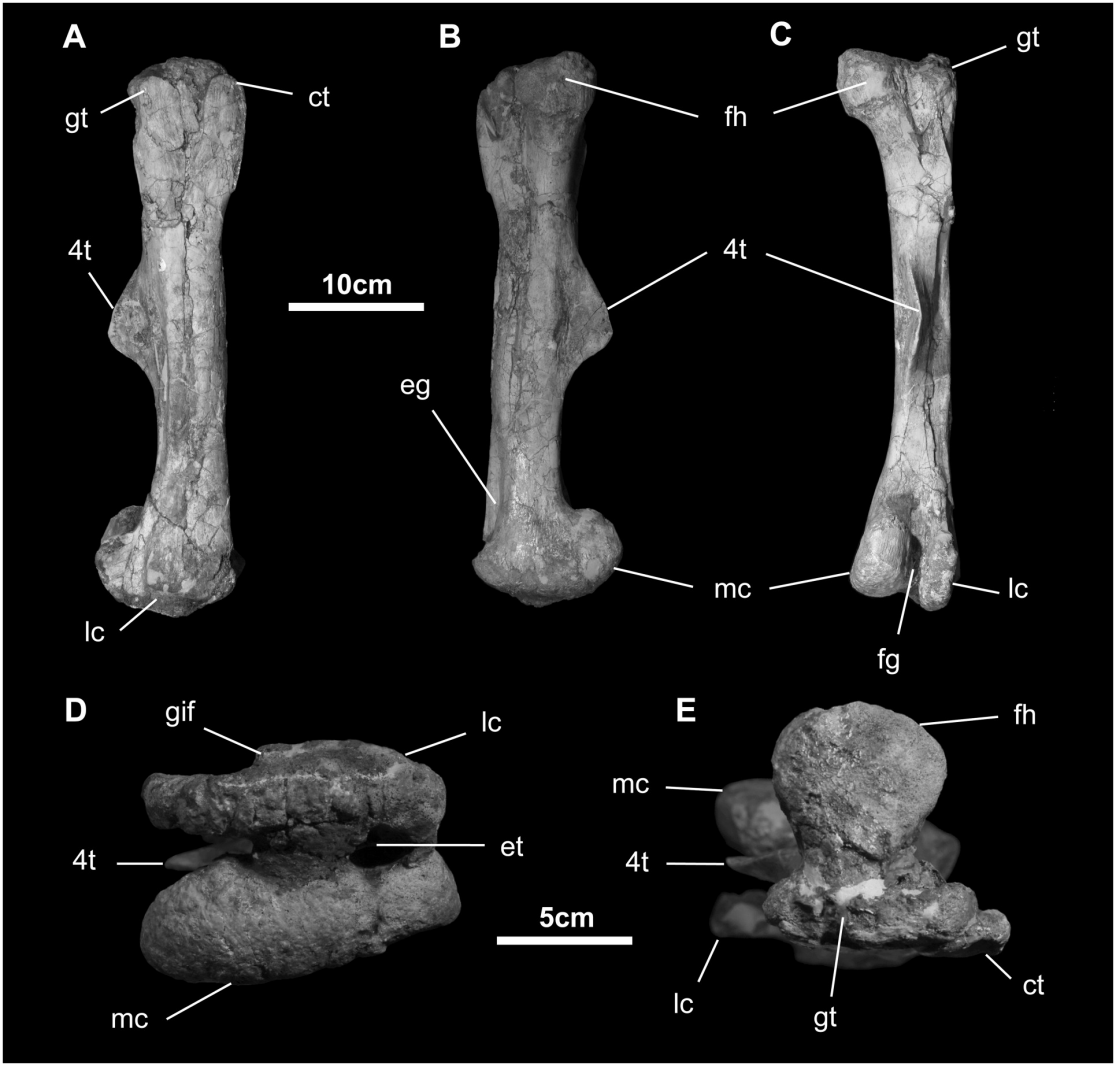
Femur of *Gobihadros mongoliensis*. Femur (MPC-D100/746, right) in lateral (A), medial (B), posterior (C), ventral (D), and dorsal (E) views. Abbreviations: ct, cranial trochanter; eg, extensor groove; et, extensor tunnel; fg, flexor groove; fh, femoral head; gif, groove for *m*. *iliofibularis*; gt, greater trochanter; lc, lateral condyle; mc, medial condyle; 4t, fourth trochanter.

*Femur* (Fig 29): The femur is robust in size and shape; midshaft diameter is 12% femoral length. The head is slightly set off from the shaft, but there is no distinct neck. The greater trochanter does not extend dorsally above the femoral head, while the cranial trochanter is large and set off from the cranial aspect of the shaft. The shaft itself is straight and dominated by the fourth trochanter, which is triangular in shape (the peak of the process is 50% down the shaft), much like the condition in *Iguanodon*, *Mantellisaurus*, *Ouranosaurus*, *Jinzhousaurus*, *Probactrosaurus*, and *Nanyangosaurus* [14, 44, 46–48, 73]. Distally, the lateral condyle is much narrower than the medial condyle and bears the incisure for the tendon of *m*. *iliofibularis*. On the cranial surface, between the two condyles is a deep intercondylar extensor groove that is bridged over by the cranial extension of the condyles to form a tunnel. Caudally, the flexor groove between the condyles is relatively spacious.

**Fig 30:**
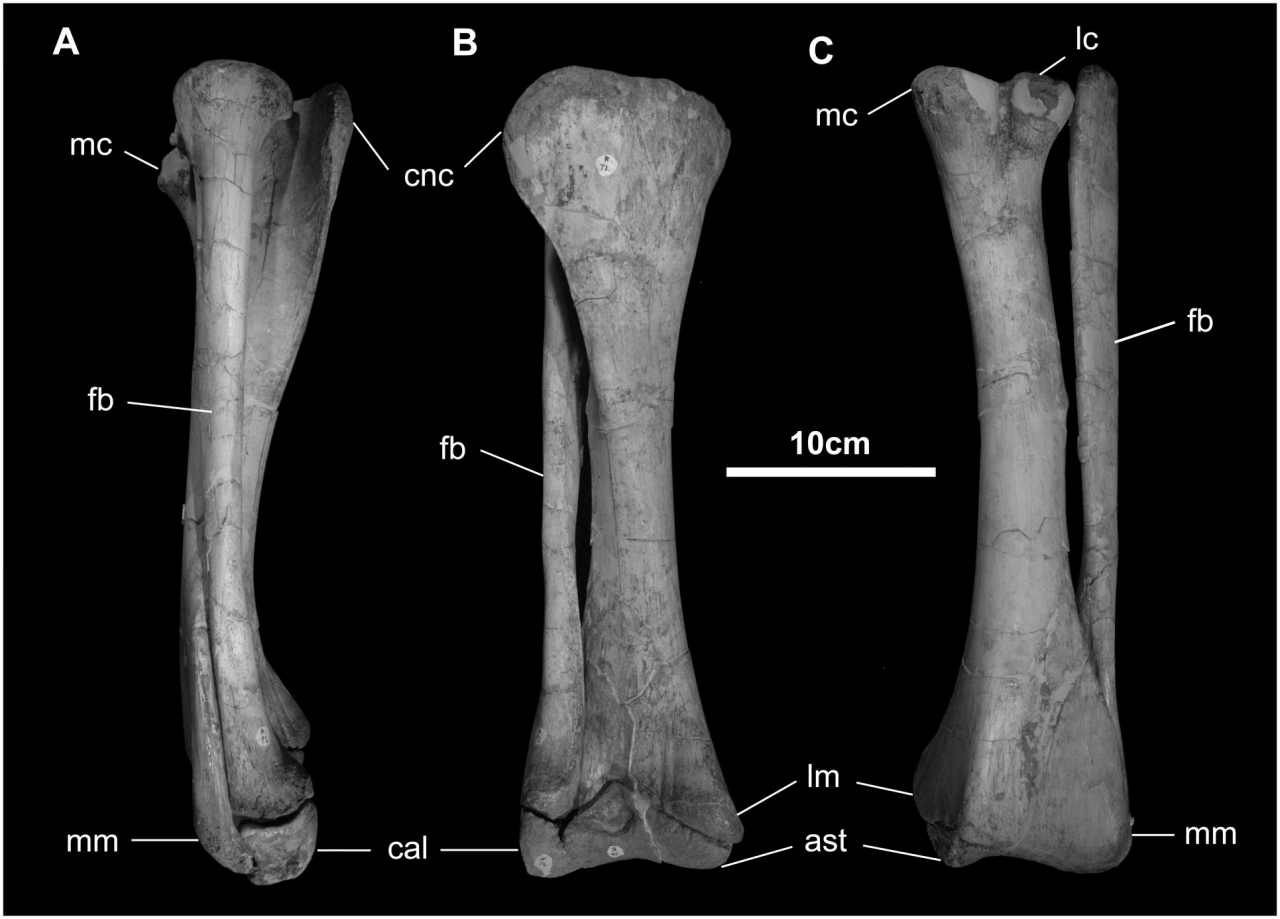
Tibia and fibula of *Gobihadros mongoliensis*. Tibia and fibula (MPC-D100/746, right) in lateral (A), anterior (B), and posterior (C) views. Abbreviations: ast, astragalus; cal, calcaneum; cnc, cnemial crest; fb, fibula; lm, lateral malleolus; lc, lateral condyle; mc, medial condyle; mm, medial malleolus.

*Tibia* (Fig 30): The tibia is typically sigmoid in lateral view and twisted along its long axis. Proximally, the well-developed cnemial crest projects forward and curves laterally to form a fossa for the head of the fibula; it extends nearly to the midshaft, as in hadrosaurids. The platform for the medial condyle of the femur is broad and contributes to the base of the cnemial crest. The proximal tibia also contributes to the platform for the lateral femoral condyle. The medial malleolus extends farther distally than the lateral aspect of the distal tibia, which accommodates the distal end of the fibula on its cranial surface. The end of the tibia is capped by the astragalus, which has a symmetrical ascending process.

*Fibula* (Fig 30): The fibula is long and narrow. Proximally it is enlarged to form a distinct head that fits into the fibular fossa of the proximal tibia. The interosseous ridge continues down the medial and caudal surface of the fibular shaft. On its distal end, the fibula again enlarges as it articulates with the lateral tibia and the calcaneum.

**Fig 31:**
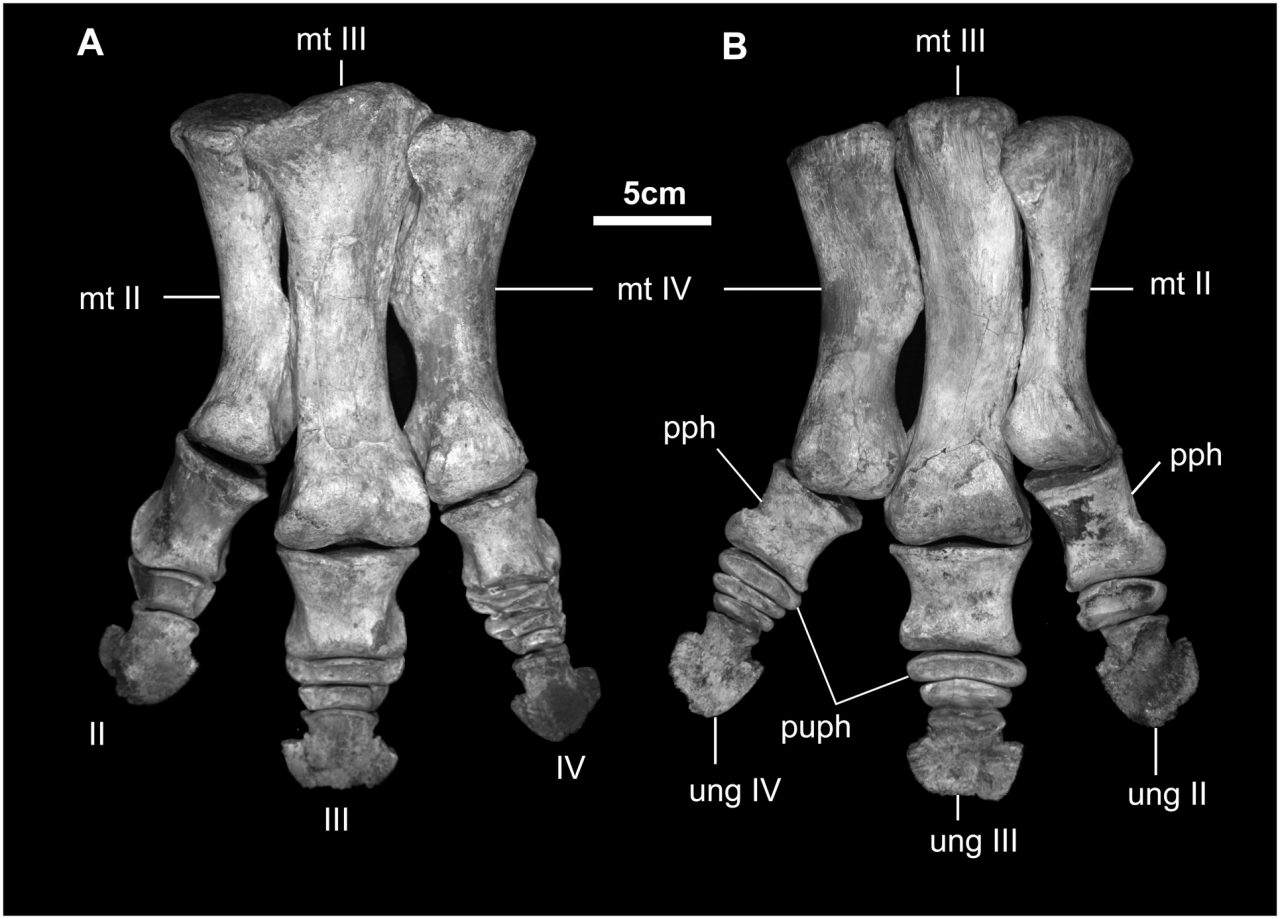
Pes of *Gobihadros mongoliensis*. Pes (MPC-D100/754, left) in dorsal (A) and ventral (B) views. Abbreviations: II-IV, second through forth digits; mt-II, second metatarsal; mt-III, third metatarsal; mt-IV, fourth metatarsal; pph, proximal phalanx; puph, penultimate phalanx; ung, ungual.

*Pes* (Fig 31): The astragalus and calcaneum comprise the only preserved tarsal elements. The proximal aspect of the astragalus articulates with the tibia and forms a shallow trochlear surface distally. The calcaneum is represented only by a small nubbin of bone that articulates proximally with the fibula and tibia and medially with the astragalus. Distal tarsals, as well as Metatarsal I, are absent. The phalangeal formula is 0-3-4-5-0 (based on MPC-D100/754), as in all hadrosauroids [52] [7, 45, 46, 51]. The penultimate phalanges of the digits II through IV are axially shortened to disc-like elements at least three times as wide as long. The unguals are dorsoventrally flattened and broadened to form a hoof-like morphology, as in hadrosaurids, *Tethyshadros*, *Tanius*, *Bactrosaurus*, and *Nanyangosaurus* [17, 18, 51, 52, 54, 73].

## Phylogenetic analysis

In order to assess the systematic position of *Gobihadros mongoliensis* and its relationships to other hadrosauroids, a phylogenetic analysis was conducted based on the recent analysis of Wu & Godefroit [11]. We opted to use this matrix, rather than others that are also available, because it was designed to assess the relationships of non-hadrosaurid hadrosauroids, rather than sample the large diversity in hadrosaurids, thereby reducing character redundancy and an unneccessary number of taxa in the analysis. *Gobihadros mongoliensis* definitely lacks many of the key features of hadrosaurids (e.g., more than 32 rows of teeth; miniaturized, lancoelate teeth; enlarged deltopectoral crest of humerus, etc), and is clearly not a member of Saurolophidae (= Euhadrosauria of [67], the least inclusive clade containing *Edmontosaurus* and *Parasaurolophus*). In addition to *Gobihadros*, nine other taxa were added to the matrix in order to increase taxon sampling of non-saurolophid hadrosauroids. The following taxa were scored for the characters in Wu & Godefroit [11] based on a combination of first-hand observation of some of the specimens, and the published literature: *Eotrachodon orientalis* [29], *Hadrosaurus foulkii* [27, 74], *Claosaurus agilis* [27, 69], *Jeyawati rugoculus* [56], *Plesiohadros djadokhtaensis* [24], *Xuwulong yueluni* [22], *Lophorhothon atopus* [68], *Gilmoreosaurus mongoliensis* [57]. The putative hadrosauroid *Koshisaurus katsuyama* was also added to the matrix, and scored on the basis of its original description [75]. The total matrix had 36 taxa and 108 characters (S1 Appendix). We recognize that this is a relatively small-scale analysis compared to other iguanodontian phylogenetic matrices and their iterations that have been recently developed and published (e.g., [9, 11–13, 18, 23, 26–29]). This analysis focuses on non-hadrosaurid hadrosauroid variation, and is intended to present a preliminary hypothesis that we expect will be further tested and refined as the new anatomical data presented in this paper makes its way into the matrices of other ornithopod researchers. A large-scale analysis of Iguanodontia that includes *Gobihadros mongoliensis* and further refines its systematic position is currently in the process of being published (Godefroit and Evans, The Dinosauria, 3^rd^ Edition, Cambridge University Press).

The data matrix was compiled in Mesquite v3.02 [76] and analyzed using the Branch-and-Bound algorithm in PAUP v4.0127 [77], with 14 characters designated as ordered following the original analysis of Wu & Godefroit (2012), and *Hypsilophodon foxii* designated as the outgroup. In order to assess the robustness of the topological results, a Bootstrap analysis (10,000 replicates) was conducted in PAUP using heuristic search criteria (10,000 replicates, random addition sequences, TBR branch swapping holding ten trees per replicate), and Bremer Decay values were calculated manually.

**Fig 32:**
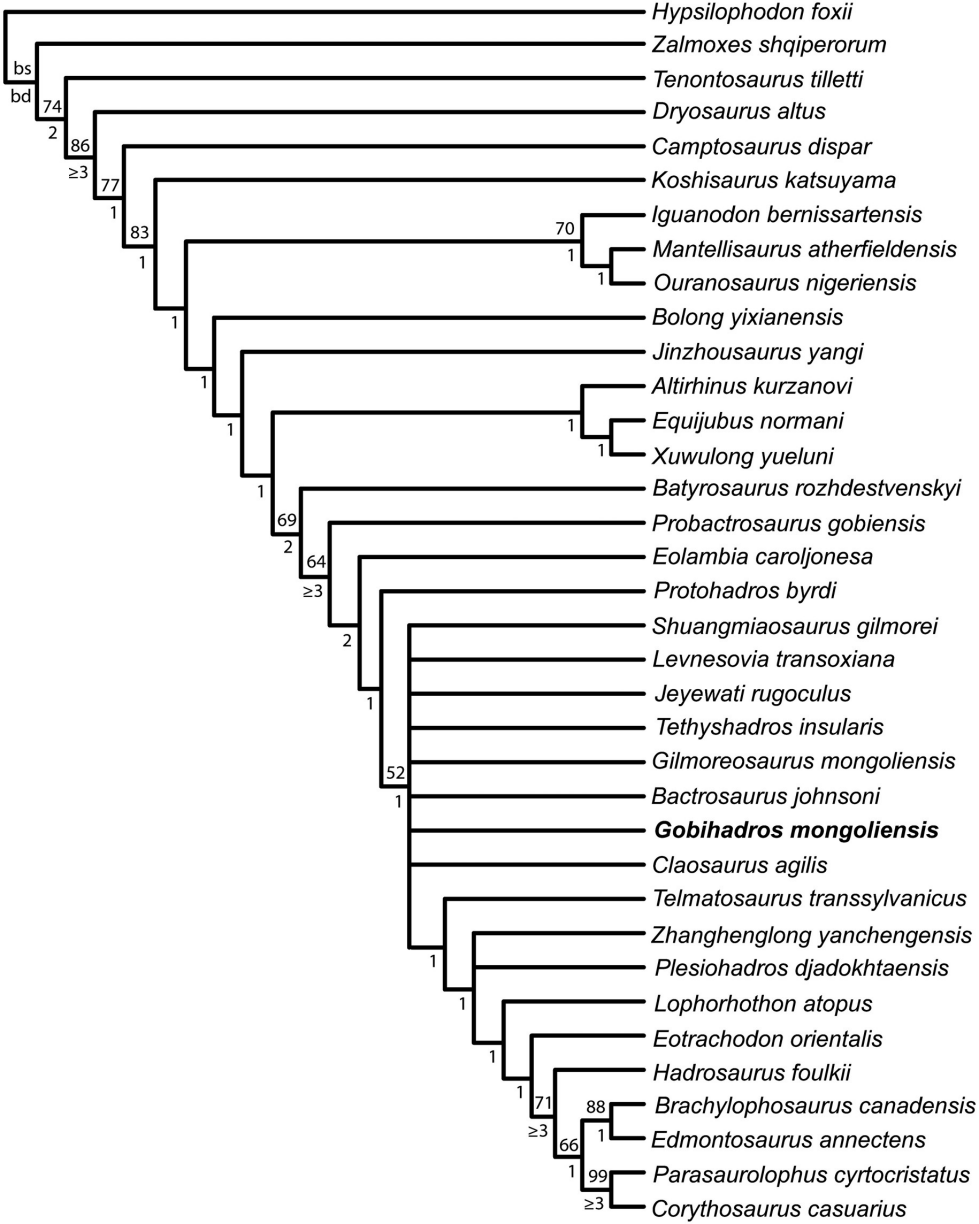
Cladogram depicting the phylogenetic relationships of *Gobihadros mongoliensis*. Strict Consensus Tree of 208 most parsimonious trees resulting from the phylogenetic analysis (see text for details), indicating the phylogenetic position of *Gobihadros mongoliensis* amoung hadrosauroids. Tree Length = 226 steps, consistency index = 0.59, retention index = 0.85.

The analysis resulted in 208 most parsimonious trees (MPTs), each with a tree length of 226 steps, consistency index is 0.59, and retention index is 0.85. In the strict consensus tree (Fig 32), *Gobihadros mongoliensis*, is recovered as a hadrosauromorph (sensu Norman, 2014) in a large polytomy with *Bactrosaurus johnsoni*, *Gilmoreosaurus mongoliensis*, *Levnesovia transoxonia*, *Tethyshadros*, *Shuangmiaosaurus gilmorei*, *Jeyawati*, *Claosaurus*, and a large, generally resolved clade that includes *Telmatosaurus*, *Zhanghenglong*, *Plesiohadros*, *Lophorhothon*, and *Eotrachodon* as successive sister-taxa to Hadrosauridae. *Gobihadros* and these taxa, in general, share the derived absence of a jugal-ectopterygoid contact (character 34[2]) and loss of a surangular foramen (57[1]), the development of a free ventral flange on the jugal (36[1]), rugose, angular-sided tooth roots (61[2]) and high aspect ratio maxillary tooth crowns (63[1]).

The least inclusive clade containing *Telmatosaurus* and Hadrosauridae is diagnosed by at least five synapomorphies, including a maxillary foramen on the rostrolateral surface of the bone (30[1]), loss of a paraquadratic foramen (39[1]), caudal extension of the toothrow beyond the apex of the coronoid process (51[1]), three or more functional teeth in a dentary tooth family (56[2]), and a coracoid with a well-developed cranioventral process (80[1]). *Plesiohadros djadoktaensis* is recovered within this clade, in a polytomy with *Zhanghenglong yangchengensis* [23], and a clade consisting of *Lophorhothon*, *Eotrachodon*, and Hadrosauridae. *Plesiohadros* shares with these more derived members of the clade a proportionately wide predentary (43[1]), a tooth row that is parallel to the lateral side of the dentary (52[2]), angular restricted to the medial side of the lower jaw (59[1]), loss of manus digit one (87[2]), presence of a premaxilla with a reflected rim (5[1]) and a ‘double-layered’ oral margin (3[1]).

Interestingly, the recently described *Eotrachodon* [29] is the sister taxon to Hadrosauridae, and not a hadrosaurid proper in this analysis. Hadrosauridae is diagnosed by at least three synapomorphies: a well-developed, horizontal ectopterygoid ridge on the maxilla (31[1]), symmetrical dentary tooth crowns (68[1]), and the absence of secondary ridges on the dentary teeth (69[1]). Additionally, *Koshisaurus katsuama*, posited as a potential hadrosauroid by Shiabata & Azuma [78], is the sister taxon of Hadrosauriformes and is not recovered as a hadrosauroid in this analysis.

The relatively low consistency index indicates a high degree of homoplasy in the data, as found in other analyses [26, 29], and is consistent with the high number of MPTs and the generally poor resolution in the strict consensus tree.

## Discussion

Non-hadrosaurid hadrosauroids from the Late Cretaceous are relatively rare, but are significant for understanding the sequence of character acquisition in the origin of Hadrosauridae and their historical biogeography. Late Cretaceous non-hadrosaurid hadrosauroids have been collected throughout Laurasia, and include *Lophorhothon atopus* and *Eotrachodon orientalis* from the Santonian-early Campanian Mooreville Chalk Formation [29], *Claosaurus agilis* from the ?Campanian Niobrara Formation of Kansas [69], *Jeyawati rugoculus* from Moreno Hill Formation (Turonian) of New Mexico, USA [56], and *Telmatosaurus transsylvanicus* and *Tethyshadros insularius* from the latest Campanian-Maastrichtian European archipelago [17, 67]. However, the greatest diversity of taxa is from Asia, including *Bactrosaurus johnsoni* and *Gilmoreosaurus mongoliensis* from the ?early Maastrichtian Iren Dabasu Formation of Inner Mongolia [52, 57, 58], *Tanius sinensis* from the Campanian Jingangkou Formation of China [54], *Levnesovia transoxiana* from the Turonian Bissetky Formation of Uzbekistan [18], and *Plesiohadros djadokhtaensis* from the late Campanian Djadokhta Formation of Mongolia [24].

**Fig 33:**
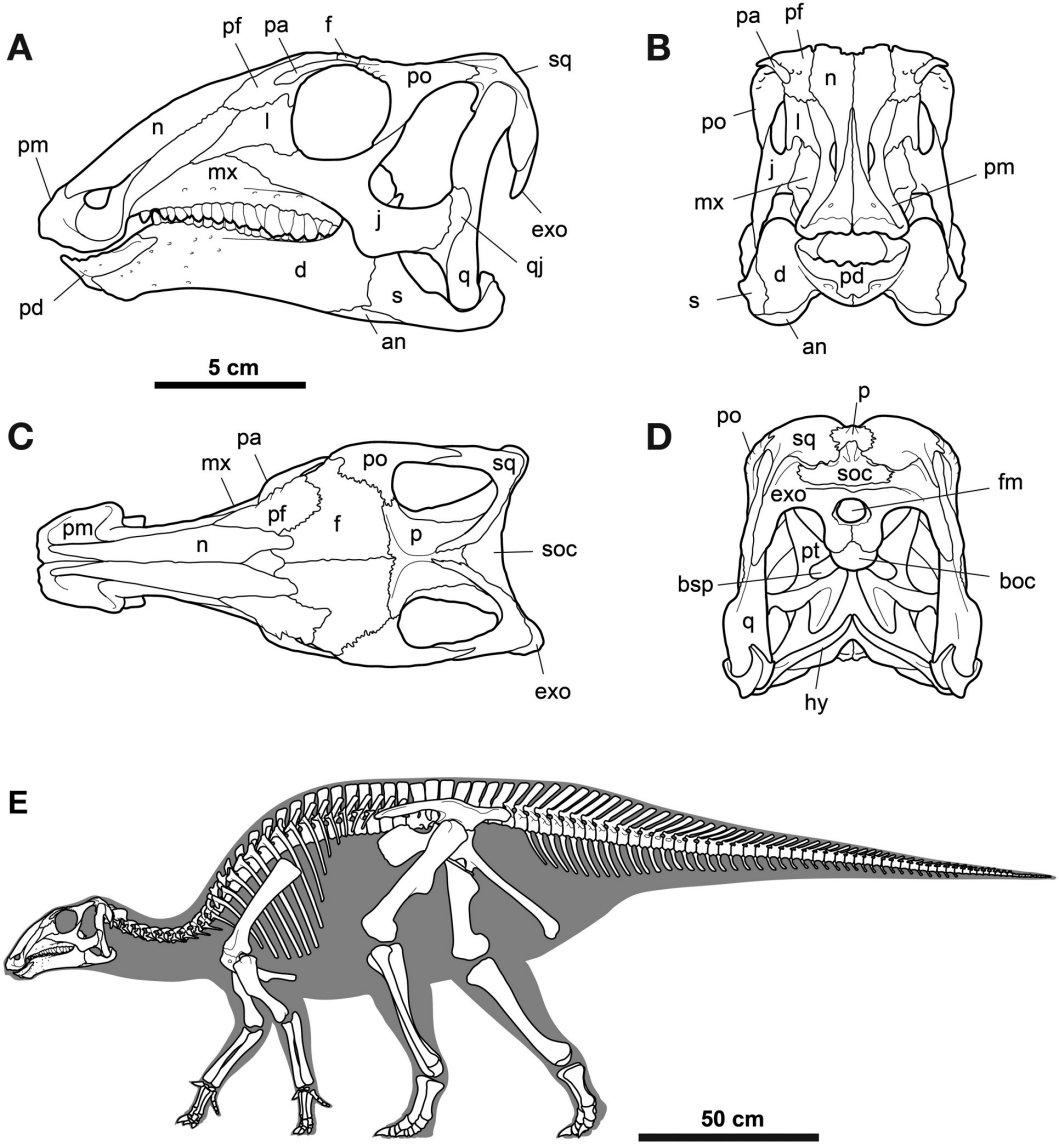
Skeletal reconstructions of *Gobihadros mongoliensis*. Skull (MPC-D100/763) of *Gobihadros mongoliensis* in left lateral (A), anterior (B), dorsal (C), and posterior (D) views. Schematic reconstruction of the skeleton of *Gobihadros mongoliensis* (E) in lateral view. Abbreviations: an, angular; boc, basioccipital; bsp, basisphenoid; d, dentary; exo, exoccipital; f, frontal; fm, foramen magnum; hy, hyoid; j, jugal; l, lacrimal; mx, maxilla; n, nasal; p, parietal; pa, palpebral; pd, predentary; pf, prefrontal; pm, premaxilla; po, postorbital; pt, pterygoid; q, quadrate; qj, quadratojugal; s, surangular; soc, supraoccipital; sq, squamosal.

**Fig 34:**
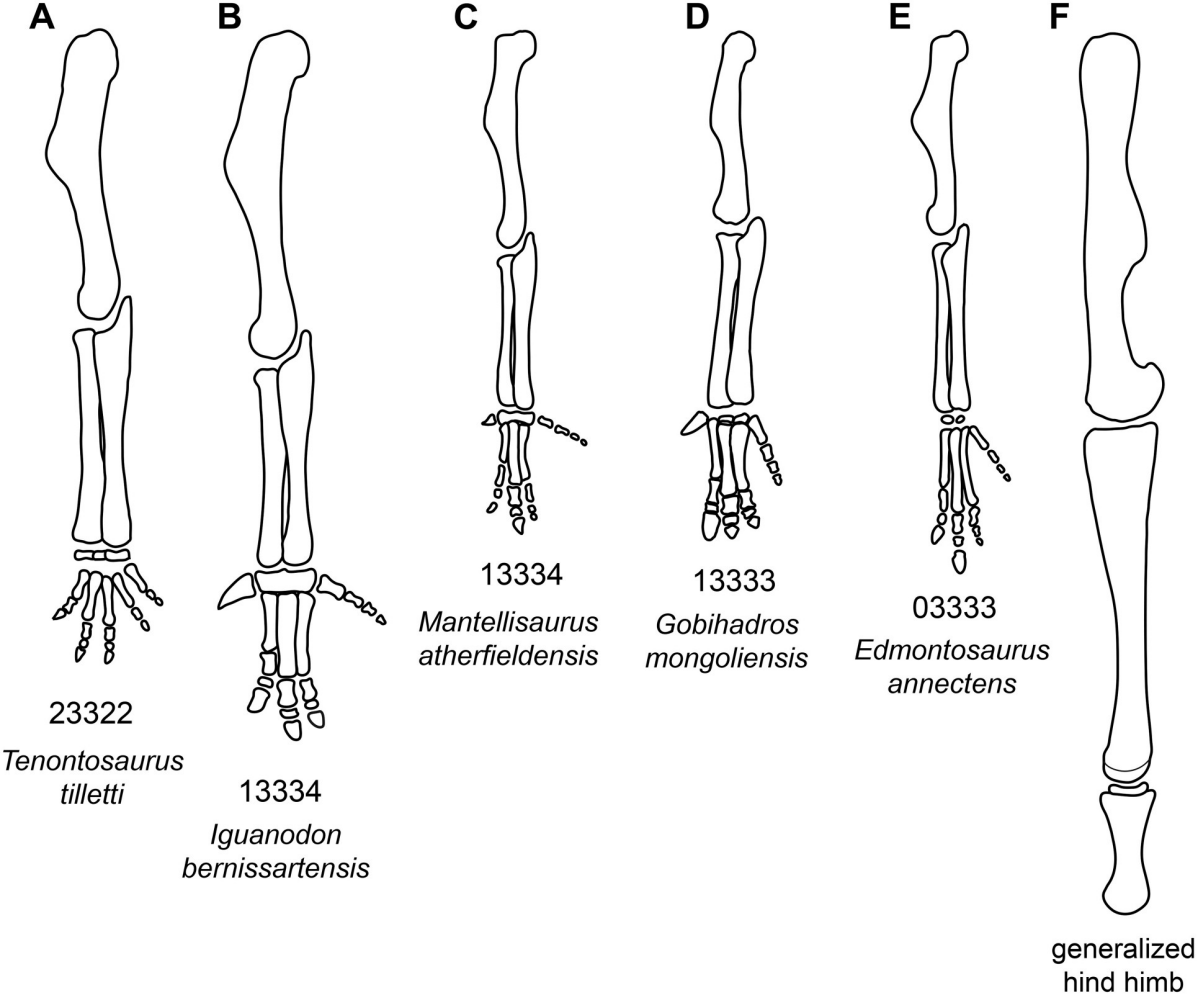
Schematic depicting the limb proportions of *Gobihadros mongoliensis* relative to other iguanodontians. The forelimbs of several iguanodontians on the left scaled to a generalized hindlimb on the right, following [44], to show differences in limb proportions. The phalangeal formula of the manus occurs below each forelimb of the corresponding taxon. Modified from Figure 81 of [44].

*Gobihadros mongoliensis* is the first non-hadrosaurid hadrosauroid from the Late Cretaceous of central Asia known from a complete, articulated skull and skeleton, and due to the exquisite nature of the preservation, it represents one of the most detailed records of their anatomy during the evolutionary transition to hadrosaurids (Fig 33). Most Asian hadrosauroid taxa are known from disarticulated bonebed material (e.g, *Gilmoreosaurus mongoliensis*, *Bactrosaurus johnsoni*) or from very fragmentary specimens (e.g., *Zhanghenglong yangchengensis*, *Claosaurus agilis*). As such, *Gobihadros* is an important taxon for evolutionary studies of hadrosauroids. The limb proportions of *Gobihadros*, with relatively short forelimbs compared to the total length of the hindlimbs, are more similar to hadrosauroids than to iguanodonts such as *Iguanodon* and *Ouranosaurus* (Fig 34; [44]). *Gobihadros* also shows that some traits characteristic of iguanodontians from the Early Cretaceous, but absent in derived members of the group, persisted into the Late Cretaceous, including the presence of a conical digit one ‘thumb-spike’ and a small, anteriorly-positioned external naris. Based on our phylogenetic analysis, *Gobihadros mongoliensis* is part of a plexus of closely-related ‘*Bactrosaurus*-grade’ taxa that includes *Bactrosaurus* and *Gilmoreosaurus* from the Iren Dabasu Formation (?latest Campanian-early Maasstrichtian, inner Mongolia), *Levnesovia* (Turonian of Uzbekistan,), *Shuangmiaosaurus* (Albian of China), *Tethyshadros* from Europe, and *Jeyawati* and *Claosaurus* from the early Late Cretaceous of North America. These taxa, including *Gobihadros*, form a polytomy with a clade that includes *Telmatosaurus*, *Zhanghenglong*, *Plesiohadros*, *Lophorhothon*, and *Eotrachodon* as successive sister-taxa to Hadrosauridae.

*Gobihadros mongoliensis* is from the same general area as *Bactrosaurus johnsoni* and *Gilmoreosaurus mongoliensis*, both of which are from the Iren Dabasu Formation of Inner Mongolia, northern China. The strata of the Baynshire Formation had once been considered very close in age to those of Iren Dabusu [39, 79], however more recent work has suggested that the Iren Dabasu Formation is latest Campanian to early Maastrichtian in age [80], and therefore considerably younger than the pre-Campanian Late Cretaceous age of the Baynshire Formation [40], although the young age of the Iren Dabasu has recently been questioned [18, 81]. Although *Gobihadros* shares substantial morphological similarities with *Bactrosaurus johnsoni* and *Gilmoreosaurus mongoliensis*, it can be easily distinguished from them in several autapomorphic traits, including the maximum number of functional dentary teeth that contribute to the triturating surface (three in *Gobihadros*, one or two in the Iren Dabasu taxa), and a premaxillary oral margin with the ‘double-layer morphology’. *Gobihadros* can be further distinguished in the sigmoidal dorsal outline of the ilium with a well developed, fan-shaped posterior process. All of these characters in *Gobihadros* are inferred to be convergent in Hadrosauridae in this analysis.

Hadrosauromorphs (sensu Norman[82]) were likely widely distributed across Laurasia in the middle Cretaceous, and the generally poor resolution of this phylogenetic analysis makes biogeographic inferences difficult. Centers of origin and faunal interchange in other dinosaurs between the Asia and North America may also prove to be more complex than previously considered (e.g., [83–85]), and these types of inferences, whether between Asia and North America or involving other landmasses, clearly benefit from a phylogenetic perspective (e.g., [30, 86–90]). However, the most proximate sister taxa to Hadrosauridae are from North America, as are *Hadrosaurus* itself (and basal saurolophines) in this phylogenetic analysis. Therefore, this analysis supports several recent biogeographic analyses that suggest hadrosaurids originated in North America [29, 30]. However, these and other analyses suggest that lambeosaurines were likely to have originated in Asia [29, 30, 91].

*Gobihadros mongoliensis* also fills in a critical gap in the Late Cretaceous hadrosauroid record of Mongolia, and reinforces a major faunal turnover event in hadrosauroids at the beginning the Nemegtian time period [24]. The latest Campanian to Maastrichtian-aged deposits of the Nemegt Formation preserve abundant remains of the hadrosaurids *Saurolophus* and *Barsboldia* [92–95], but no non-hadrosaurid hadrosauroid remains are currently known from this unit, despite the presence of *Bactrosaurus johnsoni* and *Gilmoreosaurus mongoliensis* in the potentially time-equivalent beds of the Iren Dabasu Formation in Inner Mongolia [80], but see [81]. In contrast, Hadrosauridae appear to be absent in the Gobi Desert of Mongolia prior to the Maastrichtian [24]. The recently described non-hadrosaurid hadrosauromorph *Plesiohadros djadokhtaensis* occurs in the Campanian-aged Djadokhta Formation [24], and now *Gobihadros mongoliensis* is known from the Cenomanian-Santonian Baynshire Formation. Hadrosaurids are well known from Santonian and Campanian-aged deposits of northern China [23, 96], as well as other localities in central Asia [97, 98]. Yet, in both Asia and North America, there are no known formational co-occurrences of non-hadrosaurid hadrosauroid species with hadrosaurids (e.g., [28]). In the absence of geographic barriers, these patterns suggest competitive exclusion of non-hadrosaurids by hadrosaurids and/or differences in palaeoenviromental/habitat preferences [24], but the geographic and temporal distribution of hadrosauroids needs considerable further research to illuminate the factors controlling their biogeography in the Late Cretaceous of Laurasia.

## Supporting information

S1 File Measurements of select hadrosauroid specimens from the Cretaceous of Mongolia. (PDF)

S1 Appendix Data matrix used in the phylogenetic analysis, based on Wu & Godefroit [11]. (DOCX)
