# Supplementary material for: A new hadrosauroid (Dinosauria: Ornithopoda) from the Late Cretaceous Baynshire Formation of the Gobi Desert (Mongolia)
Source: PLoS One. 2019 Apr 17;14(4):e0208480. doi: 10.1371/journal.pone.0208480 (PMC6469754; doi:10.1371/journal.pone.0208480)
Supplement: S1 File — (PDF) [file pone.0208480.s001.pdf]

# S1 File: Specimen Measurements

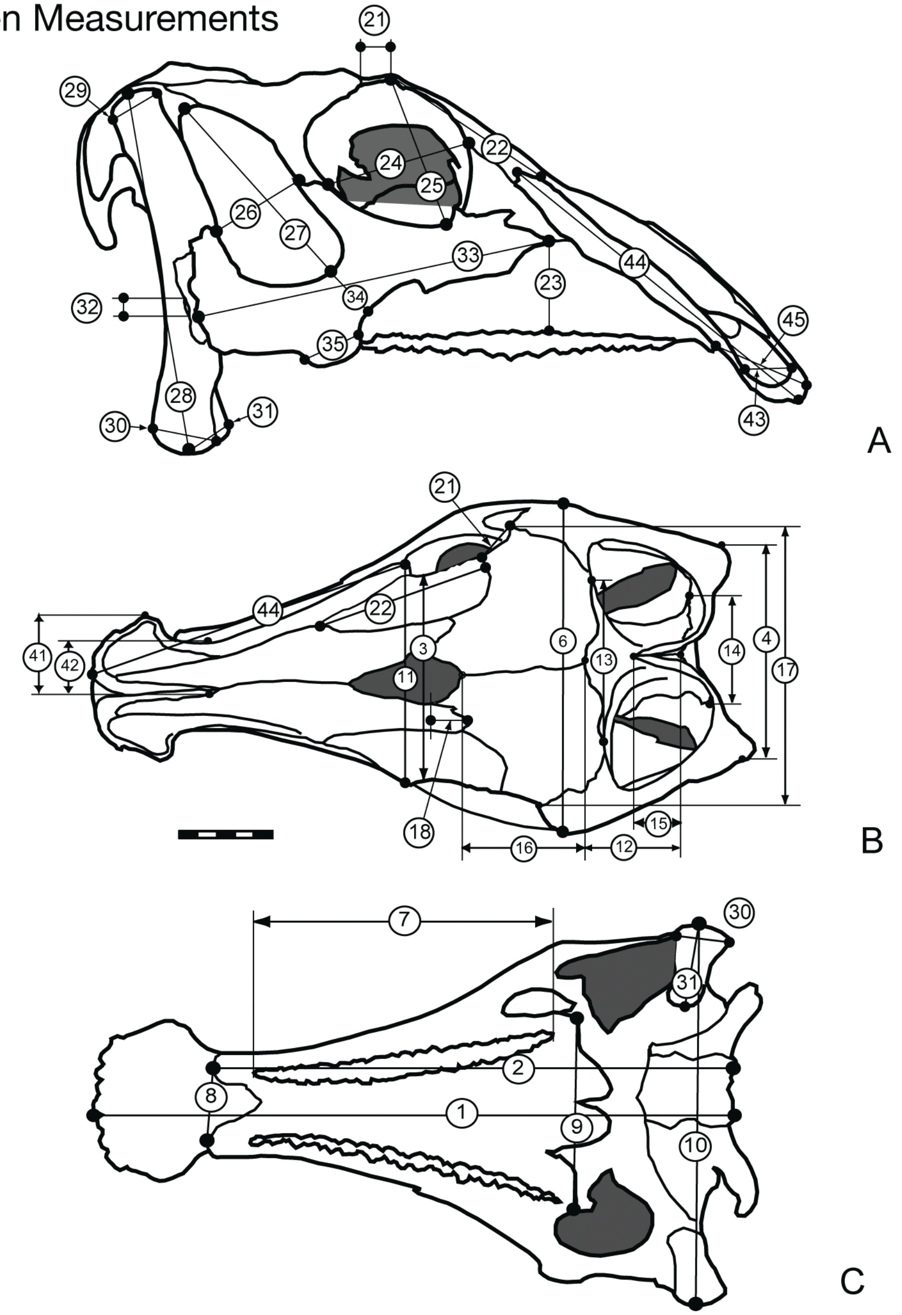


Measurement figure-1. Cranium. A- in right lateral view; B- in dorsal view; C- in ventral view;

Landmark explanation for measurements:
1- Length of skull, from base of occipital condyle to the anterior margin of premaxilla in palatial view; 2- Length of skull, from base of occipital condyle to the anterior end of rostroventral process of the maxilla; 3- Width of skull on orbital region; 4- Width of skull on occipital region; 6- Transverse width in postorbital region in dorsal view; 7- Length of maxillary tooth row; 8- Width between two anterior ends of maxilla; 9- Width between two posterior ends of maxilla; 10- Width between two mandibular condyle of quadrate (from lateral surface); 11- Width of skull on anterior margin of orbit at jugal and lacrimal suture; 12- Greatest length of parietal along midline; 13- Greatest width of parietal across anterior end; 14- Greatest width of parietal across posterior end; 15- Parietal crest length; 16- Greatest length of frontal, dorsal surface on median line; 17- Greatest width of frontals; 21- Length of frontal at orbital margin; 22- Length of prefrontal; 23- Height of maxilla, from joint point of jugal, lacrimal and maxilla to the margin of alveolar through; 24- Length of orbit, from the suture of prefrontal and lacrimal to the joint point for jugalar process of postorbital and postorbital process of jugal; 25- Height of orbit, from the prefrontal-frontal suture to the lowest margin of the anterior part of jugal; 26- Length of infratemporal fenestra, from the suture of postorbital-jugalar processes to the dorsal joint point of quadrate and jugal; 27- Height of infratemporal fenestra, from the postorbital-squamosal suture to the lowest bottom of infratemporal fenestra; 28- Height of Quadrate; 29- Length of squamosal condyle of Quadrate; 30- Length of mandibular condyle of Quadrate; 31-Width of mandibular condyle of Quadrate; 32- Height of paraquadrate opening; 33- Length of Jugal, from the rostral pointed process to the posterior margin of quadratojugalar process; 34- Depth of Jugal at constriction below intratemporal fenestra; 35- Length of the free ventral flange on jugal; 41- Maximium width of premaxilla; 42- Minimium width of premaxilla; 43- Length of external nares; 44- Length of latero-ventral process of premaxilla; 45-Length of oral portion of premaxila, from oral margin to rostral process of maxilla

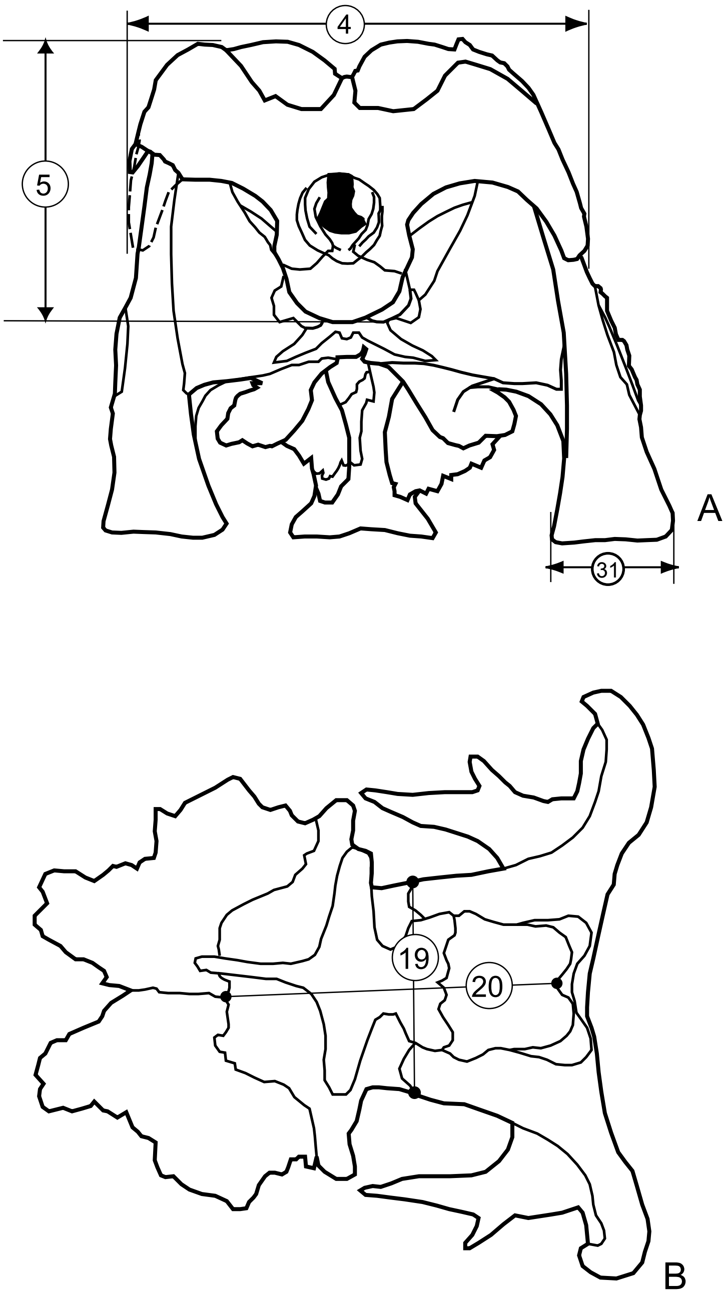


Measurement figure-2. Cranium. A- in posterior view; B- in ventral view;

4- Width of skull on occipital region; 5- Height of occiput; 19- Length of braincase, from the posterior end of the basioccipital condyle to the anterior margin of the presphenoid; 20- With of braincase, between left and right points of prootic and laterosphenoid suture on the top of foramina V cranial nerve; 31- Width of mandibular condyle of the quadrate;

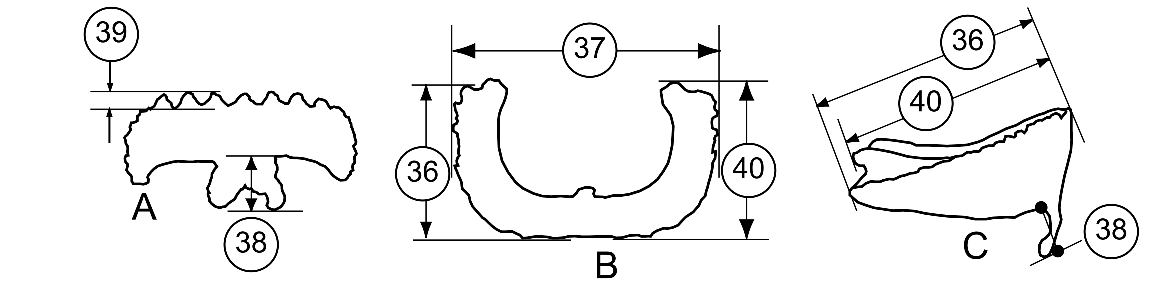


Measurement fig.3 Predentary. A- in anterior view; B- in dorsal view; C- in right lateral view;

Explanation for measurements:
36- Length form rostral margin to the posterior end of lateral process; 37- Greatest width; 38- Length of ventral process; 39- Height of denticle; 40- Length of dorsal process;

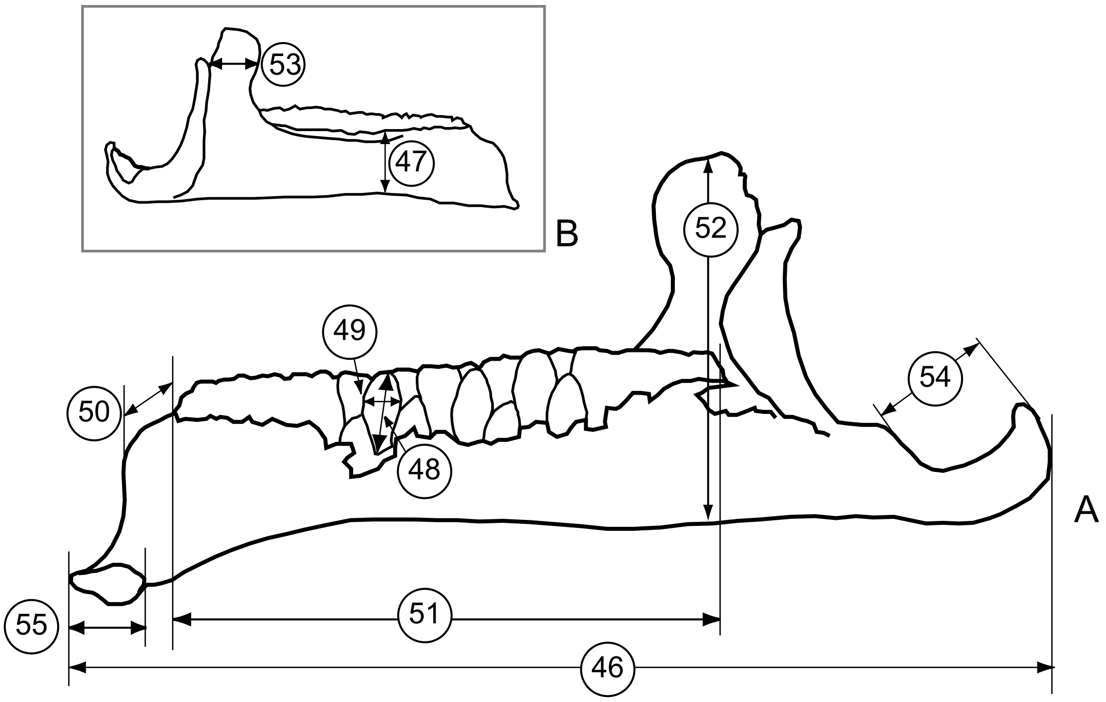


Measurement fig.4 Right mandible. A- in medial view; B- in lateral view

Explanation for measurements:
46- Greatest length, from the rostral end of predentary process to the posterior margin of articular;
47- Height of ramus at center of tooth row; 48- Tooth crown length at center of tooth row; 49- Tooth crown width at center of tooth row; 50- Length of diastema; 51- Length of tooth row; 52- Height of coronoid process; 53- Length of coronoid process; 54- Retroarticular length; 55- Rostral symphysis length;

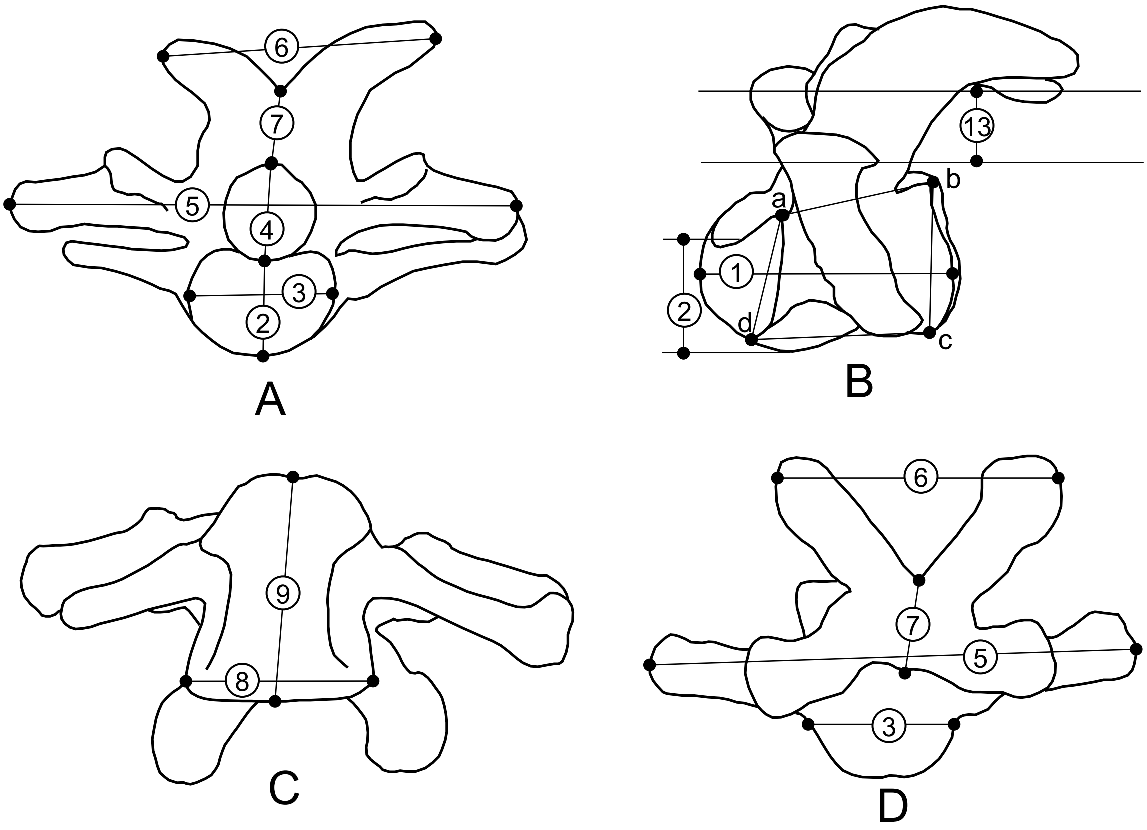


Measurement Fig.5 Cervical vertebra. A- in anterior view; B- in left lateral view; C- in ventral view; D- in dorsal view

Landmark explanation for measurements:
1- Length of the centrum, the longest axis of centrum; 2- Height of the centrum anteriorly, from the bottom of the neural canal to the ventral rim of the centrum; 3- Width of the centrum anteriorly, between lateral rims of the centrum in center; 4- Height of the neural canal anteriorly, from the roof of the neural canal to the bottom of the neural canal; 5- Length of transverse processes, between right and left lateral rims of transverse processes; 6- Width of postzygapophysis, between the lateral rims of right and left postzygapophysis; 7- Height of the neural spine; 8- Greatest width of centrum posteriorly; 9- Length of centrum ventrally; ab- Length of the centrum on dorsal margin, excluded anterior cotyle; bc- Height of the centrum posteriorly; dc- Length of the centrum in ventral margin, excluded anterior cotyle; ad- Height of the centrum anteriorly; 13- Height of the neural spine prependicularly along the anteroposterior axis of the vertebra, from the roof of the neural canal to the tip of the neural spine;

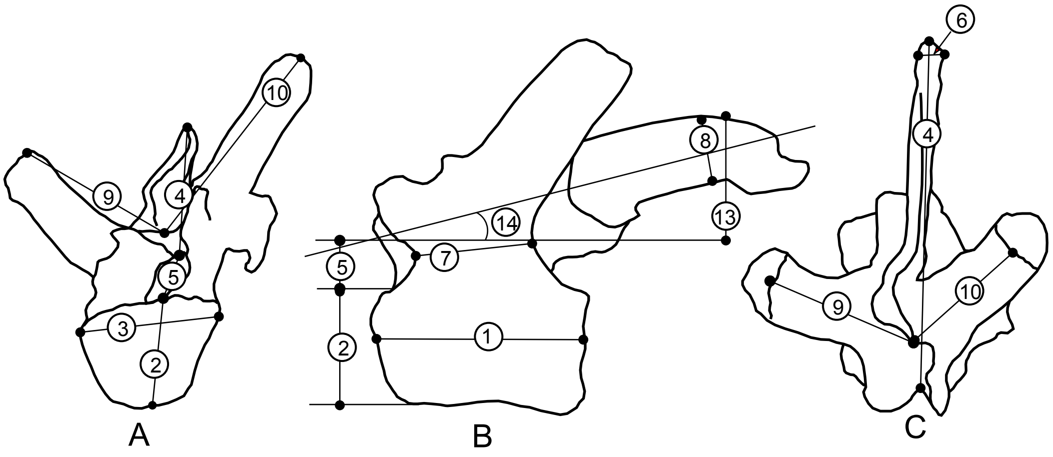


Measurement figure 6. Anterior dorsal vertebra. A- in anterior view; B- in left lateral view; C- in dorsal view;

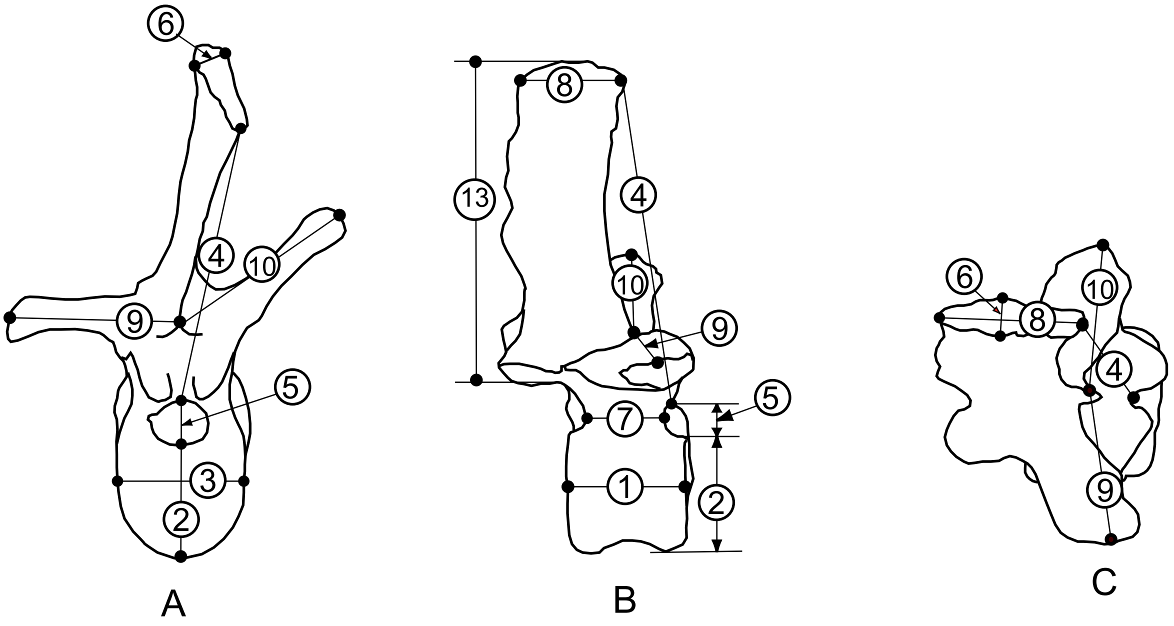


Measurement figure 7. Posterior dorsal vertebra. A- in anterior view; B- in right lateral view; C- in dorsal view;

Landmark explanation for measurement figure 6 and 7.

1- Length of centrum, the longest axis of centrum; 2- Height of centrum anteriorly, from the bottom of the neural canal to ventral rim of centrum; 3- Width of centrum anteriorly, between lateral rims of centrum in center; 4- Height of neural spine anteriorly, from the roof of the neural canal to the dorsal rim of the spine; 5- Height of neural canal anteriorly, from the roof of the neural canal to the bottom of the neural canal; 6- Width of the neural spine in dorsal end; 7- Length of the lateral wall of the neural canal; 8- Length of the neural spine in dorsal end; 9- Length of right transverse process, from the base of prespinal lamina to the center of lateral rim of transverse process; 10- Length of left transverse process, from the base of prespinal lamina to the center of lateral rim of transverse process; 13- Height of the neural spine prependicularly along the anteroposterior axis of the vertebra , from the roof of the neural canal to the tip of the neural spine; 14- Angle between the along of the neural canal roof and inclination of the neural spine

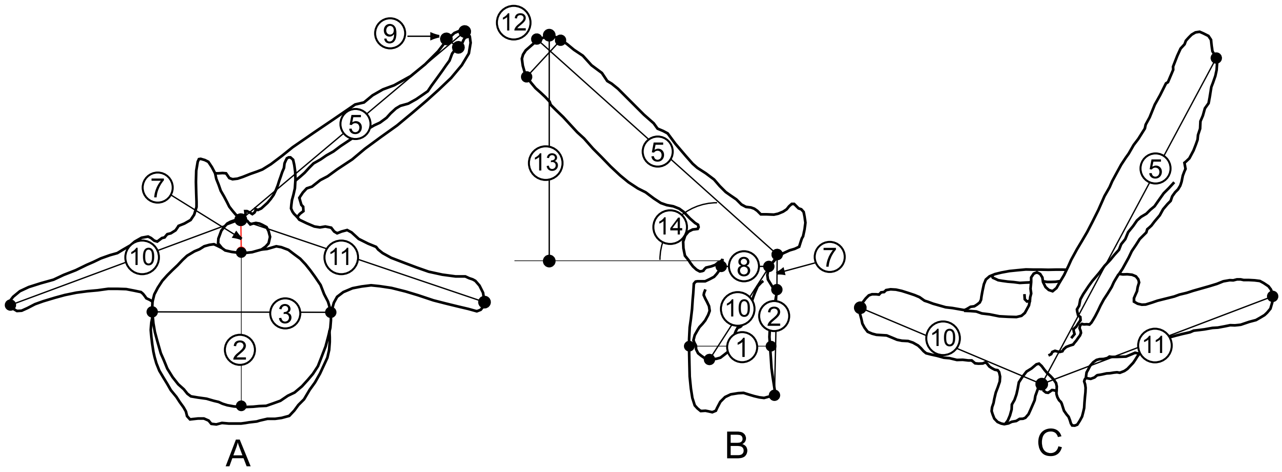


Measurement Figure 8. Anterior caudal vertebra. A- in anterior view; B- in right lateral view; C- in dorsal view

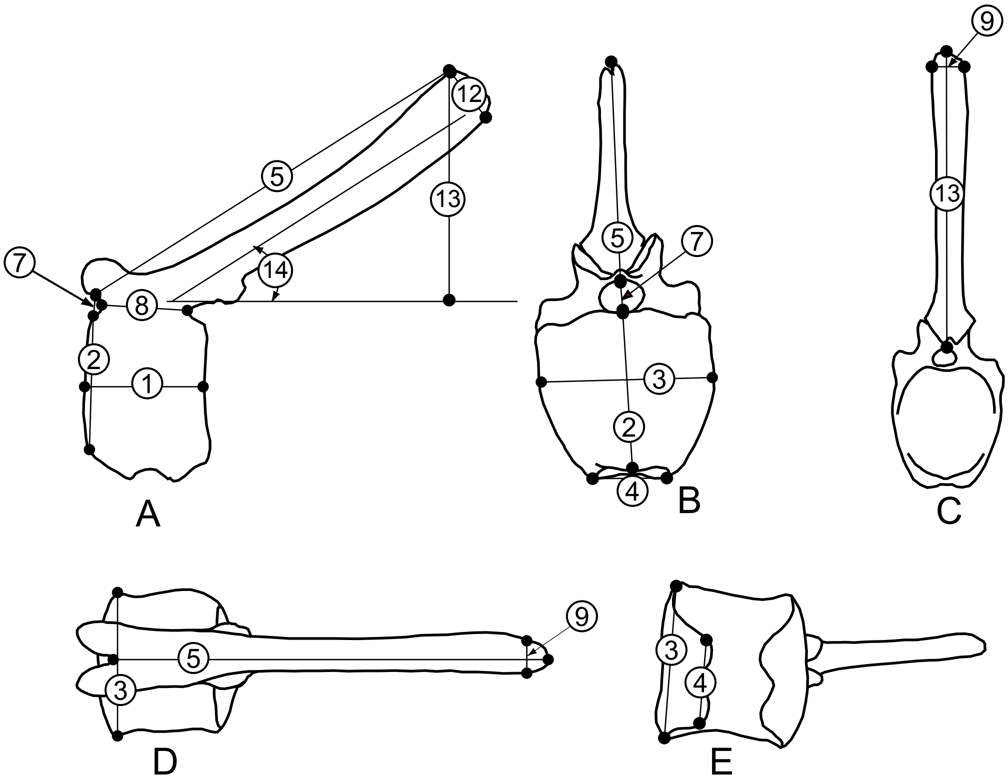


Measurement figure 9. Medial caudal vertebra. A- in lateral view(left); B- in anterior view; C- in posterior view; D- in dorsal view; E- in ventral view

Landmark explanation for measurement figure 8 and 9

1- Greatest length of centrum, the longest axis of the centrum; 2- Height of centrum in cranial articular surface, from the bottom of the neural canal to the center of articular facet of chevron; 3- Width of centrum in dorsal aspect anteriorly, between dorsolaterial margins of the centrum; 5- Height of neural spine, anteriorly, from the roof of the neural canal to the tip of the neural spine; 7- Height of neural canal in anterior aspect; 8- Length of base of the neural spine; 9- Width of dorsal rim of the neural spine; 10- Length of right transverse process, from the center of neural canal roof to the center of the lateral rim of the transverse process; 11- Length of left transverse process, from the center of neural canal roof to the center of the lateral rim of the transverse process; 12- Greatest length of the dorsal end of neural spine; 13- Height of the neural spine prependicularly along the anteroposterior axis of the vertebra, from the roof of the neural canal to the tip of the neural spine; 14- Angle between the along of the neural canal roof and inclination of the neural spine;

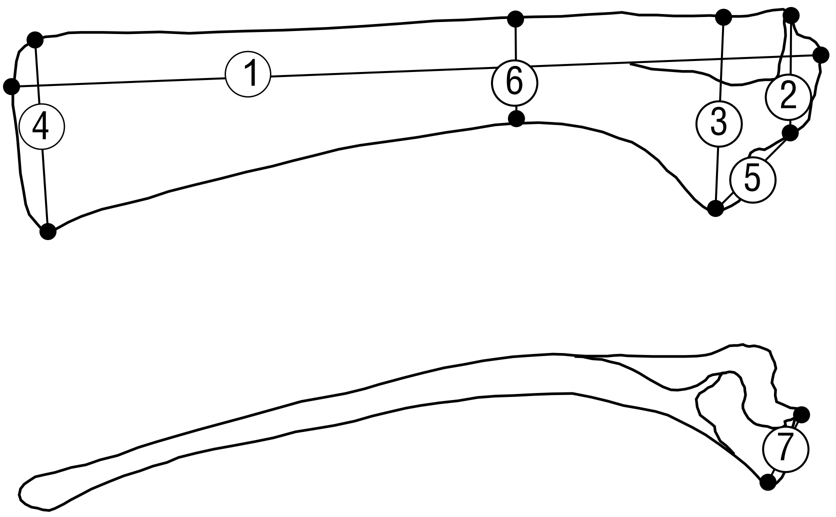


Measurement Figure 10. Scapula right. A- in lateral view; B- in ventral view;

Landmark explanation for measurements:
1- Maximum length, from dorsal point of coracoid articulation to maximum dorsal point of the distal scapular blade expansion; 2- Coracoid articulation width, from ventral point of coracoid articulation to dorsal point of coracoid articulation; 3- Maximum height through the glenoid fossa rim and the acromion process, from maximum dorsal point of the acromial process to maximum ventral point of glenoid fossa rim; 4- Maximum distal height, from maximum dorsal point of the distal scapular blade expansion to maximum ventral point of distal blade expansion; 5- Glenoid length, taken at 1/2 ventral width, from ventral point of coracoid articulation to maximum ventral point of glenoid fossa rim; 6- Minimum shaft height; 7- Mediolateral width at glenoid in ventral view from medial scapular articulation point to lateral scapular articulation point

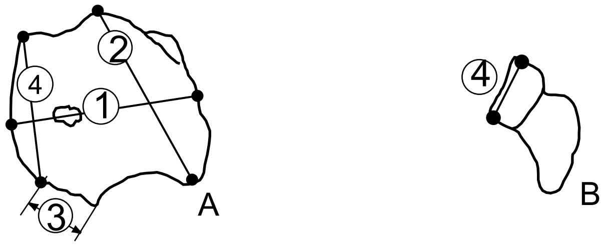


Measurement Figure 11. Coracoid right. A- in lateral view; B- in ventral view

Landmark explanation for measurement:
1- Maximum length, perpendicular to scapular articulation; 2- Maximum width, parallel to scapular articulation
3- Glenoid fossa length, from
5- Ventral scapular articulation width

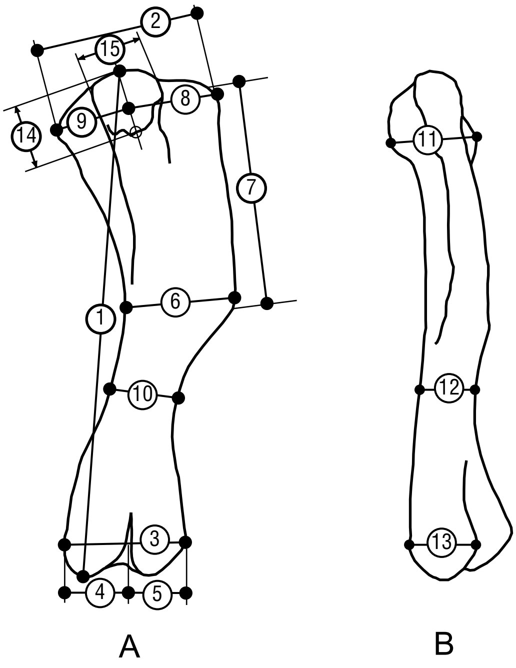


Measurement Figure 12. Right humerus. A- in caudal view; B- in lateral view;

Landmark explanation for measurement:
1- Maximum length, from the point on proximal border directly proximal to lateral border of head to the most distal point of medial condyle; 2-Maximum proximal width, from maximum lateral point of proximal articular surface to the most lateral point of proximal humerus; 3- Maximum distal width across condyles, perpendicular to long axis but rotated to maximum width, between maximum lateral point of lateral condyle and maximum medial point of medial condyle; 4- Medial condyle width, parallel to maximum distal width from maximum medial point of medial condyle to point in groove between ert- and epicondyles; 5- Lateral condyle width, parallel to maximum distal width, from point in groove between ert- and epicondyles to maximum lateral point of lateral condyle; 6- Width at the most lateral point of the distal deltopectoral crest from proximolateral corner of humerus to the distal limit of deltopectoral crest; 7- Deltopectoral crest length, from the external tuberosity to the distal end of the deltoid muscle scar; 8- Distance from the midpoint of the head to the lateral border; 9- Distance from the midpoint of the head to the medial border; 10- Minimum shaft width; 11- Craniolateral view, proximal width, from proximoventral corner of element to dorsal surface of humeral head; 12- Craniolateral view, minimum shaft width; 13- Craniolateral view, width of lateral condyle, positioned horizontally, from most dorsal point of lateral condyle to most ventral point of lateral condyle; 14- Head height; 15- Head width

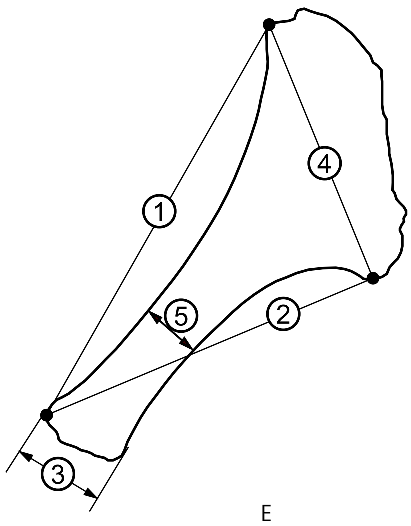


Measurement figure13. Left sternum in dorsal view.

Landmark explanation for measurement fig.13
1- Length, from the anterior edge of the distal end of "rod" to the anterior margin of the blade; 2- Length, from the anterior edge of the distal end of the rod to the posterior margin of the blade; 3- Width of the distal end of the rod; 4- Width of the blade; 5- Least width of the shaft;

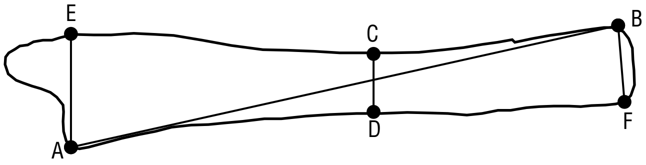


Measurement figure 14. Ulna right in medial view.

Landmark explanation for measurement fig.14
AB - Greatest length, from upper medial portion proximal end to upper lateral portion distal end;
CD - Minimium width of shaft; EA - Greatest width of the proximal end; FB - Greatest width of the distal end

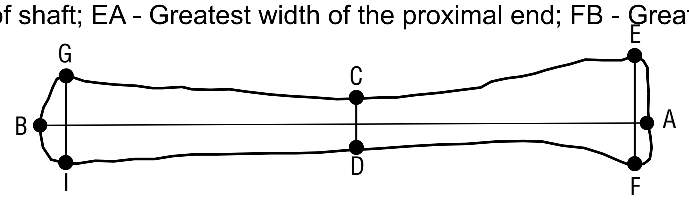


Measurement figure 15. Radius right in medial view.

Landmark explanation for measurement fig.16
AB - Greatest length; CD - Width of minimium of shaft; EF - Greatest width of the proximal end
GI - Greatest width of the distal end

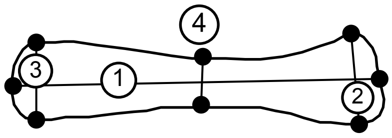


Measurement figure 16. Metacarpal III right in dorsal view.

1- Greatest length; 2- Width of the proximal end; 3- Width of the distal end; 4- Minimum width of shaft;

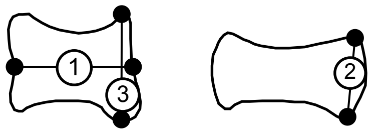


Measurement figure 17. Phalanx 1 of digit II of right manus. A- in lateral view; B- in dorsal view;

1- Length median line; 2- Width proximal end; 3- Height proximal end;

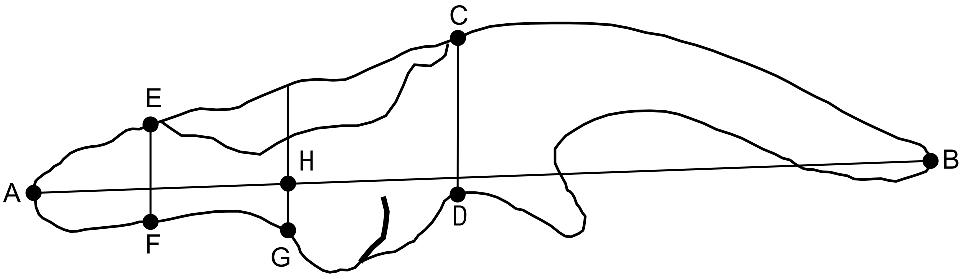


Measurement Figure 18. Ilium right in lateral view

Landmark explanation for measurements:
AB - Maximum length; CD - Height.The line is perpendicular to AB where D is on the asymptote of acetabulum. Point C may or may not fall on the anterior border of the antitrochanter;
HA - Length of postacetabular process. Point G lies on the posterior border of the ischial peduncle. Line HG is drawn perpendicular to line AB at this point. Line HB now defines the PPL; EF - Height of postacetabular process; BCD - angle preacetabular deflection

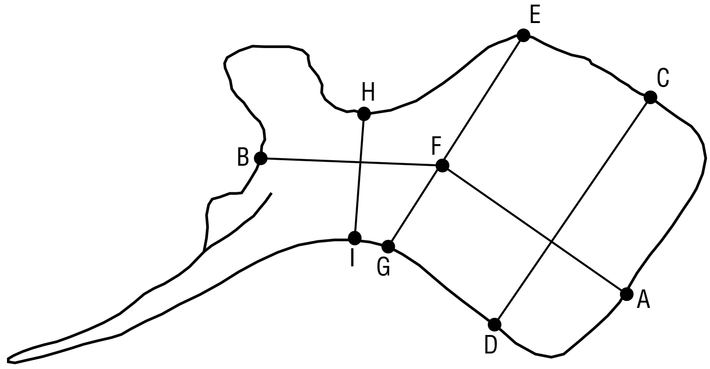


Measurement Figure 19. Pubis right in lateral view

Landmark explanation for measurements:
AB - Length, line AF bisects the blade into dorsal and ventral segments. Point B is on the asymptote of the acetabulum; CD -Height of the blade; AF - Blade length; IH - Minimum width of the neck

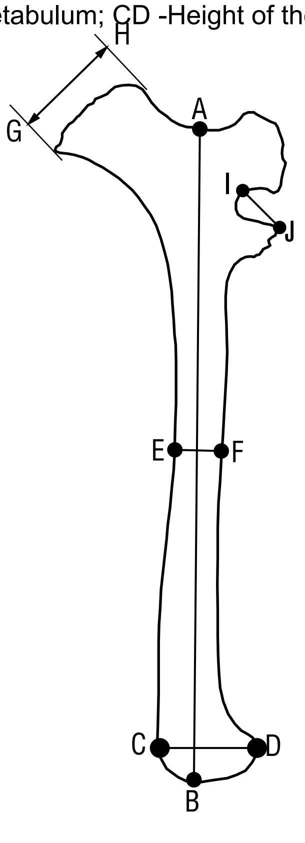


Measuremenr Figure 20. Ischium right in lateral view

Landmark explanation for measurements:
AB - Length, from the lowest point of the acetabular margin to the symptote of the ischial foot; CD - Width of the ischial foot; EF- Width of the shaft; GH - Length of iliac peduncle; IJ- Length of obturator process, from the lowest point of the obturator foramen to the proximal tip of the obturator process

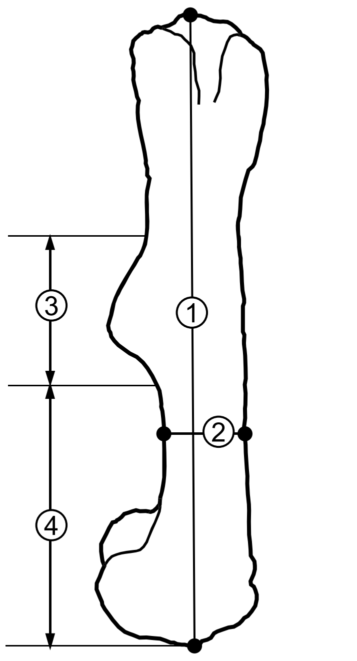


Measurement figure 21. Femur right in lateral view

Landmark explanation for measurements:
1- Greatest length; 2- Least width of midshaft; 3- Length of 4th trochanter; 4- Length of distal end, from the distal base of 4th trochanter to the distal end of lateral condyle

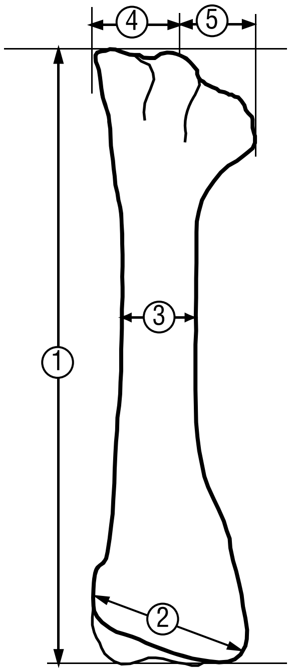


Measurement figure 22. Tibia right in caudal view

Landmark explanation for measurements:
1- Greatest length; 2- Width of the distal end; 3- Least width of midshaft; 4- Width of the caudal portion of the proximal end; 5- Width of the cnemial crest

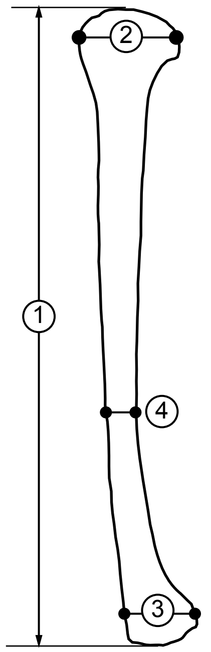


Measurement figure 23. Fibula right in lateral view

Landmark explanation for measurements:

1- Greatest length; 2- Greatest width of the proximal end; 3- Greatest width of the distal end; 4- Least width of the midshaft.

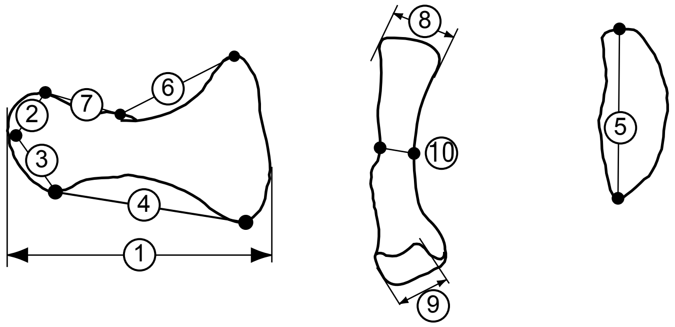


Measurement figure 24. Right metatarsal II. A- in medial view; B- in dorsal view; C- in posterior view;

Landmark explanation for measurements:

1- Greatest length; 2- Height of dorsal portion of the distal end; 3- Height of ventral portion of the distal end; 4- Ventral length, from heighest point of the proximal end to the caudal margin of the oblique articular surface in distal end; 5- Height of the proximal end;
6- Length, from the proximal end to the flange in dorsolateral surface; 7- Length, from the flange in dorsolateral surface to the caudal margin of the oblique articular surface in distal end; 8- Width of the proximal end; 9- Width of the distall end; 10- Width of the midshaft;

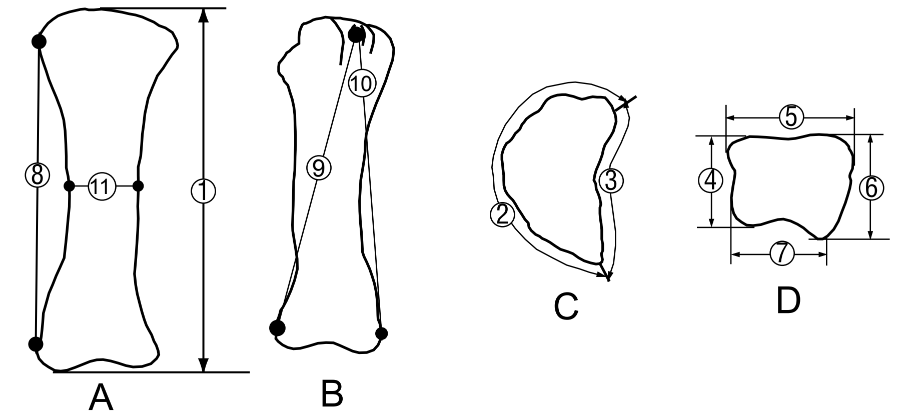


Measurement figure 25. Right metatarsal III. A- in dorsal view; B- in ventral view; C- in proximal view; D- in distal view;

Landmark explanation for measurements:
1- Greatest length; 2- Semicircle height of the proximal end in lateral view; 3- Height of the proximal end in medial view; 4- Height of the distal end in lateral view; 5- Width of the distal end in dorsal view; 6- Height of the distal end in medial view; 7- Width of the distal end in ventral view; 8- Length from the asymptote of proximal end in lateral view to the dorsolateral margin of the oblique articular surface in the distal condyle; 9- Length from the proximal end to the medial condyle in ventral view; 10- Length from the proximal end to the asymptote of the lateral condyle in ventral view; 11- Diameter of midshaft;

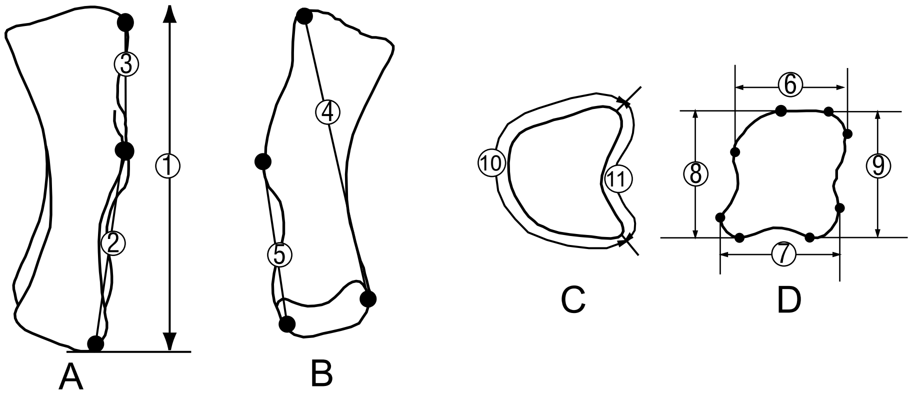


Measurement figure 26. Right metatarsal IV. A- in dorsal view; B- in ventral view; C- in proximal view; D- in distal view;

Landmark explanation for measurements:
1- Greatest length; 2- Length from the asymptote of distal end in medial view to the heighest point of the flange in dorsomedial surface; 3- Length from the heighest point of the flange in dorsomedial surface to the asymptote of the proximal end in dorsomedial view; 4- Length from the asymptote of the proximal end in ventro-medial surface to the ventro-lateral condyle; 5- Length from the heighest point of the flange in ventral view to the ventro-medial condyle; 6- Width of the distal end in ventral view; 7- Width of the distal end in lateral view; 8- Height of the distal end in lateral view; 9- Height of the distal end in medial view; 10- Length of proximal semicircle end; 11- Height of proximal end in medial view;

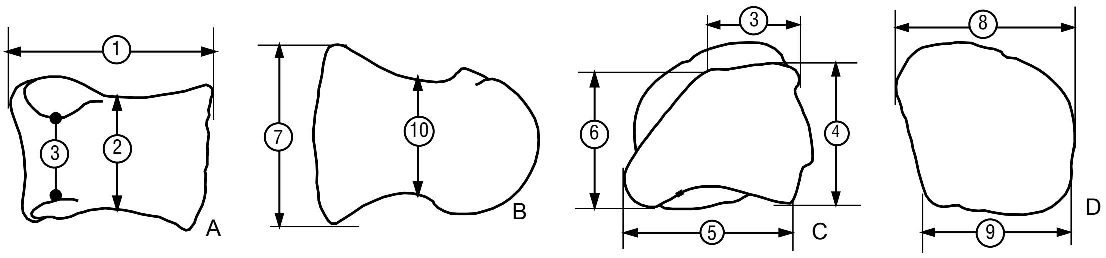


Measurement figure 27. Proximal phalanx of the left pedal digit II. A-in dorsal view; B-in medial view; C-in anterior view; D-in posterior view;

Landmark explanation for measurements:
1- Greatest length, 2- Least width of the midshaft, 3- Width of the distal end in dorsal view, 4- Height of the distal end in lateral view, 5- Width of the distal end in ventral view, 6- Hieght of the distal end in medial view 7- Height of the proximal end, 8- Width of the proximal end in dorsal view, 9- Width of the proximal end in ventral view, 10- Height of the midshaft

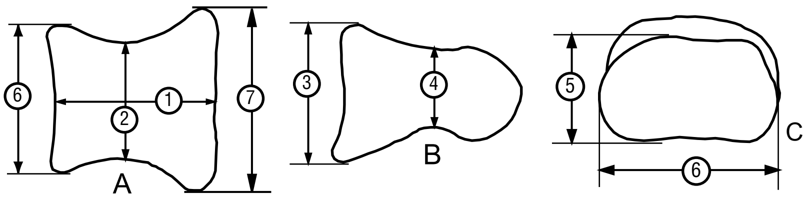


Measurement figure 28. Proximal phalanx of the left pedal digit III. A- in dorsal view; B- in medial view;C- in anterior view

Landmark explanation for measurements:
1- Greatest length, 2- Least width of the midshaft, 3- Height of the proximal end, 4- Height of midshaft 5- Height of the distal end, 6-Width of the distal end, 7- Width of the proximal end

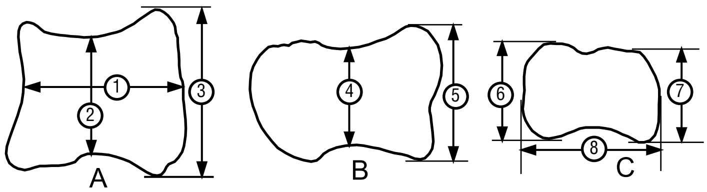


Measurement figure 29. Proximal phalanx of the left pedal digit IV. A- in dorsal view; B- in lateral view;C- in anterior view

Landmark explanation for measurements:
1- Greatest length; 2-Least width of the midshaft; 3-Width of the proximal end; 4- Height of the mishaft 5- Height of the proximal end; 6- Height of the distal end in medial view; 7- Height of the distal end in lateral view; 8- Width of distal end in ventral view

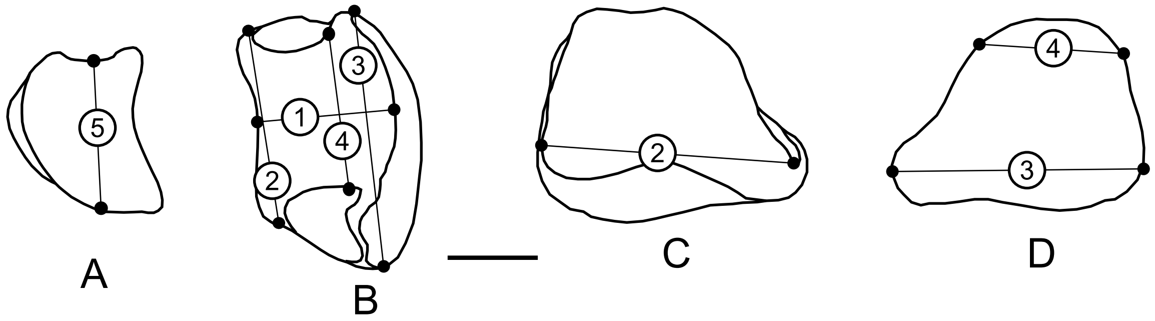


Measurement figure 30. Second phalanx of right digit II. A- in medial view; B- in dorsal view; C- in anterior view; D- in posterior view;

1- Length in median line; 2- Width of the distal end; 3- Width of the proximal end in ventral view; 4- Width of the proximal end in dorsal view; 5- Height of midshaft

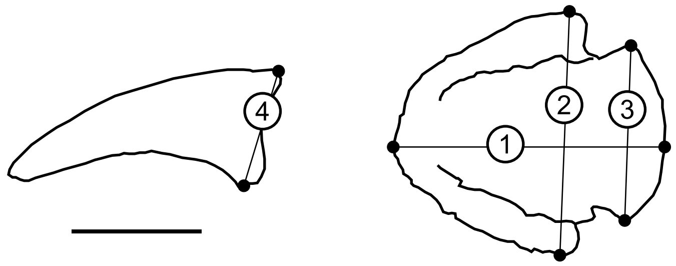


Measurement figure 31. Ungual phalanx of right digit III. A- in medial view; B- in dorsal view;

1- Length in median line; 2- Greatest width of hoof-shaped part of ungual; 3- Width of the proximal end; 4- Height of the proximal end

| Description of the measurement | MPC-D100/746 (BTs 95') | | MPC-D100/754 (KLT 04') | | MPC-D100/752 (BTs 01') | | Cast of juvenile | | MPC-D100/763 | |
|---|---|---|---|---|---|---|---|---|---|---|
| | R | L | R | L | R | L | R | L | R | L |
| Length of skull, from base of occipital condyle to anterior margin of premaxilla in palatial view | 226.00 | | 328.00 | | 340.00 | | 119.11 | | 294.45 | |
| Length of skull, from base of occipital condyle to anterior end of rostroventral process of tha maxilla | 191.04 | | 272.00 | | | | 102.00 | | 251.18 | |
| Width of skull on orbital region | 77.94 | | 160.25 | | 101.30 | | 29.59 | | 82.27 | |
| Width of skull on occipital region | 87.71 | | 103.66 | | 117.58 | | 45.50 | | 114.34 | |
| Height of occiput | 128.43 | | 101.47 | | | | 40.81 | | 101.44 | |
| Transverse width in the postorbital region in dorsal view | 116.30 | | 161.27 | | 155.26 | | 55.51 | | 141.50 | |
| Length of maxillary tooth row | 114.00 | 112.00 | 158.49 | 156.22 | 168.49 | 168.02 | 57.30 | 58.42 | 149.23 | 148.33 |
| Width between two anterior ends of maxilla | 25.39 | | 57.72 | | | | 16.06 | | 38.29 | |
| Width between two posterior ends of maxilla | 78.43 | | 104.16 | | | | 30.33 | | 83.89 | |
| Width between two mandibular condyle of quadrate (from laterial surface) | 160.51 | | 184.07 | | 197.58 | | 68.44 | | 162.86 | |
| Width of skull on anterior margin of orbit at jugal and lacrimal suture | 101.63 | | 111.75 | | 119.59 | | 29.15 | | 88.35 | |
| Greatest length of parietal along midline | 37.63 | 37.80 | 49.87 | | 59.64 | | 28.61 | | 54.96 | |
| Greatest width across anterior end | 48.80 | 60.10 | 75.38 | | 71.89 | | 30.30 | | 70.09 | |
| Greatest width across posterior end | 34.23 | 34.00 | 40.14 | | 45.99 | | 24.47 | | 45.08 | |
| Parietal crest length | 19.40 | | 24.80 | | 31.52 | | 12.45 | | 29.41 | |
| Greatest length of frontal dorsal surface on median line | 46.29 | 46.30 | 53.85 | | 61.56 | | 30.93 | | 51.94 | 49.26 |
| Greatest width of frontals | 90.68 | | 127.79 | | 126.52 | | 40.57 | | 113.13 | |
| Depth of nasal suture on the frontal | 15.75 | 15.90 | 21.61 | 23.20 | 24.26 | 23.11 | | | 20.71 | 22.43 |
| Length of braincase | 74.25 | | 100.52 | | 101.14 | | 35.06 | | | |
| Width of braincase | 41.63 | | 57.97 | | 59.89 | | 24.51 | | | |
| Length of frontal at orbit margin | 20.59 | 19.74 | 19.70 | 20.15 | 13.18 | 15.51 | 12.59 | 11.86 | 15.35 | 16.07 |
| Length of prefrontal | 63.45 | 61.21 | 90.02 | 88.20 | 79.75 | 74.03 | 36.96 | 35.36 | 95.47 | 93.04 |
| Height of maxilla, from joint point of J,L and Mx to margin of alveolar through | 34.75 | 33.37 | 42.30 | 42.70 | 35.91 | 35.93 | 12.04 | 14.39 | 34.78 | 32.84 |
| Length of orbit, from suture of prefrontal and lacrimal to joint point of jugallar process postorbital and postorbitallar process of jugal | 58.05 | 54.56 | 66.23 | 67.21 | | 71.55 | 34.71 | 33.63 | 64.28 | 67.91 |
| Height of orbit, from prefrontal-frontal suture to lowest margin of anterior part of jugal | 58.36 | 57.80 | 66.54 | 63.30 | | 71.63 | 38.77 | 35.28 | 60.64 | 63.51 |
| Length of infratemporal fenestra, from the suture of postorbital-jugalar process to the quadrate-jugalar suture | 31.95 | 30.52 | 43.57 | 49.46 | | 43.74 | 16.80 | 14.70 | | 43.67 |
| Height of infratemporal fenestra, from postorbital-squamosal suture to lowest edge of infratemporal fenestra | 71.58 | 73.60 | 102.64 | 103.23 | | 112.28 | 42.04 | 39.24 | 91.51 | 103.87 |
| Height, from the highest point of Squamosal condyle to the lowest point of Mandiblar condyle | 115.41 | 116.84 | 164.44 | 162.95 | 157.94 | 160.00 | 63.29 | 63.59 | | 154.41 |
| Length of squamosal condyle | 17.06 | 17.42 | 24.50 | 22.63 | 20.25 | 21.35 | 12.12 | 10.77 | | 21.95 |
| Length of mandibular condyle | 17.10 | 17.65 | 24.78 | 24.38 | 23.68 | 27.03 | 12.59 | 10.95 | | 21.41 |
| Width of mandibular condyle | 27.04 | 27.06 | 39.77 | 40.35 | 37.43 | 38.63 | 13.81 | 15.29 | | 32.17 |
| Diameter of paraquadrate opening | 7.73 | 8.78 | 10.95 | 12.84 | | 11.75 | | | | |
| Length of Jugal, from rostrally pointed process to the posterior margin of QJ-r process | 114.80 | 115.28 | 166.20 | 167.70 | 184.39 | | 63.35 | 57.86 | 156.86 | 154.96 |
| Depth of jugal at constriction below intratemporal fenestra | 15.85 | 16.45 | 23.10 | 23.40 | 28.23 | 27.36 | 9.87 | 8.67 | 24.39 | 24.27 |
| Length of free ventral flange on jugal | 21.91 | 21.34 | 28.10 | 25.90 | 37.46 | 37.48 | 14.55 | 12.07 | 28.48 | 29.69 |
| Length, from rostral margin to posterior end of lat.proc. | 35.14 | | 48.46 | | 50.67 | 54.83 | 18.51 | | 57.23 | |
| Width | 61.35 | | 84.49 | | 81.33 | | 29.54 | | 66.79 | |
| Length of ventral process | 10.17 | | 17.70 | | 22.80 | | 9.59 | | 20.89 | |
| Height of denticle | 2.22 | | 5.41 | | 4.03 | | | | 4.99 | |
| Length of dorsal process | 34.81 | | 48.20 | 49.10 | | | 17.33 | 16.66 | 47.55 | |
| Maximium width of premaxilla | 32.50 | 31.93 | 43.60 | 43.51 | 45.16 | | 20.92 | 18.39 | 37.22 | 36.18 |
| Minimium width of premaxilla | 20.17 | 20.10 | 22.71 | 23.00 | 29.48 | | 16.94 | 12.75 | 22.05 | 21.15 |
| Length of external nares | 22.43 | 21.46 | 30.75 | 30.06 | 34.44 | | 12.03 | 10.26 | 38.57 | 36.15 |
| Length of latero-ventral process of premaxilla | 125.98 | 128.36 | 183.00 | 185.20 | 167.61 | | 55.41 | 54.43 | 151.26 | 155.95 |
| Length of oral portion of premaxila, from oral margin to rostral process of maxilla | 42.45 | 40.43 | 51.90 | 58.90 | 58.40 | | 27.79 | 20.40 | 60.96 | 60.48 |

| | | | | | | | | | | |
|---|---|---|---|---|---|---|---|---|---|---|
| Length, from rostral end of predentary process to posterior margin of articular | 219.41 | 221.93 | 294.00 | 295.00 | 314.57 | | 113.02 | 108.29 | 270.65 | 279.60 |
| Height of ramus at center of tooth row | 37.18 | 35.08 | 48.18 | 51.61 | 46.83 | | 15.00 | 14.79 | 38.65 | 41.73 |
| Tooth crown length at center of tooth row | | 27.62 | 25.83 | 30.42 | 30.83 | | | | | |
| Tooth crown width at center of tooth row | 10.06 | 9.75 | 11.90 | 12.27 | 10.23 | | | | | |
| Length of diastema | 14.32 | 15.44 | 15.56 | 15.40 | 18.85 | | | | 19.79 | 19.19 |
| Length of tooth row | 110.27 | 109.19 | 159.25 | 161.89 | 158.33 | | 63.72 | 57.17 | 150.09 | 144.32 |
| Height of coronoid process | 87.28 | 84.69 | 110.82 | 111.17 | 98.25 | | 40.11 | 33.10 | 106.92 | 104.75 |
| Length of coronoid process | 28.12 | 25.50 | 31.29 | 30.59 | | | | | 34.41 | 33.50 |
| Retroarticular length | 32.62 | 32.18 | 49.50 | 53.20 | 41.49 | | 21.22 | 15.75 | 53.85 | 53.56 |
| Symphysis length | 21.32 | 21.26 | 23.90 | 23.60 | 31.22 | | | | | |

| Register No. | Specimen No | Position | Length of Jugal, from rostrally pointed process to the posterior margin of QJ-r process | Depth of jugal at constriction below intratemporal fenestra | Length of free ventral flange on jugal | |
|---|---|---|---|---|---|---|
| | | | 33 | 34 | 35 | |
| MPC-D100/710 | 001-001 | | 129.43 | 17.65 | 22.53 | |
| | | | | | | |
| Register No. | Specimen No | Position | Greatest length dorsal surface on median line | Greatest width of frontals | Depth of nasal suture | Length of frontal at orbit margin |
| MPC-D100/746 | 036-001 | R | 46.29 | 92.43 | 15.75 | 20.59 |
| | 036-002 | L | 46.30 | 91.70 | 15.90 | 19.74 |
| MPC-D100/754 | | R | 53.85 | 127.79 | 21.61 | 19.70 |
| | | | | | | |
| MPC-D100/752 | 042-001 | R | 61.56 | 126.52 | 24.26 | 13.18 |
| | 042-002 | L | | | 23.11 | 15.51 |
| MPC-D100/715 | 006-001 | R | 52.40 | 108.72 | 18.95 | 17.98 |
| | 006-002 | L | 48.30 | | 17.32 | 17.82 |
| MPC-D100/716 | 007-001 | R | 40.22 | 52.75 | 17.01 | 14.42 |

| Cervical vertebra | | | | | | | | | | | | | | | | |
|---|---|---|---|---|---|---|---|---|---|---|---|---|---|---|---|---|
| | | | | | | | | | | | | | | | | |
| Register No. | Specimen No. | Position | Length of the centrum, the longest axis of centrum | Height of the centrum anteriorly, from the bottom of the neural canal to the ventral rim of the centrum | Width of the centrum anteriorly, between lateral rims of the centrum in center | Height of the neural canal anteriorly, from the roof of the neural canal to the bottom of the neural canal | Length of transverse processes, between right and left lateral rims of transverse processes | Width of postzygapophysis, between the lateral rims of right and left postzygapophysis | Height of the neural spine | Greatest width of centrum posteriorly | Length of centrum ventrally | Length of the centrum on dorsal margin, excluded anterior cotyle | Height of the centrum posteriorly | Length of the centrum in ventral margin, excluded anterior cotyle | Height of the centrum anteriorly | Height of the neural spine prependicularly along the anteroposterior axis of the vertebra, from the roof of the neural canal to the tip of the neural spine |
| | | | 1 | 2 | 3 | 4 | 5 | 6 | 7 | 8 | 9 | AB | BC | DC | AD | 13 |
| MPC-D100/746 | 036-055 | C1At | 12.01 | 9.62 | 33.74 | 25.25 | | | | | | | | | | |
| | 036-056 | C3 | | | | | | | | | | | | | | |
| | 036-057 | C4 | | | | 14.16 | | 36.56 | 19.56 | 31.15 | | 17.53 | 26.34 | | | 6.70 |
| | 036-058 | C5 | 40.90 | 23.52 | 24.23 | 14.94 | 53.06 | 40.23 | 20.28 | 32.12 | 39.43 | 28.82 | 23.47 | 28.27 | 26.60 | 7.72 |
| | 036-059 | C6 | 38.80 | 24.50 | 25.07 | 16.74 | 65.56 | 44.99 | 18.64 | 33.25 | 39.50 | 28.30 | 26.41 | 27.48 | 26.43 | 9.61 |
| | 036-060 | C7 | 40.85 | 21.90 | 25.48 | 16.65 | 69.09 | 47.82 | 17.23 | 35.11 | 39.64 | 28.40 | 22.63 | 30.55 | 24.28 | 8.22 |
| | 036-061 | C8 | 40.06 | 23.95 | 25.70 | 16.68 | 74.35 | 52.29 | 19.76 | 35.83 | 39.25 | 23.99 | 24.62 | 28.97 | 25.54 | 8.23 |
| | 036-062 | C9 | 40.01 | 24.23 | 26.96 | 14.03 | 86.52 | 52.34 | 21.99 | 36.99 | 38.29 | 27.10 | 22.66 | 27.98 | 24.62 | 9.97 |
| | 036-063 | C10 | 40.31 | 23.41 | 28.35 | 16.43 | 98.85 | 56.87 | 17.83 | 34.07 | 39.40 | 28.53 | 24.35 | 28.90 | 24.75 | 10.42 |
| | 036-064 | C11 | 39.62 | 23.47 | 24.04 | 14.13 | 107.94 | 55.92 | 18.50 | 33.60 | 38.86 | 26.71 | 26.15 | 27.01 | | 11.72 |
| | | | | | | | | | | | | | | | | |
| MPC-D100/752 | | C1 | | | | | | | | | | | | | | |
| | | C2 | | | | | | | | | | | | | | |
| | 042-054 | C3 | | | | | 75.31 | 66.08 | 29.06 | | | | | | | 6.46 |
| | 042-055 | C4 | | | | 22.19 | 84.25 | | 23.62 | | | | | | | 7.5 |
| | 042-056 | C5 | 42.59 | 32.59 | 36.00 | 19.27 | 104.26 | 82.37 | 24.89 | 44.77 | 39.41 | 37.43 | | | | 8.22 |

| | | | | | | | | | | | | | | | | |
|---|---|---|---|---|---|---|---|---|---|---|---|---|---|---|---|---|
| | 042-057 | C6 | | | | 24.17 | | 85.35 | | 46.64 | 40.87 | 33.66 | 32.09 | 33.13 | 31.17 | 8.31 |
| | 042-058 | C7 | | 33.44 | 40.54 | 22.33 | 106.81 | 86.33 | 24.05 | 48.61 | 41.37 | 39.79 | | | | 9.93 |
| | 042-059 | C8 | 45.87 | 35.18 | 39.33 | | 125.34 | | | 49.44 | 46.91 | 39.81 | 39.94 | 35.04 | 35.30 | |
| | 042-060 | C9 | | 37.52 | 43.62 | 26.85 | | | | | | | | | | |
| | 042-061 | C10 | 48.45 | 31.80 | 39.26 | 24.33 | 168.15 | 90.95 | 26.28 | 49.53 | 43.62 | | | | | 16.5 |
| | 042-062 | C11 | | | | | | | | | | | | | | |

| Dorsal vertebra | | | | | | | | | | | | | | |
|---|---|---|---|---|---|---|---|---|---|---|---|---|---|---|
| | | | | | | | | | | | | | | |
| Register No. | Specimen No. | Position | Length of centrum, the longest axis of centrum | Height of centrum anteriorly, from the bottom of the neural canal to ventral rim of centrum | Width of centrum anteriorly, between lateral rims of centrum in center | Height of neural spine anteriorly, from the roof of the neural canal to the dorsal rim of the spine | Height of neural canal anteriorly, from the roof of the neural canal to the bottom of the neural canal | Width of the neural spine in dorsal end | Length of the lateral wall of the neural canal | Length of the neural spine in dorsal end | Length of the right transverse process, from the base of prespinal lamina to the center of lateral rim of right transverse process | Length of left transverse process, from the base of prespinal lamina to the center of lateral rim of left transverse process | Height of neural spine prependicularly along the anteroposterior axis of the vertebra, from the roof of the neural canal to | Angle between the along of the neural canal roof and inclination of the neural spine |
| | | | 1 | 2 | 3 | 4 | 5 | 6 | 7 | 8 | 9 | 10 | 13 | 14 |
| MPC-D100/746 | 036-080 | D1 | 49.58 | 60.30 | 80.63 | | | | | | | | | |
| | 036-081 | D2 | 40.51 | 22.53 | 27.23 | 26.12 | 14.45 | 5.82 | 17.36 | 8.14 | 48.10 | 57.98 | 20.75 | 48.00 |
| | 036-082 | D3 | 40.70 | 22.48 | 27.39 | | 15.66 | | 19.85 | | 51.47 | 54.28 | | |
| | 036-083 | D4 | 43.82 | 25.00 | 29.92 | 67.62 | 15.96 | 4.48 | 23.39 | 11.77 | 42.22 | 61.57 | 32.06 | 21.50 |
| | 036-084 | D5 | 45.42 | 28.18 | 31.74 | 90.08 | 15.94 | 5.13 | 27.58 | 14.98 | 39.90 | 61.52 | 40.79 | 17.70 |
| | 036-085 | D6 | 46.19 | 29.58 | 30.95 | 95.74 | 16.72 | 6.66 | 29.41 | 21.17 | 53.55 | | 64.78 | 53.50 |
| | 036-086 | D7 | 46.90 | 28.02 | 32.10 | 112.61 | 16.16 | 5.93 | 30.62 | 20.87 | 46.83 | 48.27 | 65.86 | 30.50 |
| | 036-087 | D8 | 46.97 | 32.32 | 31.27 | 94.47 | 14.52 | 6.74 | 30.48 | 28.74 | 52.98 | 55.81 | 53.13 | 40.00 |
| | 036-088 | D9 | 47.96 | 30.31 | 33.33 | 115.03 | 12.78 | 8.12 | 30.33 | 27.94 | 56.92 | 48.74 | 73.15 | 34.00 |
| | 036-089 | D10 | 48.05 | 31.84 | 30.70 | 102.40 | 14.28 | 8.87 | 29.85 | 29.07 | 49.24 | 52.62 | 93.83 | 50.00 |
| | 036-090 | D11 | 48.35 | 36.80 | 30.94 | 109.31 | 12.95 | 10.12 | 31.98 | 33.78 | 45.68 | 55.64 | 94.29 | 72.00 |
| | 036-091 | D12 | 43.68 | 35.94 | 35.44 | 111.19 | 13.48 | 10.14 | 30.64 | 34.42 | 48.82 | 53.02 | 96.08 | 61.50 |
| | 036-092 | D13 | 42.25 | 36.52 | 35.91 | 98.91 | 13.14 | 9.82 | 29.51 | 40.87 | 49.74 | 54.60 | 90.74 | 75.00 |
| | 036-093 | D14 | 41.17 | 38.26 | 38.07 | 97.80 | 9.92 | 13.19 | 28.90 | 36.16 | 45.95 | 52.25 | 91.31 | 80.00 |
| | 036-094 | D15 | 39.24 | 40.44 | 41.71 | 99.44 | 11.37 | 10.04 | 27.28 | 32.05 | | 53.16 | 103.68 | 83.50 |
| | 036-095 | D16 | 37.54 | 39.30 | 43.81 | 101.40 | 15.93 | 6.82 | 25.83 | 34.32 | 42.04 | 47.02 | 103.50 | 86.00 |
| | | | 38.57 | 39.09 | 45.16 | 108.67 | 13.71 | 6.48 | 27.04 | 34.84 | 44.25 | 50.08 | 107.22 | 90.00 |
| | | | | | | | | | | | | | | |
| MPC 100/752 | 042-063 | D1 | | 34.77 | 41.17 | | 19.93 | | | | 70.78 | 88.08 | | |
| | 042-064 | D2 | 60.91 | 33.93 | 43.46 | | 17.82 | | 37.09 | | 79.25 | | | 40.5 |
| | 042-065 | D3 | | | | 169.83 | 22.54 | | 40.54 | 22.60 | 70.92 | | 89.01 | 32.0 |
| | 042-066 | D4 | 53.36 | 62.58 | 47.55 | 168.8 | 22.76 | 14.45 | 37.95 | 28.72 | 88.72 | | 101.64 | 40.0 |
| | 042-067 | D6 | 53.85 | 58.48 | 51.12 | 155.48 | | 15.84 | 39.52 | 31.87 | 80.46 | | 109.6 | 57.6 |
| | 042-068 | D7 | | | 54.00 | | | | | | 78.91 | | | |
| | 042-069 | D9 | 52.96 | 42.57 | 55.19 | | 16.62 | | 39.57 | | 86.89 | 82.50 | | 70.5 |
| | 042-070 | D10 | 52.4 | 41.10 | 49.04 | 162.88 | 22.78 | 10.70 | 35.32 | 39.72 | 74.95 | | 150.84 | 84.5 |
| | 042-071 | D13 | 47.50 | 62.06 | 59.40 | 191.79 | 20.05 | 9.39 | 33.28 | 45.26 | | 109.64 | 180.99 | 87.5 |
| | 042-072 | D15 | | 67.24 | 80.42 | 201.38 | | 28.49 | | 52.20 | 102.45 | | 194.99 | 90.0 |
| | 042-073 | D16 | 58.55 | 69.55 | 61.19 | | | | | | | | | |

| Caudal vertebra | | | | | | | | | | | | | | | |
|---|---|---|---|---|---|---|---|---|---|---|---|---|---|---|---|
| | | | | | | | | | | | | | | | |
| Register No. | Specimen No. | Position | Greatest length of centrum, the longest axis of the centrum | Height of centrum in cranial articular surface, from the bottom of the neural canal to the center of articular facet of chevron | Width of centrum in dorsal aspect anteriorly, between dorsolateral margins of the centrum | Width of centrum in ventral aspect anteriorly, between lateroventral margins of the centrum | Height of the neural spine anteriorly, from the roof of the neural canal to the tip of the neural spine | Height of the neural canal in anterior aspect | Length of base of the neural spine | Width of dorsal rim of the neural spine | Length of left caudal rib, from the center of the neural canal roof to the center of the lateral rim of left caudal rib | Length of right caudal rib, from the center of the neural canal roof to the center of the lateral rim of right caudal rib | Greatest length of the dorsal end of neural spine | Height of the neural spine prependicularly along the anteroposterior axis of the vertebra , from the roof of the neural canal to the tip of the neural spine | Angle between the longest axis of the centrum and inclination of the neural spine |
| | | | 1 | 2 | 3 | 4 | 5 | 7 | 8 | 9 | 10 | 11 | 12 | 13 | 14 |
| MPC-D100/714 | 005-001 | | 42.58 | 40.14 | 42.66 | 27.63 | 137.78 | 9.2 | 25.4 | 12.61 | | | 21.35 | 70.31 | 32.50 |
| | 005-002 | | 42.05 | 27.68 | 39.53 | 27.7 | | 6.25 | 21.38 | | | | | | |
| | | | | | | | | | | | | | | | |
| MPC-D100/728 | 019-001 | Cd12 | 42.83 | 42.44 | 43.47 | 28.60 | 148.99 | 9.90 | 25.28 | 19.07 | | | 24.10 | 58.87 | 27.00 |
| | 019-002 | Cd13 | 42.35 | 40.11 | 42.16 | 27.98 | 131.44 | 9.57 | 25.05 | 12.51 | | | 19.80 | 55.55 | 26.50 |
| | 019-003 | Cd14 | 41.73 | 39.01 | 41.42 | 26.89 | 123.07 | 8.83 | 23.49 | 12.17 | | | 19.08 | 52.42 | 26.00 |
| | 019-004 | Cd15 | 42.08 | 38.20 | 40.82 | 25.06 | 116.79 | 8.37 | 23.19 | 12.15 | | | 19.82 | 51.68 | 26.50 |
| | 019-005 | Cd16 | 41.58 | 37.49 | 39.94 | 23.31 | 110.38 | 7.74 | 22.71 | 11.38 | | | 18.74 | 51.50 | 27.00 |
| | 019-006 | Cd17 | 39.99 | 35.70 | 40.29 | 22.89 | 102.74 | 7.45 | 21.48 | 12.72 | | | 15.32 | 43.73 | 28.00 |
| | 019-007 | Cd18 | 40.12 | 34.01 | 38.86 | 23.11 | 95.21 | 7.70 | 21.67 | 12.66 | | | 15.52 | 36.84 | 28.00 |
| | 019-008 | Cd19 | 39.10 | 33.08 | 38.44 | 22.50 | 88.72 | 7.21 | 21.14 | 9.75 | | | 12.90 | 34.73 | 29.00 |
| | 019-009 | Cd20 | 37.28 | 31.98 | 38.11 | 21.69 | | 7.01 | 21.99 | | | | | | 30.00 |
| | 019-010 | Cd21 | 38.03 | 31.00 | 37.23 | 21.48 | | 6.82 | 21.55 | | | | | 30.34 | 30.50 |
| | 019-011 | Cd22 | 36.72 | 28.51 | 32.68 | 22.96 | | 6.44 | 20.10 | | | | | 22.84 | |
| | 019-012 | Cd23 | 37.03 | 27.33 | 33.49 | 22.68 | 57.20 | 6.37 | 20.12 | | | | | | 31.00 |
| | 019-013 | Cd24 | 35.92 | 27.8 | 32.59 | 19.80 | | 5.95 | 17.41 | | | | | | 32.00 |
| | 019-014 | Cd25 | 35.49 | 26.54 | 30.99 | 19.24 | | 5.87 | 16.99 | | | | | | 33.00 |
| | 019-015 | Cd26 | | | | | | | | | | | | | |

| | | | | | | | | | | | | | | | |
|---|---|---|---|---|---|---|---|---|---|---|---|---|---|---|---|
| | | | | | | | | | | | | | | | |
| MPC-D100/729 | 020-001 | Cd | 43.67 | 47.01 | 49.43 | 30.39 | 172.38 | 10.75 | 25.81 | 13.90 | | | 29.23 | 93.65 | |
| | | | | | | | | | | | | | | | |
| MPC-D100/738 | 028-001 | Cd-1 | 45.83 | 75.55 | 84.45 | | 239.92 | 14.54 | 30.79 | 17.58 | 108.73 | | 39.58 | 214.56 | 65.00 |
| | | | | | | | | | | | | | | | |
| MPC-D100/746 | 036-131 | Cd1 | 31.93 | 42.32 | 52.23 | | 140.92 | 10.56 | 22.36 | 6.17 | 76.15 | 75.10 | 25.86 | 119.07 | 63.50 |
| | 036-132 | Cd2 | 30.56 | 44.72 | 50.77 | | 143.77 | 10.96 | 17.20 | 7.09 | 78.75 | 78.32 | 22.16 | 121.24 | 57.00 |
| | 036-133 | Cd3 | 31.85 | 45.67 | 49.84 | | 138.41 | 10.83 | 17.27 | 6.29 | 70.24 | 70.68 | 22.05 | 111.96 | 57.50 |
| | 036-134 | Cd4 | 30.93 | 45.40 | 47.76 | 25.50 | 133.87 | 10.98 | 18.01 | 7.20 | 67.53 | 67.13 | 22.25 | 97.71 | 52.00 |
| | 036-135 | Cd5 | 31.59 | 42.01 | 46.37 | 29.52 | 135.37 | 11.78 | 17.33 | 7.50 | 60.88 | 60.02 | 21.76 | 97.61 | 56.50 |
| | 036-136 | Cd6 | 30.34 | 42.02 | 43.10 | 28.66 | 134.46 | 11.36 | 17.31 | 6.96 | 56.15 | 48.26 | 23.49 | 92.21+ | 53.00 |
| | 036-137 | Cd7 | 31.44 | 40.80 | 42.39 | 27.13 | 135.66 | 10.51 | 17.27 | 8.95 | 51.60 | 51.85 | 23.80 | 80.52 | 43.50 |
| | 036-138 | Cd8 | 31.88 | 38.92 | 40.82 | 27.30 | 136.56 | 10.98 | 19.11 | 8.80 | 45.32 | 48.29 | 23.42 | 79.06 | 38.50 |
| | 036-139 | Cd9 | 31.51 | 38.39 | 39.40 | 26.67 | 140.31 | 10.50 | 18.80 | 8.49 | 38.28 | 43.59 | 20.45 | 76.58 | 35.50 |
| | 036-140 | Cd10 | 30.97 | 35.85 | 37.65 | 25.18 | 135.05 | 10.18 | 19.09 | 8.41 | 32.80 | 30.85 | 21.51 | 75.04 | 36.00 |
| | 036-141 | Cd11 | 32.31 | 36.22 | 37.65 | 24.65 | 136.66 | 9.79 | 18.74 | 8.94 | 32.20 | | 18.50 | 63.28 | 30.00 |
| | 036-142 | Cd12 | 32.30 | 32.57 | 33.71 | 23.16 | 137.09 | 9.81 | 18.68 | 7.01 | 26.26 | 25.68 | 18.62 | 64.16 | 28.00 |
| | 036-143 | Cd13 | 33.74 | 33.03 | 33.97 | 20.13 | 124.61 | 9.27 | 20.22 | 8.18 | | | 18.13 | 58.37 | 28.20 |
| | 036-144 | Cd14 | 33.61 | 33.06 | 35.98 | 20.07 | 124.64 | 8.87 | 20.34 | 8.13 | | | 15.49 | 63.17 | 25.50 |
| | 036-145 | Cd15 | 33.75 | 31.47 | 33.69 | 22.24 | 129.20 | 8.46 | 19.96 | 7.29 | | | 14.85 | 60.39 | 26.00 |
| | 036-146 | Cd16 | 33.32 | 32.22 | 34.17 | 21.37 | 128.30 | 8.39 | 20.38 | 8.80 | | | 15.34 | 62.42 | 27.50 |
| | 036-147 | Cd17 | 34.26 | 31.97 | 33.37 | 21.27 | 124.96 | 7.27 | 20.68 | 8.28 | | | 13.69 | 55.90 | 26.00 |
| | 036-148 | Cd18 | 33.73 | 30.44 | 32.54 | 22.55 | 119.73 | 8.05 | 20.86 | 7.45 | | | 12.62 | 48.10 | 18.00 |
| | 036-149 | Cd19 | 33.40 | 29.80 | 32.66 | 21.52 | 113.39 | 7.90 | 20.65 | 7.09 | | | 12.79 | 43.41 | 21.50 |
| | 036-150 | Cd20 | 33.90 | 29.89 | 32.39 | 21.71 | 106.59 | 8.33 | 19.91 | 7.04 | | | 11.68 | 43.21 | 25.00 |
| | 036-151 | Cd21 | 32.80 | 29.46 | 31.27 | 21.32 | 100.23 | 7.26 | 19.83 | 7.14 | | | 11.99 | 33.95 | 27.00 |
| | 036-152 | Cd22 | 32.12 | 28.45 | 30.50 | 21.15 | 98.06 | 7.19 | 19.26 | 8.26 | | | 11.11 | 35.41 | 24.50 |
| | 036-153 | Cd23 | 33.24 | 28.05 | 29.73 | 20.40 | 94.52 | 7.38 | 17.48 | 6.24 | | | 11.36 | 37.72 | 25.00 |
| | 036-154 | Cd24 | 32.56 | 27.39 | 29.84 | 20.36 | 90.64 | 6.76 | 16.93 | 6.87 | | | 10.93 | 36.83 | 21.00 |
| | 036-155 | Cd25 | 31.68 | 26.00 | 29.20 | 19.91 | 86.71 | 6.66 | 16.17 | 7.36 | | | 11.17 | 31.23 | 17.00 |
| | 036-156 | Cd26 | 31.55 | 24.74 | 28.96 | 19.71 | 81.80 | 6.51 | 17.85 | 5.24 | | | 10.67 | 29.78 | 20.80 |
| | 036-157 | Cd27 | 31.81 | 23.31 | 28.90 | 19.53 | 77.30 | 6.49 | 15.16 | 4.92 | | | 8.69 | 27.55 | 17.00 |
| | 036-158 | Cd28 | 30.70 | 23.15 | 27.67 | 18.73 | 70.15 | 4.27 | 16.84 | 4.23 | | | 9.10 | 26.93 | 16.50 |
| | 036-159 | Cd29 | 30.16 | 22.43 | 26.88 | 18.34 | 66.07 | 5.94 | 14.94 | 4.45 | | | 8.21 | 24.21 | 17.50 |
| | 036-160 | Cd30 | 29.83 | 21.99 | 26.16 | 17.87 | 62.82 | 5.46 | 15.86 | 3.55 | | | 7.31 | 24.20 | 18.00 |
| | 036-161 | Cd31 | 30.03 | 22.37 | 25.98 | 17.13 | 57.18 | 5.53 | 15.58 | 2.99 | | | 6.49 | 20.42 | 19.00 |
| | 036-162 | Cd32 | 28.87 | 21.76 | 24.75 | 16.11 | 53.67 | 5.35 | 12.48 | 2.88 | | | 5.16 | 20.23 | 20.00 |
| | 036-163 | Cd33 | 28.32 | 21.41 | 24.20 | 15.47 | 48.72 | 5.05 | 13.27 | 2.87 | | | 5.13 | 19.2 | 21.00 |
| | 036-164 | Cd34 | 28.23 | 20.07 | 23.87 | 15.08 | 46.37 | 4.64 | 13.35 | 3.10 | | | 5.09 | 17.15 | 22.00 |
| | 036-165 | Cd35 | 27.86 | 19.51 | 23.52 | 13.81 | 45.08 | 4.89 | 13.91 | 3.02 | | | 4.61 | 13.93 | 22.50 |
| | 036-166 | Cd36 | 26.92 | 18.84 | 22.68 | 13.06 | 34.15 | 4.49 | 10.98 | 3.91 | | | 4.10 | 12.38 | 18.00 |

| | | | | | | | | | | | | | | | |
|---|---|---|---|---|---|---|---|---|---|---|---|---|---|---|---|
| | 036-167 | Cd37 | 25.93 | 17.78 | 21.80 | 13.74 | 32.50 | 4.48 | 10.94 | 3.76 | | | 4.00 | 10.84 | 20.00 |
| | 036-168 | Cd38 | 25.90 | 17.25 | 21.02 | 13.04 | 31.60 | 4.50 | 10.70 | 4.34 | | | 4.87 | 10.18 | 22.50 |
| | 036-169 | Cd39 | 25.01 | 17.06 | 20.15 | 12.66 | 21.28 | 4.25 | 10.69 | 2.98 | | | 4.03 | 9.84 | 19.00 |
| | 036-170 | Cd40 | 24.73 | 16.83 | 20.23 | 12.60 | 26.35 | 4.32 | 10.22 | 3.94 | | | 4.04 | 9.34 | 18.00 |
| | 036-171 | Cd41 | 23.45 | 15.98 | 19.89 | 12.46 | 24.30 | 4.09 | 9.11 | 4.26 | | | 3.44 | 7.57 | 20.00 |
| | 036-172 | Cd42 | 23.29 | 15.95 | 19.54 | 12.76 | 21.33+ | 3.97 | 8.90 | - | | | - | 5.55 | 10.00 |
| | 036-173 | Cd43 | 22.57 | 15.29 | 18.74 | 12.39 | 21.19 | 3.93 | 8.10 | 4.09 | | | 3.60 | 5.33 | 11.00 |
| | 036-174 | Cd44 | 22.33 | 15.05 | 17.84 | 12.26 | 19.24 | 4.06 | 8.09 | 4.19 | | | 3.49 | 5.57 | 14.00 |
| | 036-175 | Cd45 | 21.85 | 14.16 | 17.19 | 10.99 | 18.66 | 3,22 | 7.45 | 3.73 | | | 3.56 | 4.93 | 15.00 |
| | 036-176 | Cd46 | 20.49 | 13.36 | 16.22 | 11.22 | 20.63 | 3.16 | 6.58 | 4.43 | | | 3.36 | 4.87 | 10.00 |
| | 036-177 | Cd47 | 20.03 | 13.33 | 15.43 | 10.05 | 18.05 | 2.95 | 6.46 | 4.16 | | | 3.02 | 4.19 | 10.00 |
| | 036-178 | Cd48 | 19.93 | 12.69 | 14.47 | 10.72 | 17.06 | 2.93 | 5.65 | 3.76 | | | 3.03 | 3.94 | 8.00 |
| | 036-179 | Cd49 | 18.89 | 12.57 | 14.22 | 8.93 | 12.25 | 2.94 | 4.95 | 2.95 | | | 2.39 | 3.68 | 10.00 |
| | 036-180 | Cd50 | 18.17 | 12.12 | 13.83 | 8.56 | 9.89 | 2.84 | 5.04 | 3.24 | | | 2.19 | 3.54 | 7.00 |
| | 036-181 | Cd51 | 17.91 | 11.34 | 13.25 | 7.33 | 8.33 | 3.34 | 4.65 | 2.73 | | | 2.32 | 2.90 | 5.00 |
| | 036-182 | Cd52 | 17.14 | 11.27 | 13.20 | 7.91 | 10.23 | 2.86 | 4.48 | 2.06 | | | 1.64 | 2.11 | 5.00 |
| | 036-183 | Cd53 | 16.17 | 10.78 | 11.91 | 6.75 | 8.60 | 2.40 | 4.25 | 2.32 | | | 2.14 | 2.35 | |
| | 036-184 | Cd54 | 15.84 | 10.49 | 11.22 | 7.96 | 8.68 | 3.37 | 4.64 | 2.47 | | | 2.53 | 2.17 | |
| | 036-185 | Cd55 | 16.49 | 10.23 | 10.70 | 7.39 | 8.66 | 3.03 | 4.14 | 2.32 | | | 1.84 | 3.09 | |
| | 036-186 | Cd56 | 16.66 | 9.61 | 10.34 | 5.98 | 8.79 | 3.90 | 3.47 | 1.88 | | | 1.99 | 4.60 | |
| | 036-187 | Cd57 | 16.34 | 9.81 | 11.22 | | 10.54 | 3.36 | 3.43 | 2.33 | | | 2.14 | 5.37 | |
| | 036-188 | Cd58 | 15.58 | 9.20 | 10.48 | | | | | | | | | | |
| | | | | | | | | | | | | | | | |
| Specimens from BS bonebed | | | | | | remarks | | | | | | | | | |
| MPC-D100/750 | 040-073 | Cd.ant. | 19.81 | 27.17 | 32.14 | ve59 | | | | | | | | | |
| | 040-074 | Cd.ant. | 19.3 | 27.20 | 29.06 | ve62 | | | | | | | | | |
| | 040-075 | Cd.ant. | 15.18 | 26.99 | 26.99 | ve61 | | | | | | | | | |
| | 040-076 | Cd.ant. | 16.03 | 25.44 | 28.26 | ve63 | | | | | | | | | |
| | 040-077 | Cd.ant. | 19.06 | 25.07 | 25.50 | ve74 | | | | | | | | | |
| | 040-078 | Cd.ant. | 18.67 | 21.08 | 26.28 | ve75 | | | | | | | | | |
| | 040-079 | Cd.mid. | 14.32 | 21.38 | 24.17 | ve69 | | | | | | | | | |
| | 040-080 | Cd.mid. | 13.72 | 22.13 | 24.19 | ve70 | | | | | | | | | |
| | 040-081 | Cd.mid. | 12.93 | 20.29 | 24.18 | ve71 | | | | | | | | | |
| | 040-082 | Cd.mid. | 11.73 | 18.11 | 23.02 | ve78 | | | | | | | | | |
| | 040-083 | Cd.mid. | 9.74 | 10.98 | 13.96 | Ve82 | | | | | | | | | |
| | 040-084 | Cd.mid. | 9.61 | 12.21 | 13.98 | Ve83 | | | | | | | | | |
| | 040-085 | Cd.mid. | 9.56 | 12.28 | 13.75 | ve72 | | | | | | | | | |
| | 040-086 | Cd.mid. | 9.63 | 9.74 | 12.05 | ve81 | | | | | | | | | |
| | 040-087 | Cd.mid. | 8.66 | 10.73 | 13.67 | Ve84 | | | | | | | | | |
| | | | | | | | | | | | | | | | |

| | | | | | | | | | | | | | | | |
|---|---|---|---|---|---|---|---|---|---|---|---|---|---|---|---|
| MPC-D100/752 | 042-074 | Cd1 | 42.63 | 66.27 | 77.95 | 43.32 | | 22.97 | 25.14 | | 107.64 | | | | 68.50 |
| | 042-075 | Cd2 | 42.03 | 63.91 | 64.79 | 31.27 | 233.50 | 18.06 | 26.62 | 13.08 | | | 34.12 | 170.00 | 50.00 |
| | 042-076 | Cd3 | 45.93 | 59.76 | 52.55 | | | 11.92 | 31.73 | | 52.63 | | | | |
| | 042-077 | Cd4 | 46.97 | 54.87 | 54.8 | 25.29 | 220.04 | 15.63 | 31.20 | 12.19 | | | 25.78 | 99.25 | 32.00 |
| | 042-078 | Cd5 | 42.19 | 56.5 | 54.04 | 27.90 | 219.97 | 15.35 | 30.97 | 11.98 | | | 23.58 | 97.49 | 25.00 |
| | 042-079 | Cd6 | 47.55 | 48.18 | 52.72 | | | | | | | | | | |
| | 042-080 | Cd7 | 48.01 | | 51.49 | 33.11 | | | 34.43 | | | | | | 18.00 |
| | 042-081 | Cd8 | 46.04 | 45.32 | 49.52 | 24.97 | | 15.55 | 25.85 | | | | | | 20.00 |
| | 042-082 | Cd9 | 46.99 | 45.38 | 46.05 | 25.72 | 193.88 | 13.01 | 30.49 | 18.01 | | | 25.34 | 97.43 | 21.00 |
| | 042-083 | Cd10 | 46.30 | 39.37 | 48.98 | 25.32 | 167.68 | 10.63 | 30.23 | 18.17 | | | 21.98 | 74.37 | 22.00 |
| | 042-084 | Cd11 | 46.27 | 38.00 | 49.36 | 24.94 | 159.17 | 9.15 | 29.54 | 13.89 | | | 23.70 | 68.71 | 20.00 |
| | 042-085 | Cd12 | 46.53 | 37.40 | 46.63 | 24.53 | 150.92 | 10.28 | 30.82 | 12.44 | | | 24.12 | 67.01 | 20.50 |
| | 042-086 | Cd13 | 46.93 | 38.50 | | 22.65 | | | | | | | | | |
| | 042-087 | Cd14 | 45.09 | 38.41 | 46.41 | 24.56 | 134.86 | 10.02 | 27.08 | 9.96 | | | 16.92 | 51.76 | 15.00 |
| | 042-088 | Cd15 | 44.87 | 36.81 | 45.22 | 22.79 | 130.17 | 10.32 | 26.1 | 10.21 | | | 20.36 | 54.44 | 16.00 |
| | 042-089 | Cd16 | | 36.70 | 48.49 | 28.93 | | | | | | | | | |
| | 042-090 | Cd17 | 45.26 | 36.73 | 44.20 | 23.19 | 122.97 | 9.97 | 23.72 | 10.82 | | | 18.69 | 40.79 | 15.50 |
| | 042-091 | Cd18 | 44.19 | 35.99 | 40.61 | 21.59 | 113.73 | 11.95 | 22.40 | 7.58 | | | 13.36 | | |
| | 042-092 | Cd19 | 42.66 | 33.97 | 41.86 | 21.13 | | 8.72 | 23.72 | | | | | | |
| | 042-093 | Cd20 | 42.29 | 31.80 | 40.92 | 20.94 | 62.11 | 7.81 | 23.51 | 4.09 | | | 9.71 | | 18.00 |
| | 042-094 | Cd21 | 42.43 | 30.62 | 39.81 | 17.33 | | 6.96 | 20.56 | | | | | | |
| | 042-095 | Cd22 | 42.12 | 29.41 | 39.75 | 14.03 | | | | | | | | | |
| | 042-096 | Cd23 | 28.82 | 24.65 | 27.58 | 10.08 | | 6.60 | 11.87 | | | | | | |
| | 042-097 | Cd24 | | | | | | | | | | | | | |
| | | | | | | | | | | | | | | | |
| MPC-D100/753 | 043-001 | cd33 | 29.40 | 22.70 | 25.56 | 16.13 | | | | | | | | | |
| | 043-002 | cd34 | 29.48 | 24.43 | 24.64 | 16.44 | | | 14.64 | | | | | | |
| | 043-003 | cd35 | 28.12 | 22.41 | 25.55 | 15.87 | 33.90 | 5.30 | 17.73 | 3.83 | | | 6.39 | 16.71 | 33.00 |
| | 043-004 | cd36 | 27.01 | 21.73 | 24.23 | 14.94 | 36.20 | 4.91 | 14.53 | 3.45 | | | 6.09 | 16.92 | 35.00 |
| | 043-005 | cd37 | 27.10 | 21.98 | 23.66 | 13.90 | 28.97 | 4.28 | 13.18 | 3.63 | | | 6.24 | 11.99 | 31.00 |
| | 043-006 | cd38 | 26.29 | 21.66 | 23.33 | 13.91 | 26.90 | 4.88 | 13.09 | 3.30 | | | 6.42 | 15.82 | 40.00 |
| | 043-007 | cd39 | 25.99 | 20.30 | 23.28 | 13.25 | 25.27 | 3.55 | 13.80 | 5.90 | | | 5.85 | 11.54 | 42.00 |
| | 043-008 | cd40 | 24.94 | 18.99 | 23.25 | 13.11 | 20.48 | 4.30 | 10.80 | 3.85 | | | 5.37 | 9.71 | 30.00 |
| | 043-009 | cd41 | 24.63 | 18.10 | 22.51 | 13.44 | 22.94 | 4.85 | 10.33 | 3.82 | | | 5.33 | 7.78 | 15.00 |
| | 043-010 | cd42 | 24.23 | 17.55 | 21.19 | 13.23 | 18.63 | 4.30 | 10.95 | 3.54 | | | 5.05 | 7.47 | 10.00 |
| | 043-011 | cd43 | 22.54 | 17.35 | 19.56 | 13.20 | 20.49 | 4.51 | 9.90 | 3.47 | | | 4.29 | 6.48 | 10.00 |
| | 043-012 | cd44 | 22.58 | 17.59 | 20.60 | 13.11 | 19.24 | 5.05 | 9.60 | 3.20 | | | 4.34 | 5.74 | 9.50 |
| | 043-013 | cd45 | 21.26 | 16.51 | 18.40 | 11.01 | 21.41 | 4.54 | 9.14 | 3.44 | | | 3.89 | 4.41 | 5.00 |
| | 043-014 | cd46 | 20.64 | 15.42 | 17.74 | 11.57 | 16.95 | 4.28 | 7.88 | 2.83 | | | 4.27 | | |
| | 043-015 | cd47 | 19.59 | 15.23 | 17.56 | 11.37 | | 3.60 | 7.43 | | | | | | |

| | | | | | | | | | | | | | | | |
|---|---|---|---|---|---|---|---|---|---|---|---|---|---|---|---|
| | 043-016 | cd48 | 17.76 | 15.10 | 16.80 | 10.78 | 16.94 | 3.24 | 7.39 | 2.92 | | | 3.28 | | |
| | 043-017 | cd49 | 17.42 | 13.51 | 16.08 | 11.06 | | | 7.45 | | | | | | |
| | 043-018 | cd50 | 16.83 | 13.03 | 15.06 | | | | 7.31 | | | | | | |
| | 043-019 | cd51 | 16.08 | 12.21 | 14.10 | | | | | | | | | | |
| | 043-020 | cd52 | 15.98 | 12.03 | 13.26 | | | | 7.24 | | | | | | |
| | 043-021 | cd53 | 14.30 | 10.69 | 12.55 | | | | 6.71 | | | | | | |
| | 043-022 | cd54 | 13.99 | 12.17 | 13.03 | | | | | | | | | | |
| | 043-023 | cd55 | 13.87 | 12.55 | | | | | | | | | | | |
| | | | | | | | | | | | | | | | |
| MPC-D100/755 | 045-001 | Cd12 | 43.57 | 53.84 | 59.39 | 24.81 | 175.67 | 12.72 | 30.31 | 10.90 | | | 29.67 | 94.47+ | 33.00 |
| | 045-002 | Cd13 | 44.93 | 54.51 | 56.32 | 24.15 | 186.94 | 11.92 | 30.13 | 12.13 | | | 26.69 | 101.39 | 31.00 |
| | 045-003 | Cd14 | 45.80 | 51.62 | 53.81 | 23.66 | 188.88 | 11.59 | 30.80 | 11.97 | | | 26.41 | 106.91 | 30.00 |
| | 045-004 | Cd15 | 46.41 | 51.56 | 53.25 | 22.55 | | 11.56 | 30.59 | | | | | 91.67+ | 29.00 |
| | 045-005 | Cd16 | 45.50 | 50.46 | 51.37 | 22.01 | 178.95 | 11.14 | 30.21 | 10.66 | | | 24.63 | 95.25 | 27.50 |
| | 045-006 | Cd17 | 45.36 | 49.32 | 49.56 | 21.81 | 176.41 | 11.11 | 29.80 | 12.71 | | | 25.21 | 96.57 | 26.50 |
| | 045-007 | Cd18 | 45.45 | 49.56 | 48.29 | 20.80 | 172.91 | 10.81 | 28.44 | 13.83 | | | 24.10 | 85.60 | 25.00 |
| | 045-008 | Cd19 | 45.44 | 47.93 | 47.24 | 20.49 | 166.37 | 11.23 | 30.34 | 13.11 | | | 24.26 | 84.71 | 24.00 |
| | 045-009 | Cd20 | 45.38 | 46.72 | 45.21 | 21.92 | 162.46 | 11.03 | 28.63 | 12.70 | | | 21.96 | 81.75 | 23.00 |
| | 045-010 | Cd21 | 43.71 | 46.17 | 43.13 | 20.36 | 157.42 | 9.71 | 27.07 | 12.59 | | | 21.31 | 77.99 | 23.00 |
| | 045-011 | Cd22 | 42.98 | 45.77 | 41.49 | 21.32 | 149.50 | 9.07 | 26.93 | 12.05 | | | 20.26 | 69.17 | 22.20 |
| | 045-012 | Cd23 | 44.23 | 42.29 | 41.59 | 21.02 | 140.46 | 8.99 | 25.27 | 10.57 | | | 20.48 | 68.84 | 23.00 |
| | 045-013 | Cd24 | 43.97 | 42.22 | 40.65 | 20.96 | 135.71 | 8.58 | 24.61 | 10.55 | | | 19.98 | 66.34 | 23.50 |
| | 045-014 | Cd25 | 43.62 | 41.02 | 41.29 | 20.49 | 126.77 | 8.76 | 24.65 | 9.73 | | | 17.91 | 59.01 | 24.50 |
| | 045-015 | Cd26 | 42.92 | 41.22 | 39.13 | 21.24 | 121.33 | 8.55 | 23.78 | 9.62 | | | 17.96 | 54.63 | 23.00 |
| | 045-016 | Cd27 | 42.59 | 39.54 | 37.37 | 20.55 | 114.02 | 7.36 | 22.73 | 8.66 | | | 17.01 | 47.39 | 22.00 |
| | 045-017 | Cd28 | 43.06 | 38.84 | 36.82 | 20.80 | | 7.66 | 21.84 | | | | | 40.29+ | 21.50 |
| | 045-018 | Cd29 | 42.25 | 37.14 | 35.65 | 19.85 | 102.79 | 7.36 | 21.36 | 6.61 | | | 13.46 | 46.70 | 22.00 |
| | 045-019 | Cd30 | 38.45 | 36.85 | 36.00 | 23.47 | 89.81 | 7.15 | 20.56 | 6.52 | | | 12.58 | 38.45 | 22.00 |
| | 045-020 | Cd31 | 37.77 | 36.51 | 36.68 | 22.30 | | 6.93 | 20.57 | | | | | 23.35+ | 21.00 |
| | 045-021 | Cd32 | 40.75 | 32.27 | 33.34 | 18.87 | 77.78 | 6.63 | 20.83 | 5.23 | | | 7.50 | 28.78 | 20.00 |
| | 045-022 | Cd33 | 40.16 | 30.51 | 31.67 | 18.70 | 74.15 | 6.76 | 20.79 | 4.40 | | | 8.15 | 26.12 | 20.00 |
| | 045-023 | Cd34 | 39.45 | 29.58 | 31.62 | 18.26 | 68.74 | 6.73 | 20.67 | 4.31 | | | 9.58 | 22.06 | 19.00 |
| | 045-024 | Cd35 | 38.64 | 27.85 | 31.95 | 18.47 | 61.98 | 5.95 | 20.91 | 5.47 | | | 8.34 | 18.28 | 20.00 |
| | 045-025 | Cd36 | 37.51 | 27.51 | 29.36 | 18.33 | 52.11 | 5.93 | 19.40 | 6.59 | | | 7.98 | 17.78 | 24.00 |
| | 045-026 | Cd37 | 36.82 | 26.53 | 28.98 | 18.04 | 49.78 | 5.86 | 19.36 | 7.81 | | | 6.95 | 14.46 | 22.00 |
| | 045-027 | Cd38 | 35.13 | 25.55 | 28.96 | 19.85 | 43.09 | 5.60 | 17.59 | 7.48 | | | 6.46 | 13.96 | 20.00 |
| | 045-028 | Cd39 | 34.58 | 24.23 | 29.38 | 19.19 | 43.38 | 6.06 | 17.40 | 7.05 | | | 6.70 | | |
| | 045-029 | Cd40 | 34.41 | 24.41 | 29.31 | 20.27 | | 5.57 | 16.70 | | | | | | |
| | 045-030 | Cd41 | 32.67 | 22.80 | 27.93 | 17.90 | | | | | | | | | |

**Scapula**

| Registered No. | Specimen No. | Position | Maximum length | Coracoid articulation width | Maximum height through the glenoid fossa rim and acromion process | Maximum distal height | Glenoid length | Minimum shaft height | Mediolateral width at glenoid in ventral view |
|---|---|---|---|---|---|---|---|---|---|
| | | | 1 | 2 | 3 | 4 | 5 | 6 | 7 |
| MPC-D100/746 | 036-036 | R | 325.00 | 44.65 | 79.48 | 75.36 | 43.89 | 40.98 | 22.81 |
| | 036-037 | L | 320.58 | 50.83 | 79.30 | 72.08 | 47.51 | 39.60 | 25.37 |
| MPC-D100/727 | 018-005 | L | | | 81.46 | | | 37.41 | |
| MPC-D100/750 | 040-010 | L | 195.20 | 26.41 | 42.03 | 38.41 | 23.18 | 23.79 | 11.57 |
| | 040-011 | R | | 28.63 | 38.95 | | 16.82 | 18.65 | 8.76 |
| | 040-012 | L | | | 52.24 | | 28.06 | 22.86 | 9.56 |
| MPC-D100/744 | 034-011 | R | 675.74 | 106.87 | 156.45 | 131.59 | 96.93 | 73.66 | 62.28 |

**Coracoid**

| Registered No. | Specimen No. | Position | Maximum length | Maximum width | Glenoid fossa length | Scapula articular width | Ventral scapular articulation width |
|---|---|---|---|---|---|---|---|
| | | | 1 | 2 | 3 | 4 | 5 |
| MPC-D100/746 | 036-036 | R | 64.37 | 66.92 | 22,42 | 44.11 | 33.69 |
| | 036-037 | L | 64.73 | 68.41 | 25.56 | 53.51 | 27.90 |
| MPC-D100/750 | 040-013 | R | 32.70 | 38.64 | 19.82 | 25.16 | 10.49 |

**Humerus**

| Registered No. | Specimen No. | Position | Maximum length | Maximum proximal width | Maximum distal width | Medial condyle width | Lateral condyle width | Width at the most lateral point of the distal deltopectoral crest | Deltopectoral crest length | Distance from the midpoint of the head to the lateral border | Distance from the midpoint of the head to the medial border | Minimum shaft width | Proximal width in craniolateral view | Minimum shaft width in craniolateral view | Width of lateral condyle in craniolateral view | Head height | Head width |
|---|---|---|---|---|---|---|---|---|---|---|---|---|---|---|---|---|---|
| | | | 1 | 2 | 3 | 4 | 5 | 6 | 7 | 8 | 9 | 10 | 11 | 12 | 13 | 14 | 15 |
| MPC-D100/746 | 036-040 | R | 208.65 | 68.70 | 52.48 | 30.44 | 27.91 | 47.54 | 90.81 | 47.75 | 43.62 | 27.34 | 34.75 | 22.55 | 27.18 | 33.95 | 29.23 |
| | 036-041 | L | 204.6 | 64.22 | 51.51 | 32.55 | 27.35 | 43.26 | 91.49 | 50.06 | 48.53 | 26.09 | 39.93 | 24.21 | 27.01 | 33.80 | 27.49 |
| MPC-D100/724 | 015-001 | L | | 70.91 | | | | 52.06 | 116.53 | 50.73 | 41.16 | 32.39 | | | | | |
| MPC-D100/736 | 026-001 | L | 234.55 | 66.50 | 49.06 | 30.34 | 29.58 | 40.13 | 97.78 | 39.16 | 43.49 | 26.69 | 34.84 | 23.32 | 28.81 | 34.80 | 26.69 |
| MPC-D100/750 | 040-014 | R | 135.54 | 36.69 | 29.41 | 17.71 | 17.46 | 26.45 | 53.70 | 23.23 | 18.99 | 16.28 | 16.20 | 14.55 | 15.49 | 16.31 | 15.92 |
| | 040-015 | R | 116.14 | 31.86 | 27.93 | 17.82 | 15.83 | 24.07 | 42.14 | 23.34 | 19.22 | 13.35 | 15.55 | 12.16 | 15.05 | 12.22 | 13.21 |
| | 040-016 | R | | | | 16.72 | | 32.40 | | | | 18.81 | | 14.23 | | | |
| | 040-017 | R | 121.23 | 31.78 | 29.56 | 17.46 | 16.25 | 24.10 | 46.65 | 20.24 | 17.45 | 14.28 | 15.24 | 11.33 | 13.45 | 10.61 | 12.83 |
| | 040-018 | L | 136.64 | 32.09 | 29.26 | 15.17 | 17.89 | 25.49 | 54.15 | 19.22 | 18.25 | 17.09 | 17.04 | 12.81 | 15.34 | 12.08 | 12.95 |
| | 040-019 | R | | | | | | | 51.43 | | | | | | | | |

**Sternum**

| Registered No. | Specimen No. | Position | Length, from the anteriolateral edge of the distal end of "rod" to the anterior margin of the blade | Length from the anteriolateral edge of the distal end of the rod to the posterior margin of the blade | Width of the distal end of the rod | Width of blade | Least width of the shaft |
|---|---|---|---|---|---|---|---|
| | | | 1 | 2 | 3 | 4 | 5 |
| MPC-D100/746 | 036-038 | R | 112.55 | 88.60 | 24.80 | 69.41 | 16.76 |
| | 036-039 | L | | | | 73.81 | 16.99 |
| MPC-D100/752 | 042-033 | R | | | | | |
| | 042-034 | L | | | | | |

**Ulna**

| Registered No. | Specimen No. | Position | Greatest length,from upper medial portion proximal end to upper lateral portion distal end | Minimum width of shaft | Greatest width of proximal end | Greatest width of distal end |
|---|---|---|---|---|---|---|
| | | | AB | CD | AE | BF |
| MPC-D100/746 | 036-042 | R | 191.86 | 21.04 | 43.24 | 25.81 |
| MPC-D100/722 | 013-001 | R | 224.83 | 21.62 | | 27.73 |
| MPC-D100/730 | 021-001 | R | | 23.22 | 44.40 | |
| MPC-D100/750 | 040-020 | L | 116.63 | 12.42 | 23.41 | 13.18 |
| | 040-020 | L | 116.42 | 11.73 | 25.65 | 13.48 |
| | 040-020 | R | 123.08 | 10.72 | 25.01 | 14.25 |
| **Radius** | | | | | | |
| Registered No. | Specimen No. | Position | Greatest length | Width of minimium of shaft | Greatest width of proximal end | Greatest width of distal end |
| | | | AB | CD | EF | GI |
| MPC-D100/746 | 036-043 | R | 186.87 | 15.93 | 30.6 | 27.76 |
| MPC-D100/750 | 040-023 | L | 129.35 | 13.05 | 17.75 | 18.28 |
| | 040-024 | R | | 9.36 | | 19.22 |
| **Metacarpal II** | | | | | | |
| Registered No. | Specimen No. | Position | Greatest length | Width of the proximal end | Width of the distal end | Minimum width of shaft |
| | | | 1 | 2 | 3 | 4 |
| MPC-D100/746 | 036-044 | R | 64.9 | 11.7 | 11.56 | 8.59 |
| MPC-D100/717 | 008-002 | R | | | | 21.20 |
| | | | | | | |
| **Metacarpal III** | | | | | | |
| Registered No. | Specimen No. | Position | Greatest length | Width of the proximal end | Width of the distal end | Minimum width of shaft |
| | | | 1 | 2 | 3 | 4 |
| MPC-D100/746 | 036-045 | R | 78.1 | 16.8 | 19.08 | 10.15 |
| | | | | | | |
| **Metacarpal IV** | | | | | | |

| Registered No. | Specimen No. | Position | Greatest length | Width of the proximal end | Width of the distal end | Minimum width of shaft |
|---|---|---|---|---|---|---|
| | | | 1 | 2 | 3 | 4 |
| MPC-D100/746 | 036-046 | R | 74.10 | 18.45 | 11.63 | 12.43 |
| | | | | | | |
| **Metacarpal V** | | | | | | |
| Registered No. | Specimen No. | Position | Greatest length | Width of the proximal end | Width of the distal end | Minimum width of shaft |
| | | | 1 | 2 | 3 | 4 |
| MPC-D100/746 | 036-047 | R | 28.10 | 16.60 | 11.28 | 9.09 |
| | | | | | | |
| **Digit II proximal phalanx (DII-Ph-1)** | | | | | | |
| Registered No. | Specimen No. | Position | Length median line | Width, proximal end | Height, proximal end | |
| | | | 1 | 2 | 3 | |
| MPC-D100/746 | 036-048 | R | 23.90 | 12.73 | 17.94 | |
| | 036-054 | L | 24.94 | 13.32 | 18.75 | |
| | | | | | | |
| **Digit II second phalanx (DII-Ph-2)** | | | | | | |
| Registered No. | Specimen No. | Position | Length median line | Width, proximal end | Height, proximal end | |
| | | | 1 | 2 | 3 | |
| MPC-D100/746 | 036-055 | L | 8.98 | 15.18 | 11.16 | |
| | | | | | | |
| | | | | | | |
| **Digit III proximal phalanx (DIII-Ph-1)** | | | | | | |

| Registered No. | Specimen No. | Position | Length median line | Width, proximal end | Height, proximal end |
|---|---|---|---|---|---|
| | | | 1 | 2 | 3 |
| MPC-D100/746 | 036-049 | R | 17.91 | 17.55 | 15.38 |
| | | | | | |
| **Digit III second phalanx (DIII-Ph-2)** | | | | | |
| Registered No. | Specimen No. | Position | Length median line | Width, proximal end | Height, proximal end |
| | | | 1 | 2 | 3 |
| MPC-D100/746 | 036-051 | R | 8.01 | 16.59 | 12.62 |
| | | | | | |
| **Digit IV proximal phalanx (DIV-Ph-1)** | | | | | |
| Registered No. | Specimen No. | Position | Length median line | Width, proximal end | Height, proximal end |
| | | | 1 | 2 | 3 |
| MPC-D100/746 | 036-050 | R | 18.70 | 13.49 | 16.42 |
| | | | | | |
| **Digit V proximal phalanx (DV-Ph-1)** | | | | | |
| | | | | | |
| Registered No. | Specimen No. | Position | Length median line | Width, proximal end | Height, proximal end |
| | | | 1 | 2 | 3 |
| MPC-D100/746 | 036-052 | R | 14.54 | 9.71 | 11.08 |
| | | | | | |
| | | | | | |
| **Digit V third phalanx (DV-Ph-3)** | | | | | |

| Registered No. | Specimen No. | Position | Length median line | Width, proximal end | Height, proximal end | | |
|---|---|---|---|---|---|---|---|
| | | | 1 | 2 | 3 | | |
| MPC-D100/746 | 036-053 | R | 9.04 | 7.23 | 6.71 | | |

**Ilium**

| Registered No. | Specimen No. | Position | Maximum length | Height | Length of postacetabular process | Height of postacetabular process | Angle preacetabular deflection |
|---|---|---|---|---|---|---|---|
| | | | (AB) | (CD) | (HA) | (EF) | (BCD) |
| MPC-D100/746 | 036-230 | R | 378.35 | 61.77 | 96.19 | 41.25 | 70° |
| | 036-231 | L | 379.47 | 63.15 | 93.4 | 34.08 | 75° |
| MPC-D100/752 | 042-047 | R | 458.00 | 95.53 | 84.00 | 47.99 | 75° |
| | 042-048 | L | 419.00 | 119.83 | 81.00 | 65.21 | 70° |
| MPC-D100/756 | 046-001 | R | | 106.86 | 88.03 | 40.46 | 70° |
| MPC-D100/761 | 050-001 | L | | 81.22 | | 33.91 | 70° |
| MPC-D100/750 | 040-027 | R | | 44. 30 | 40. 75 | 20. 10 | 70° |
| | 040-028 | R | | | | | |

**Ischum**

| Registered No. | Specimen No. | Position | Length, from the lowest point of the acetabular margin to the symptote of the ischial foot(AB) | Width of the ischial foot (CD) | Width of the shaft (EF) | Length of iliac peduncle (GH) | Length of obturator process, from the lowest point of the obturator foramen to the proximal tip (IJ) |
|---|---|---|---|---|---|---|---|
| | | | (AB) | (CD) | (EF) | (GH) | (IJ) |
| MPC-D100/746 | 036-228 | R | | | 27.96 | 61.78 | 21.98 |
| | 036-229 | L | | | 26.11 | 61.64 | |
| MPC-D100/726 | 017-001 | L | | | | | |
| MPC-D100/727 | 018-004 | R | | | | | |
| | 018-009 | L | | | | | |
| MPC-D100/752 | 042-049 | R | 460.00 | 93.26 | 32.66 | 89.82 | 32.42 |
| | 042-050 | L | | | 31.92 | 74.55 | 35.59 |
| MPC-D100/762 | 051-004 | R | 375.00 | 74.39 | 22.87 | 55.52 | 24.28 |
| MPC-D100/760 | 049-001 | L | 424.00 | 66.63 | 29.17 | 72.17 | |
| MPC-D100/750 | 040-029 | R | 178.34 | | 10.68 | | 20.25 |
| | 040-030 | R | 165.85 | | 13.84 | 30.19 | |
| | 040-031 | R | | | | | |

**Pubis**

| Registered No. | Specimen No. | Position | Length, line AF bisects the blade into dorsal and ventral segments. Point B is on the asymptote of the acetabulum. | Length of blade (AF) | Height of the blade (CD) | Minimum width of the neck (HI) |
|---|---|---|---|---|---|---|
| | | | | (AF) | (CD) | (HI) |
| MPC-D100/746 | 036-226 | R | 194.85 | 116.55 | 119.00 | 59.76 |
| | 036-227 | L | 188.83 | 116.94 | 127.29 | 58.83 |
| MPC-D100/752 | 042-051 | R | 297.32 | 139.48 | | 78.76 |
| | 042-052 | L | | | | 80.41 |

**Femur**

| Registered No. | Specimen No. | Position | Greatest length (AB) | Least diameter(CD) | Length of 4th trochanter | Length of distal part (from the distal base of 4th trochanter to the distal end of lateral |
|---|---|---|---|---|---|---|
| | | | 1 | 2 | 3 | 4 |
| MPC-D100/746 | 036-226 | R | 415.88 | 54.1 | 100.27 | 171.46 |
| | 036-227 | L | 408.75 | 46.46 | 101.94 | 158.62 |
| MPC-D100/718 | 009-002 | L | 574.00 | 55.47 | 173.03 | 192.22 |
| | 009-003 | R | | | | 178.26 |
| MPC-D100/721 | 012-001 | L | | | | |
| MPC-D100/731 | 022-001 | L | | 43.76 | 121.85 | 150.85 |
| MPC-D100/752 | 042-098 | R | 563.49 | 53.64 | 155.71 | 235.59 |
| MPC-D100/755 | 045-049 | R | 562.00 | 67.90 | 171.87 | 107.85 |
| MPC-D100/743 | 033-001 | R | 464.00 | 53.78 | 129.64 | 208.23 |
| MPC-D100/744 | 034-004 | L | 728.04 | 104.91 | 189.55 | 251.03 |
| MPC-D100/750 | 040-032 | R | 226.68 | 26.62 | 61.32 | 86.15 |
| | 040-033 | R | 209.43 | 24.25 | 62.30 | 76.63 |
| | 040-034 | L | 171.06 | 19.68 | 54.68 | 61.87 |
| | 040-035 | L | 185.25 | 27.48 | 68.64 | |

**Tibia**

| Registered No. | Specimen No. | Position | Greatest length | Width of the distal end | Least width of midshaft | Width of the caudal portion of the proximal end | Width of the cnemial crest |
|---|---|---|---|---|---|---|---|
| | | | 1 | 2 | 3 | 4 | 5 |
| MPC-D100/746 | 036-234 | R | 383.82 | 114.14 | 40.9 | 55.60 | 79.66 |
| | 036-235 | L | 390.78 | 111.68 | 40.4 | 55.40 | 69.36 |
| MPC-D100/712 | 003-001 | R | | 61.78 | 37.83 | 44.98 | |
| MPC-D100/718 | 009-001 | R | | 114.55 | 58.00 | | 57.95 |
| MPC-D100/722 | 013-002 | R | 309.38 | 60.27 | 30.18 | 53.05 | 37.50 |
| | 013-003 | L | | | 31.38 | | 51.76 |

| | | | | | | | |
|---|---|---|---|---|---|---|---|
| MPC-D100/725 | 016-001 | L | 434.00 | | 51.01 | 88.87 | |
| MPC-D100/734 | 024-001 | R | | 84.52 | 42.93 | | |
| MPC-D100/737 | 027-001 | L | | | | 81.90 | |
| MPC-D100/762 | 051-001 | R | 468.00 | 78.59 | 43.90 | 90.53 | 71.89 |
| | 051-005 | L | 478.76 | | 43.98 | 94.37 | 65.56 |
| MPC-D100/744 | 034-005 | L | 703.00 | 150.25 | 84.64 | 162.73 | |
| | 034-007 | R | 715.00 | 153.38 | 83.02 | 171.05 | 100.40 |
| MPC-D100/750 | 040-036 | R | 214.29 | 66.18 | 19.98 | 29.37 | 38.12 |
| | 040-037 | L | 224.25 | 66.51 | 22.97 | 31.5 | 41.06 |
| | 040-038 | R | 213.34 | 60.61 | 21.45 | 29.96 | 44.21 |
| | 040-040 | L | 191.25 | 54.91 | 18.55 | 29.9 | 38.35 |
| | 040-041 | R | 181.35 | 50.47 | 14.27 | 22.14 | 44.61 |
| | 040-042 | R | | | 15.87 | 24.30 | 40.32 |
| | 040-043 | L | | | 16.70 | 26.21 | 32.55 |
| | 040-044 | R | 208.33 | 55.10 | 17.32 | 27.28 | 40.18 |

**Fibula**

| Registered No. | Specimen No. | Position | Greatest length | Greatest width of the proximal end | Greatest width of the distal end | Least width of midshaft |
|---|---|---|---|---|---|---|
| | | | 1 | 2 | 3 | 4 |
| MPC-D100/746 | 036-236 | R | 360.38 | 60.59 | 37.21 | 17.28 |
| | 036-237 | L | 363.93 | 54.56 | 35.37 | 17.59 |
| MPC-D100/755 | 045-050 | R | 464.00 | 83.54 | 62.04 | 24.61 |
| MPC-D100/744 | 034-006 | L | 658.00 | 90.18 | 61.62 | 118.00 |
| | 034-008 | R | 658.00 | 103.77 | 74.54 | 115.00 |
| MPC-D100/750 | 040-045 | L | 194.32 | 29.09 | 24.36 | 35.00 |
| | 040-046 | R | 162.67 | 29.76 | 22.60 | 40.00 |

**Metatarsal II**

| Registered No. | Specimen No. | Position | Greatest length | Height of dorsal portion of the distal end | Height of ventral portion of the distal end | Ventral length, from heighest point of the proximal end to the caudal margin of the oblique articular surface in distal end | Height of the proximal end in lateral side | Length, from the proximal end to the flange in dorsolateral surface | Length, from the flange in dorsolateral surface to the caudal margin of the oblique articular surface in distal end | Width of the proximal end | Width of the distall end | Width of the midshaft |
|---|---|---|---|---|---|---|---|---|---|---|---|---|
| | | | 1 | 2 | 3 | 4 | 5 | 6 | 7 | 8 | 9 | 10 |
| MPC-D100/746 | 036-238 | R | 117.57 | 28.25 | 32.11 | 84.19 | 93.76 | 61.21 | 37.06 | 30.12 | 30.71 | 17.14 |
| | 036-241 | L | 119.87 | 30.2 | 33.43 | 88.66 | 98.55 | 63.04 | 38.1 | 28.21 | 30.38 | 17.03 |
| MPC-D100/754 | 044-082 | L | 157.32 | 40.84 | 55.04 | 105.45 | 143.38 | 79.91 | 46.63 | 45.36 | 41.06 | 28.2 |
| | | | | | | | | | | | | |
| MPC-D100/739 | 029-001 | L | 147.58 | 33.36 | 54.07 | 95.11 | 128.00 | 73.31 | 42.71 | 33.23 | | 24.13 |
| MPC-D100/741 | 031-001 | L | 152.26 | 39.01 | 43.43 | 116.02 | 118.28 | 73.42 | 46.01 | 34.36 | 35.89 | 23.76 |
| MPC-D100/750 | 040-049 | R | 74.64 | 13.60 | 18.97 | 60.02 | 41.00 | 44.57 | 19.27 | | | |
| | 040-050 | R | 66.93 | 11.37 | 12.97 | 52.81 | 37.00 | 37.42 | 21.31 | | | |
| | 040-051 | R | 68.74 | 10.64 | 13.65 | 50.10 | 39.00 | 36.46 | 19.09 | | | |
| MPC-D100/751 | 041-004 | L | 258.28 | 58.48 | 59.59 | 186.08 | 172.00 | 123.42 | 86.02 | 49.97 | 46.72 | 31.60 |

**Metatarsal III**

| Registered No. | Specimen No. | Position | Greatest length | Semicircle height of the proximal end in lateral view | Height of the proximal end in medial view | Height of the distal end in lateral view | Width of the distal end in dorsall view | Height of the distal end in medial view | Width of the distal end in ventrall view | Length from the asymptote of proximal end in lateral view to the dorsolateral margin of the oblique articular surface in the distal condyle | Length from the proximal end to the medial condyle in ventral view | Length from the proximal end to the asymptote of the lateral condyle in ventral view | Width of midshaft |
|---|---|---|---|---|---|---|---|---|---|---|---|---|---|
| | | | 1 | 2 | 3 | 4 | 5 | 6 | 7 | 8 | 9 | 10 | 11 |
| MPC-D100/746 | 036-239 | R | 142.83 | 123.29 | 72.13 | 34.44 | 45.78 | 42.65 | 38.34 | 131.14 | 139.62 | 137.30 | 27.77 |
| | 036-242 | L | 146.16 | 113.11 | 55.17 | 34.52 | 45.96 | 41.92 | 39.63 | 134.86 | 139.28 | 135.62 | 28.32 |
| MPC-D100/754 | 044-083 | L | 198.98 | 189.46 | 102.68 | 51.47 | 67.84 | 59.3 | 57.73 | 180.18 | 193.41 | 189.71 | 41.78 |
| MPC-D100/740 | 030-001 | R | 165.73 | 182.00 | 123.00 | 38.52 | 52.84 | 45.82 | 44.43 | 142.48 | 155.30 | 150.03 | 96.00 |

| | | | | | | | | | | | | | |
|---|---|---|---|---|---|---|---|---|---|---|---|---|---|
| MPC-D100/750 | 040-047 | R | 92.21 | 61.00 | 38.00 | 20.60 | 24.15 | 22.92 | 20.49 | 74.86 | 83.20 | 81.61 | 57.00 |
| | 040-048 | L | 78.87 | 59.00 | 34.00 | 18.49 | 22.03 | 22.60 | 17.62 | 71.38 | 72.89 | 69.90 | 49.00 |
| MPC-D100/751 | 041-005 | L | 293.10 | 354.00 | 184.00 | 68.87 | 98.44 | 90.52 | 85.41 | 260.80 | 247.14 | 272.76 | |

**Metatarsal IV**

| Registered No. | Specimen No. | Position | Greatest length | Length from the asymptote of distal end in medial view to the heighist point of the flange in dorsomedial surface | Length from the heighest point of the flange in dorsomedial surface to the asymptote of the proximal end in dorsomedial view | Length from the asymptote of the proximal end in ventromedial surface to the ventro-lateral condyle | Length from the heighest point of the flange in ventral view to the ventro-medial condyle | Width of the distal end in dorsal view | Width of the distal end in ventral view | Height of the distal end in lateral view | Height of the distal end in medial view | Length of proximal semicircle end | Height of proximal end in medial view |
|---|---|---|---|---|---|---|---|---|---|---|---|---|---|
| | | | 1 | 2 | 3 | 4 | 5 | 6 | 7 | 8 | 9 | 10 | 11 |
| MPC-D100/746 | 036-240 | R | 115.61 | 74.78 | 41.62 | 103.73 | 62.62 | 30.5 | 34.32 | 39.04 | 36.82 | 98.77 | 42.15 |
| | 036-243 | L | 118.23 | 73.3 | 42.27 | 106.58 | 60.01 | 30.48 | 34.15 | 36.94 | 35.03 | 98.6 | 41.28 |
| MPC-D100/754 | 044-084 | L | 165.08 | 100.03 | 70.72 | 154.24 | 91.89 | 48.52 | 51.36 | 52.87 | 50.43 | 139.43 | 67.36 |
| MPC-D100/750 | 040-052 | L | 73.14 | 46.70 | 27.94 | 64.03 | 36.81 | 13.59 | 17.81 | 18.35 | 18.04 | 69.00 | 21.00 |
| MPC-D100/751 | 040-006 | L | 231.90 | 128.73 | 93.84 | 221.23 | 123.26 | 57.89 | 56.97 | 74.25 | 67.06 | 205.00 | 141.00 |

**Proximal pedal phalanx of digit II**

| Registered No. | Specimen No. | Side | Greatest length | Least width of the midshaft | Width of the distal end in dorsal view | Height of the distal end in lateral view | Width of the distal end in ventral view | Height of the distal end in medial view | Height of the proximal end | Width of the proximal end in dorsal view | Width of the proximal end in ventral view | Height of the midshaft |
|---|---|---|---|---|---|---|---|---|---|---|---|---|
| | | | 1 | 2 | 3 | 4 | 5 | 6 | 7 | 8 | 9 | 10 |
| MPC-D100/746 | 036-244 | R | 46.93 | 25.68 | 16.84 | 26.74 | 35.06 | 25.52 | 35.18 | 35.01 | 32.26 | 23.08 |
| | 036-251 | L | 47.01 | 26.20 | 16.73 | 26.86 | 35.14 | 27.46 | 35.38 | 35.93 | 32.40 | 21.40 |
| MPC-D100/754 | 044-085 | L | 62.46 | 37.77 | 24.79 | 37.06 | 46.81 | 38.22 | 47.56 | 50.24 | 40.77 | 30.56 |
| MPC-D100/752 | 042-099 | L | 63.38 | 41.19 | 30.52 | | | 41.76 | | 46.66 | | 35.19 |
| MPC-D100/750 | 040-053 | L | 26.54 | 17.82 | 9.23 | 10.69 | 17.23 | 9.18 | 13.84 | 19.53 | 16.01 | 11.30 |

| | | | | | | | | | | | | |
|---|---|---|---|---|---|---|---|---|---|---|---|---|
| MPC-D100/751 | 041-007 | L | 65.13 | 40.49 | 27.47 | 43.17 | 49.82 | 42.95 | 63.89 | | 52.14 | 36.63 |

**Digit II median phalanx**

| Registered No. | Specimen No. | Side | Length in median line | Width of the distal end | Width of the proximal end in ventral view | Width of the proximal end in dorsal view | Height of the midshaft |
|---|---|---|---|---|---|---|---|
| | | | 1 | 2 | 3 | 4 | 5 |
| MPC-D100/746 | 036-245 | R | 16.74 | 23.13 | 29.39 | 18.15 | 17.59 |
| | 036-252 | L | 17.25 | 23.56 | 28.99 | 17.35 | 16.92 |
| MPC-D100/754 | 044-086 | L | 24.83 | 25.60 | 41.67 | 26.66 | 25.99 |
| MPC-D100/751 | 041-008 | L | 28.20 | 42.67 | | 34.07 | 32.43 |

**Digit II ungual phalanx**

| Registered No. | Specimens | Side | Length in median line | Greatest width of hoof-shaped part | Width of the proximal end | Height of the proximal end |
|---|---|---|---|---|---|---|
| | | | 1 | 2 | 3 | 4 |
| MPC-D100/746 | 036-246 | R | 44.27 | 29.45 | 23.57 | 19.62 |
| | 036-253 | L | 43.74 | 29.96 | 23.61 | 19.64 |
| MPC-D100/754 | 044-087 | L | 52.57 | 46.08 | 30.16 | 29.93 |
| MPC-D100/752 | 042-106 | R | 46.68 | 46.89 | 31.11 | 26.11 |
| | 042-107 | L | 47.71 | 45.86 | 32.09 | 29.08 |
| MPC-D100/751 | 041-009 | L | | | 46.03 | 32.04 |

**Proximal pedal phalanx of digit III**

| Registered No. | Specimen No. | Side | Greatest length | Least width of the midshaft | Height of the proximal end | Height of the midshaft | Height of the distal end in medial view | Width of the distal end in ventral view | Width of the proximal end in dorsal view |
|---|---|---|---|---|---|---|---|---|---|
| | | | 1 | 2 | 3 | 4 | 5 | 6 | 7 |
| MPC-D100/746 | 036-254 | L | 41.13 | 31.56 | 34.01 | 20.95 | 22.66 | 39.04 | 46.75 |
| MPC-D100/754 | 044-088 | L | 54.21 | 46.47 | 45.38 | 30.06 | 33.79 | 57.04 | 64.79 |

| | | | | | | | | | |
|---|---|---|---|---|---|---|---|---|---|
| MPC-D100/752 | 042-100 | R | 52.91 | 43.63 | 44.76 | 30.86 | 34.04 | 52.31 | 65.88 |
| MPC-D100/750 | 040-054 | L | 28.10 | 19.25 | 17.89 | 11.90 | 14.26 | 23.06 | 28.47 |
| MPC-D100/751 | 041-010 | L | 70.04 | 62.55 | 66.06 | 38.61 | 49.35 | 84.83 | 89.61 |
| | | | | | | | | | |
| | | | | | | | | | |
| | | | | | | | | | |
| **Digit III second phalanx** | | | | | | | | | |
| Registered No. | Specimen No. | Side | Length in median line | Width of the distal end | Width of the proximal end in ventral view | Width of the proximal end in dorsal view | Height of the midshaft | | |
| | | | 1 | 2 | 3 | 4 | 5 | | |
| MPC-D100/746 | 036-255 | L | 14.56 | 34.81 | 39.8 | 26.55 | 19.01 | | |
| MPC-D100/754 | 044-089 | L | 17.67 | 53.46 | 55.35 | 32.99 | 25.76 | | |
| MPC-D100/751 | 041-011 | L | 14.97 | 77.54 | 79.97 | 59.98 | | | |
| | | | | | | | | | |
| | | | | | | | | | |
| **Digit III third phalanx** | | | | | | | | | |
| Registered No. | Specimen No. | Side | Length in median line | Width of the distal end | Width of the proximal end in ventral view | Width of the proximal end in dorsal view | Height of the midshaft | | |
| | | | 1 | 2 | 3 | 4 | 5 | | |
| MPC-D100/746 | 036-247 | R | 10.32 | 32.31 | 32.78 | 19.84 | 18.93 | | |
| | 036-256 | L | 11.40 | 29.56 | 29.83 | 20.01 | 17.2 | | |
| MPC-D100/754 | 044-090 | L | 16.41 | 35.91 | 42.76 | 26.32 | 22.91 | | |
| MPC-D100/751 | 041-012 | L | 17.74 | 62.24 | 36.98 | 62.34 | 23.20 | | |
| | | | | | | | | | |
| | | | | | | | | | |
| **Digit III ungual phalanx** | | | | | | | | | |
| Registered No. | Specimen No. | Side | Length in median line | Greatest width of hoof-shaped part | Width of the proximal end | Height of the proximal end | | | |
| | | | 1 | 2 | 3 | 4 | | | |
| MPC-D100/746 | 036-248 | R | 39.52 | 38.29 | 27.43 | 18.36 | | | |
| | 036-257 | L | 38.46 | 37.84 | 27.40 | 18.87 | | | |
| MPC-D100/751 | 041-013 | L | 60.37 | | 55.00 | 27.54 | | | |
| MPC-D100/752 | 042-105 | R | 44.59 | 57.22 | 38.25 | 26.82 | | | |
| MPC-D100/754 | 044-091 | L | | 55.47 | 37.13 | 26.04 | | | |

**Proximal pedal phalanx of digit IV**

| Registered No. | Specimen No. | Side | Greatest length | Least width of the midshaft | Width of the proximal end in dorsal view | Height of the midshaft | Height of the proximal end | Height of the distal end in medial view | Height of the distal end in lateral view | Width of the distal end in ventral view |
|---|---|---|---|---|---|---|---|---|---|---|
| | | | 1 | 2 | 3 | 4 | 5 | 6 | 7 | 8 |
| MPC-D100/746 | 036-249 | R | 34.08 | 28.13 | 36.48 | 21.31 | 31.61 | 24.92 | 24.32 | 33.94 |
| | 036-258 | L | 34.08 | 27.02 | 35.81 | 20.43 | 30.62 | 24.09 | 24.25 | 33.49 |
| MPC-D100/750 | 040-055 | L | 20.46 | 15.94 | 19.28 | 13.59 | 17.60 | 14.32 | 12.18 | 16.84 |
| MPC-D100/751 | 041-014 | L | 53.52 | 45.38 | 57.70 | 42.32 | 60.53 | 44.13 | 46.59 | 57.14 |
| MPC-D100/752 | 042-101 | R | 43.18 | 38.44 | 46.5 | 30.85 | 43.44 | 36.22 | 33.52 | 45.71 |
| MPC-D100/754 | 044-092 | L | 45.68 | 36.88 | 48.97 | 31.35 | 43.64 | 35.28 | 34.10 | 47.94 |

**Digit IY, second phalanx**

| Registered No. | Specimen No. | Side | Length in median line | Width of the distal end | Width of the proximal end in ventral view | Width of the proximal end in dorsal view | Height of the midshaft |
|---|---|---|---|---|---|---|---|
| | | | 1 | 2 | 3 | 4 | 5 |
| MPC-D100/754 | 044-093 | L | 15.60 | 44.33 | 44.53 | 26.29 | 24.80 |
| MPC-D100/751 | 041-015 | L | 17.98 | 55.56 | 54.34 | 32.71 | 29.14 |

**Digit IY, third phalanx**

| Registered No. | Specimen No. | Side | Length in median line | Width of the distal end | Width of the proximal end in ventral view | Width of the proximal end in dorsal view | Height of the midshaft |
|---|---|---|---|---|---|---|---|
| | | | 1 | 2 | 3 | 4 | 5 |
| MPC-D100/746 | 036-259 | L | 8.29 | 21.54 | 23.48 | 15.23 | 16.46 |
| MPC-D100/754 | 044-094 | L | 11.46 | 37.82 | 38.86 | 20.87 | 22.89 |

| | | | | | | | |
|---|---|---|---|---|---|---|---|
| MPC-D100/752 | 042-102 | R | 12.63 | 37.78 | 35.78 | 19.12 | 21.02 |
| MPC-D100/751 | 041-016 | L | 8.80 | 44.95 | 44.78 | | 29.93 |

**Digit IY, fourth phalanx**

| Registered No. | Specimens | Side | Length in median line | Width of the distal end | Width of the proximal end in ventral view | Width of the proximal end in dorsal view | Height of the midshaft |
|---|---|---|---|---|---|---|---|
| | | | 1 | 2 | 3 | 4 | 5 |
| MPC-D100/746 | 036-260 | L | 9.88 | 18.32 | 22.56 | 15.32 | 15.68 |
| MPC-D100/754 | 044-095 | L | 11.83 | 27.31 | 31.77 | 19.90 | 20.75 |
| MPC-D100/751 | 041-017 | L | 11.67 | 37.66 | 40.96 | 28.74 | 24.50 |
| MPC-D100/752 | 042-102 | R | 10.06 | 30.64 | 28.80 | 16.34 | 19.74 |

**Digit IY, ungual phalanx**

| Registered No. | Specimen No. | Side | Length in median line | Greatest width of hoof-shaped part | Width of the proximal end | Height of the proximal end |
|---|---|---|---|---|---|---|
| | | | 1 | 2 | 3 | 4 |
| MPC-D100/746 | 036-250 | R | 40.14 | 27.48 | 20.38 | 17.93 |
| | 036-261 | L | 41.34 | 28.65 | 21.33 | 18.32 |
| MPC-D100/754 | 044-096 | L | | 43.33 | 27.47 | 27.32 |
| MPC-D100/751 | 041-018 | L | 69.88 | 57.58 | 46.17 | 31.46 |
