# Supplementary material for: A new hadrosauroid (Dinosauria: Ornithopoda) from the Late Cretaceous Baynshire Formation of the Gobi Desert (Mongolia)
Source: PLoS One. 2019 Apr 17;14(4):e0208480. doi: 10.1371/journal.pone.0208480 (PMC6469754; doi:10.1371/journal.pone.0208480)
Supplement: S1 Appendix — (DOCX) [file pone.0208480.s002.docx]

**S1 Appendix. Data matrix used in the phylogenetic analysis, based on Wu & Godefroit [11].**

*Hypsilophodon foxii* 0000000000000000000000?0000000000000000000000000000000000000000000000000000000000000000000?00000000000000000

*Zalmoxes* *shqiperorum* ?0000000000000000?00?0?000000000000010101000100000000000010100000000000?0000?000000?00?????0000121?000000??0

*Tenontosaurus tilleti* 100100010001000000000000001000000?0000000?010000000000000??10000000000010000000000010000000000000001000000?0

*Dryosaurus altus* 0001000100010000000000?0001000001?0010000101100000000000?10100010000000?001000000?010000?0?00001110100000?00

*Camptosaurus dispar* 110100010001000000000000001000001?00101001011000000000000?01000100000010001010000001011110000001110100000010

*Mantellisaurus atherfieldensis* 110100010001000000000001001010011000100001011000000000100001010100000021101110000101011121100001111110100111

*Iguanodon bernissartensis* 110100010001000000000001001010011000100001011000000000100001010100000021001110000101011121100001111110100111

*Ouranosaurus nigeriensis* 11010001000100000010000100101001100010000111111000000010000101010000002110???0000101011121?00000111110100111

*Bolong yixianensis* 11010??1000100??00???????1?01?011?0??0??00011000000???10???10101000000210?10000????1001111100?0011??????01?1

*Altirhinus kurzanovi* 110?000100011?0000???????210101110011000??011001000?0?10000111011000002??????0000101001?11?000001?1110100??1

*Koshisaurus katsuyama* ?????????????????????????11010001????????????????????????????00100?????????????????????????????????1?0?1????

*Jinzhousaurus yangi* 110?0??1000100010000000??2?01?????0010?????10?0000?????00001?1?11000????????????????????????????????????????

*Equijubus normani* 11010??1000110000?000?0??2?00??010001000???1110?00??011001011100000000?1?01????????????????00???????????????

*Batyrosaurus rozhdestvenskyi* ??????????????000?0000?10??????1?0011?0?1?0110010001111?0?0111012000012??????????10?0???11??????????????????

*Probractrosaurus gobiensis* 1101000?00010?000?0000010??01001110010?01?011001000111110?0111012100011??0???00001010?1?11?00000111110100??1

*Eolambia caroljonesa* ?101010?00010?????000?0?02??10011100?0?010???011000111??000111012100121?????????????0??????00??0?1????10????

*Protohadros byrdi* ?101010?0001000001???????210100111001010??0111110001111?010111012100120?????????????????????????????????????

*Shuangmiaosaurus* *gilmorei*

???????????????????????????010011????????????00100011?1?????21?121????0?????????????????????????????????????

Xuwulong *yueluni* 11?100010001100000?0010102?010?01?0010000?01100?000???10???1?0?110?????10000???????????????00000?11110??????

*Levnesovia transoxiana* ?????????????0000?0100010??01001120010?010???001???11?1?110??1012100120???????000???0??????00????????1?1???2

*Jeyawati rugoculus* ???????????????????????????0??011?????00???1?00100011?1?????21112000120?????????????????????????????????????

*Tethyshadros insularis* 11010?01000100000000000??21000????001010?????00101???1?111?12111110001?10110110??101022131210?00101111???1?2

Zhanghenglong *yangchengensis* ??????????????????????????10110?1?0110???????001?0121112?????11111011?0??????1??????????????????????????????

*Gilmoreosaurus mongoliensis* ?????????????????????????21000011?0110?0??0111?????11?11????211121001?0?0????1000???0??????0(01)10011?111211??2

*Bactrosaurus johnsoni* 1101010?000?00000000000102101001120110001001100100011111110121012100120?001??10001010????1?00000111111211??2

*Gobihadros mongoliensis* 111100010000000000000001021010011101100000011001000?11121111?1112100120100???1000101021121111000111111211112

*Plesiohadros djadokhtaensis* 1111100?00???00001000?0102101?0?1??11110101111010002111???11?1?11100120??????????????221311??????????????112

*Lophorhothon atopus* ??????1??01??000?1???0010?????????0??1?11??????????????????????12100110?0????????????????1???????0???????112

*Claosaurus agilis* ????????????????????????????????????????????????????1????????11121002201?0???1???1110??????0011??0????21?10?

*Telmatosaurus transsylvanicus* 1101010?00010?000?0000010210111?2?0?1?1010?1100100111222110021112100120?0????1?1??010????1??????1?111?21????

*Eotrachodon orientalis* 11111011001110000111?10?02101102220111?1???11001001211111111?2112100120?1??????????????????????????11???????

*Hadrosaurus foulkii* ??????????????????????????????1?????????????????????????????22112101220?????????????0??????1111010???121????

*Brachylophosaurus canadensis* 111211110011100001001101121001122211111110111201111212221111221121112201011011111101022131111110101111211112

*Edmontosaurus annectens* 111211110011100001001101121001122211111110111201111212221111221121112201011011111101022131111110101111211112

*Parasaurolophus cyrtocristatus* 111100011101011111110111022112122211111110111101111212221111221121112201111011111111122131111110111111211112

*Corythosaurus casuarius* 111100011101011111110111022112122211111110111101111212221111221121112201111011111111122131111110111111211112
